# Supplementary material for: Rates, causes, place and predictors of mortality in adults with intellectual disabilities with and without Down syndrome: cohort study with record linkage
Source: BMJ Open. 2020 May 17;10(5):e036465. doi: 10.1136/bmjopen-2019-036465 (PMC7239521; doi:10.1136/bmjopen-2019-036465)
Supplement: Supplementary data [file bmjopen-2019-036465supp001.pdf]

Rates, causes, place, and predictors of mortality in adults with intellectual disabilities with and without Down syndrome: cohort study with record linkage

Supplementary table 1. Previously reported standardised mortality ratios, causes, and risks for death

Author	Country	SMR (95% confidence interval)	Number of deaths	Causes of death and risk factors for death
Forsgren et al (1996)[7]	Sweden	4.2 (3.3, 5.3) at 20-59y; 1.1 (0.9, 1.5) at 60+y<br>*Without epilepsy:*<br>3.8 (2.8, 5.0) at 20-59y; 1.1 (0.8, 1.5) at 60+y<br>*With epilepsy:*<br>5.0 (2.9, 8.7) at 20-59y; 2.4 (0.9, 6.1) at 60+y<br>*With epilepsy and cerebral palsy:*<br>8.0 (4.1, 15.7) at 20-59y; 0.9 (0.1, 6.6) at 60+y<br>*M:* 1.6 (1.2, 2.0) at 0-60+y<br>*F:* 2.6 (2.0, 3.3) at 0-60+y<br>*Mild ID:* 1.8 (1.1, 2.7) at 0-60+y<br>*Moderate ID:* 1.5 (1.1, 2.0) at 0-60+y<br>*Severe ID:* 2.0 (1.5, 2.6) at 0-60+y<br>*Profound ID:* 8.1 (5.6, 11.7) at 0-60+y	124 at 0-60+y; 112 at 20-60+y	*Underlying cause at 0-60+y:*<br>Congenital anomalies: SMR=46.3 (32.9, 65.0)<br>Nervous system: SMR=9.7 (5.5, 17.0)<br>Mental disorder: SMR=4.0 (1.9, 8.4)<br>Respiratory: SMR=3.3 (2.0, 5.5)<br>Circulatory: SMR=2.1 (1.6, 2.7)<br>Violent death: SMR=1.4 (0.6, 2.8)<br>Neoplasm: SMR=0.9 (0.6, 1.6)
Durvasula & Beange (2002)[8]	Australia	4.9 (3.4, 6.4) at 10-59y<br>*M:* 4.1 (2.4, 5.9) at 10-59y<br>*F:* 6.2 (3.3, 9.1) at 10-59y	40 at 10-59y; 31 at 20-59y	*Underlying cause at 10-59y:*<br>Respiratory: 35% (pneumonia, aspiration)<br>External causes: 20%<br>Neoplasm: 17.5%<br>Heart disease: 15% (congenital heart disease 50%)<br>Gastrointestinal: 7.5% (ischaemic bowel, perforated peptic ulcer, post-operative peritonitis)<br>Seizure: 5%
Tyrer et al (2007)[9]	England	3.24 (2.93, 3.56) at 20-70+y<br>*M:* 2.86 (2.50, 3.26) at 20-70+y<br>*F:* 3.63 (3.12, 4.20) at 20-70+y<br>1.51 (1.23, 1.83) to 11.50 (8.14, 15.78) at 20-70+y<br>*M:* 1.39 (1.03, 1.82) to 8.83 (5.60, 13.25) at 20-70+y<br>*F:* 1.60 (1.18, 2.12) to 17.22 (9.64, 28.4) at 20-70+y<br>*With Down syndrome:* 7.60 at 20-70+y<br>*Without Down syndrome:* 2.70 at 20-70+y	409 at 20-70+y	Not reported
Patja et al (2008)[10]	Finland	*M:* 2.2 at 20-39y, 1.0 at 40-59y, 1.0 at 60+y<br>*F:* 1.4 at 20-39y, 1.1 at 40-49y, 1.0 at 60+y<br>*Mild ID:*<br>M: 1.6 at 20-39y, 1.0 at 40-59y, 1.0 at 60+y<br>F: 1.2 at 20-39y, 1.1 at 40-49y, 1.0 at 60+y	1,046 at 20-97y	*Underlying cause at 2-97y:*<br>Vascular: 36% (cardiac infarct 33%, cerebral infarct 33%, congenital heart disease 18%, pulmonary infarct 6%)<br>Respiratory: 22% (pneumonia 83%, COPD 11%)

Rates, causes, place, and predictors of mortality in adults with intellectual disabilities with and without Down syndrome: cohort study with record linkage

		Moderate ID:<br>M: 2.3 at 20-39y, 1.1 at 40-59y, 1.0 at 60+y<br>F: 1.5 at 20-39y, 1.1 at 40-49y, 1.0 at 60+y<br>*Severe ID:*<br>M: 2.6 at 20-39y, 1.2 at 40-59y, 1.0 at 60+y<br>F: 1.6 at 20-39y, 1.0 at 40-49y, 1.0 at 60+y<br>*Profound ID:*<br>M: 2.1 at 20-39y, 1.1 at 40-59y, 1.0 at 60+y<br>F: 1.3 at 20-39y, 1.2 at 40-49y, 1.0 at 60+y		Neoplasm: 11% (digestive 44%, respiratory 15%, urogenital, 12%)<br>Digestive: 7% (intestinal obstruction 25%, ulcer perforation 13%)<br>Accidents and poisonings: 7% (commonest was fatal fracture, then drowning)<br>Vascular, neoplasm, and accident causes were less common than sex-age-matched general population; Digestive were 2.5 times, Respiratory 2.6-5.8 times more common
Tyrer & McGother (2009)[11]	England	2.77 (2.53, 3.03) at 20+y<br>*M:* 2.28 (2.02, 2.56) at 20+y<br>*F:* 3.24 (2.83, 3.69) at 20+y	503 at 20+y	*Underlying cause at 20+y:*<br>Pneumonia: 13.1%, SMR=6.47 (5.00, 8.23)<br>Nervous system: 13.1%, SMR=16.30 (12.61, 20.74)<br>Other respiratory: 12.9%, SMR=4.64 (3.58, 5.91)<br>Ischaemic heart disease: 11.5%, SMR=1.49 (1.13, 1.92)<br>Neoplasm: 9.3%<br>Congenital anomalies: 9.1%, SMR=85.60 (62.67, 114.18)<br>Cerebrovascular disease: 7.8%, SMR=2.40 (1.71. 3.28)
Oullette-Kuntz et al (2015)[12]	Canada	2.5 (2.1, 2.9) at 0-60+y<br>*M:* 2.1 (1.7, 2.6) at 0-60+y<br>*F:* 3.0 (2.4, 3.8) at 0-60+y<br>*M:* 1.7 (1.3, 2.3) to 3.4 (2.3, 4.7) at 20-60+y<br>*F:* 2.1 (1.4, 2.9) to 6.1 (4.1, 8.6) at 20-60+y	172 at 0-60+y; 158 at 20-60+y	*Risk factors for death:*<br>Age, Down syndrome (OR=1.76 at 20-39y; OR=1.69 at 40-59y: OR=22.34 at 60+y), cerebral palsy (OR=2.39 at 20-39y; OR=0.93 at 40-59y: OR=0.50 at 60+y), blindness/low vision (OR not given), technological dependance/medical fragility (OR=11.96 at 20-39y; OR=7.28 at 40-59y: OR=3.42 at 60+y), wheelchair dependence (OR=5.96 at 20-39y; OR=2.89 at 40-59y: OR=2.56 at 60+y), mobility impairment without wheelchair dependence (OR not given), epilepsy (OR=1.83 at 20-39y; OR=1.80 at 40-59y: OR=1.09 at 60+y)
Florio & Troller (2015)[13]	Australia	2.48 (2.32, 2.64) at 0-85+y<br>3.15 (2.94, 3.38) at 5-69y<br>*M:* 2.52 (2.29, 2.77) at 5-69y<br>*F:* 4.26 (3.83, 4.74) at 5-69y	953 at 0-85+y; 831 at 15+y	Not reported
McCarron et al (2015)[14]	Republic of Ireland	3.85 (3.70, 4.00) at 0-80+y<br>*M:* 3.09 (2.93, 3.25) at 0-80+y<br>*F:* 4.90 (4.63, 5.17) at 0-80+y<br>2.71 (2.41, 3.04) to 6.09 (5.29, 6.96) at 20-80y<br>*M:* 2.50 (2.18, 2.86) to 4.50 (3.69, 5.44) at 20-80y<br>*F:* 2.71 (2.32, 3.14) to 10.07 (8.99, 13.10) at 20-80y	2,666 at 0-80+y; 2,394 at 20-80+y	Not reported

Rates, causes, place, and predictors of mortality in adults with intellectual disabilities with and without Down syndrome: cohort study with record linkage

Heslop & Glover (2015)[15]	England	Median 2.13 (interquartile range 1.09, 2.83) across geographic areas at 18-65+y	18-65+y	Not reported
Lauer & McCallion (2015)[16]	USA	*Intellectual and developmental disabilities*:*<br>1.19 at all ages, 2011<br>1.49 at 18+y, 2009	120,913 in 2009 at 18+y, 140,104 in 2011 at all ages	Not reported
Arvio et al (2016)[17]	Finland	*Mild ID:*<br>2.28 (2.18, 2.39) at 0-60+y<br>1.99 (1.85, 2.13) to 2.77 (2.36, 3.23) at 15-60+y<br>*M:* 2.01 (1.88, 2.14) at 0-60+y<br>*F:* 2.80 (2.60, 3.01) at 0-60+y<br>*Severe ID:*<br>3.41 (3.30, 3.52) at 0-60+y<br>2.07 (1.96, 2.19) to 8.77 (7.77, 9.87) at 15-60+y<br>*M:* 2.59 (2.48, 2.72) at 0-60+y<br>*F:* 5.24 (4.99, 5.50) at 0-60+y	5,171 at 0-60+y; 5,053 at 15-60y	Not reported
Hosking et al (2016)[5]	England	HR=3.62 (3.33, 3.93) at 18-84y<br>*M:* HR=3.30 (2.96, 3.68) at 18-84y<br>*F:* HR=4.10 (3.61, 4.66) at 18-84y<br>*With Down syndrome:* HR=9.21 (7.22, 11.76)<br>*Without Down syndrome:* HR=3.19 (2.92, 3.49)<br>*With epilepsy:* HR=6.04 (5.04, 7.24)<br>*Without epilepsy:* HR=3.18 (2.90, 3.50)<br>*With high level of support needs:* HR=4.77 (4.08, 5.59)<br>*Without high level of support needs:* HR=3.28 (2.98, 3.62)<br>*With autism:* HR=2.39 (1.45, 3.96)<br>*Without autism:* HR=3.66 (3.37, 3.98)<br>*In communal/shared homes:* HR=4.99 (4.36, 5.73)<br>*Not in communal/shared homes:* HR=3.05 (2.74, 3.30)	656 at 18-84y	*Underlying cause at 18-84y:*<br>Circulatory: 21.6%, HR=3.05 (2.56, 3.64)<br>Respiratory: 18.8% (pneumonia and aspiration pneumonia), HR=6.68 (5.38, 8.29)<br>Neoplasm: 14.9%, HR=1.44 (1.18, 1.76)<br>Nervous system: 11.6%, HR=13.79 (9.70, 19.62)<br>Digestive: 7.0%, HR=4.02 (2.92, 5.54)<br>Congenital anomalies: 6.9%, HR could not be estimated<br>Mental disorders: 5.3%, HR=7.99 (5.19, 12.31)<br>External causes: 4.1%, HR=1.85 (1.26, 2.71)<br>Genitourinary: 3.5%, HR=10.89 (6.09, 19.47)<br>Endocrine, nutritional, and metabolic: 2.0%, HR=5.38 (2.79, 10.07)<br>*Down syndrome:* Respiratory: 20.3% (or 42.4% if "Down syndrome" is excluded as an underlying cause of death)<br>*Avoidable deaths:*<br>37% amenable (23% controls), 19% preventable (40% controls)

Rates, causes, place, and predictors of mortality in adults with intellectual disabilities with and without Down syndrome: cohort study with record linkage

Lauer (2016)[18]	USA	Not reported	438 in 2012, 409 in 2013, at 18+y	*Major cause of death, 2012, 2013*<br>Heart disease: 16.0%, 13.7%<br>Neoplasm: 13.7%, 13.4%<br>Alzheimer disease: 13.0%-12.2% (48% in Down syndrome)<br>Aspiration pneumonia: 9.4%, 8.6%<br>Septicaemia: 10.0%, 8.6%<br>Chronic lower respiratory diseases: 4.6%, 6.6%<br>Unintentional injury: 4.8%, 3.2%
Troller et al (2017)[19]	Australia	1.3 (1.2, 1.5) at 20+y<br>4.0 (3.1, 5.2) at 20-44y<br>2.3 (2.0, 2.7) at 45-64y<br>1.0 (0.8, 1.20 at 65+y<br>*M:*1.4 (1.1, 1.6) at 20+y<br>*F:* 1.3 (1.1, 1.6) at 20+y	732 at 20-65+y	*Underlying cause at 20-65+y:*<br>Circulatory: 18%<br>Neoplasm: 18%<br>Nervous: 16%<br>Respiratory: 12%<br>Congenital anomaly: 11%<br>Injury and poisoning: 6%<br>Digestive: 5%<br>*Avoidable deaths:* 31%
Glover et al (2017)[6]	England	3.18 (2.94, 3.43) at 0-99y<br>*M:* 3.03 (2.73, 3.35) at 0-99y<br>*F:* 3.40 (3.02, 3.81) at 0-99y<br>1.6 (1.2, 2.1) to 7.8 (5.4, 11.1) at 18-99y<br>*M:* 1.5 (0.9, 2.2) to 6.6 (4.0, 10.1) at 18-99y<br>*F:* 1.7 (1.1, 2.4) to 11.6 (6.0, 20.2) at 18-99y	664 at 0-99y	*Underlying cause at 0-99y:*<br>Circulatory: 22.9% (ischaemic heart disease 37.5%, cerebrovascular 25.7%, thrombophlebitis 6.6%, cardiomyopathy 5.9%, PE 3.9%), SMR=2.8 (2.4, 3.3)<br>Respiratory: 17.2% (pneumonia 50.0%, pneumonitis 21.0%), SMR=4.9 (4.0, 5.9)<br>Neoplasm: 3.1% (digestive 36.8%, respiratory 13.8%, female genital tract 10.3%, lymphoid and haematopoietic 10.3%), SMR=1.1 (0.9, 1.4)<br>Nervous: 12.8%, SMR=9.8 (7.8, 12.1)<br>Congenital anomalies: 8.4%, SMR=72.9 (55.1, 94.7)<br>Digestive: 7.8%, SMR=4.0 (3.0, 5.2)<br>No ICD10 chapters had fewer than expected deaths<br>Other common single causes: dementia 33/664, epilepsy 26/664, cerebral palsy 23/664<br>*Avoidable deaths:*<br>44.7% (41.0%, 48.5%), mostly amenable<br>M: 50.9% (45.9%, 56.0%); F: 36.9% (31.5%, 42.5%)

COPD=chronic obstructive pulmonary disease; HR=hazard ratio; ID=intellectual disabilities; OR=odds ratio; PE=pulmonary embolism; SMR=standardised mortality ratio; y=years

*Includes some individuals with IQ>70

Rates, causes, place, and predictors of mortality in adults with intellectual disabilities with and without Down syndrome: cohort study with record linkage

Rates, causes, place, and predictors of mortality in adults with intellectual disabilities with and without Down syndrome: cohort study with record linkage

Supplementary table 2. Groupings of related causes of deaths

**Infectious diseases**	**ICD10**
**Infection**	
ENTEROCOLITIS DUE TO CLOSTRIDIUM DIFFICILE	A047
SEPSIS DUE TO STAPHYLOCOCCUS AUREUS	A410
SEPSIS, UNSPECIFIED	A419
BACTERIAL INFECTION, UNSPECIFIED	A499
SUBACUTE SCLEROSING PANENCEPHALITIS	A811
CHRONIC VIRAL HEPATITIS B WITHOUT DELTA-AGENT	B181
PULMONARY CANDIDIASIS	B371
NECROTISING FASCIITIS	M726
URINARY TRACT INFECTION, SITE NOT SPECIFIED	N390
**Neoplasms**	
**Gastrointestinal malignant neoplasms**	
MALIGNANT NEOPLASM OF PAROTID GLAND	C07
MALIGNANT NEOPLASM, OESOPHAGUS, UNSPECIFIED	C159
MALIGNANT NEOPLASM, STOMACH, UNSPECIFIED	C169
MALIGNANT NEOPLASM, CAECUM	C180
MALIGNANT NEOPLASM, SIGMOID COLON	C187
MALIGNANT NEOPLASM, COLON, UNSPECIFIED	C189
INTRAHEPATIC BILE DUCT CARCINOMA	C221
NEOPLASM OF UNCERTAIN OR UNKNOWN BEHAVIOUR, OTHER DIGESTIVE ORGANS	D377
**Other neoplasms**	
MALIGNANT NEOPLASM, LOWER LOBE, BRONCHUS OR LUNG	C343
MALIGNANT NEOPLASM, BRONCHUS OR LUNG, UNSPECIFIED	C349
MALIGNANT NEOPLASM, BREAST, UNSPECIFIED	C509
MALIGNANT NEOPLASM, ENDOMETRIUM	C541
MALIGNANT NEOPLASM OF OVARY	C56
MALIGNANT NEOPLASM, TESTIS, UNSPECIFIED	C629
MALIGNANT NEOPLASM, BLADDER, UNSPECIFIED	C679
MALIGNANT NEOPLASMS OF THYROID GLAND	C73
WALDENSTROM MACROGLOBULINAEMIA	C880
NON-HODGKIN'S LYMPHOMA, UNSPECIFIED	C859
MALIGNANT NEOPLASM OF UNSPECIFIED SITE	C80
NEOPLASM OF UNCERTAIN OR UNKNOWN BEHAVIOUR, TRACHEA, BRONCHUS AND LUNG	D381
SECONDARY MALIGNANT NEOPLASM OF LUNG	C780
SECONDARY MALIGNANT NEOPLASM OF LIVER AND INTRAHEPATIC BILE DUCT	C787
SECONDARY MALIGNANT NEOPLASM OF BRAIN AND CEREBRAL MENINGES	C793
SECONDARY MALIGNANT NEOPLASM OF OTHER SPECIFIED SITES	C798
**Endocrine and metabolic diseases**	
**Diabetes**	
INSULIN-DEPENDENT DIABETES MELLITUS WITHOUT COMPLICATIONS	E109
NON-INSULIN-DEPENDENT DIABETES MELLITUS WITH RENAL COMPLICATIONS	E112
NON-INSULIN-DEPENDENT DIABETES MELLITUS W. PERIPHERAL CIRC. COMPLICATIONS	E115
NON-INSULIN-DEPENDENT DIABETES MELLITUS WITHOUT COMPLICATIONS	E119
UNSPECIFIED DIABETES MELLITUS WITH RENAL COMPLICATIONS	E142

UNSPECIFIED DIABETES MELLITUS WITHOUT COMPLICATIONS	E149
ABNORMAL GLUCOSE TOLERANCE TEST	R730
HYPERGLYCAEMIA, UNSPECIFIED	R739
**Metabolic disorders**	
OTHER HYPERPHENYLALANINAEMIAS	E701
DISORDERS OF PHOSPHORUS METABOLISM & PHOSPHATASES	E833
DISORDERS OF PLASMA-PROTEIN METABOLISM, NOT ELSEWHERE CLASSIFIED	E880

Mental disorders

**Dementias**	
VASCULAR DEMENTIA, UNSPECIFIED	F019
UNSPECIFIED DEMENTIA	F03
ALZHEIMER'S DISEASE WITH LATE ONSET	G301
ALZHEIMER'S DISEASE, UNSPECIFIED	G309
**Mental health**	
MENTAL AND BEHAVIOURAL DISORDERS DUE TO ACUTE INTOXICATION WITH ALCOHOL	F100
MENTAL AND BEHAVIOURAL DISORDERS DUE TO ALCOHOL DEPENDENCE SYNDROME	F102
MENTAL AND BEHAVIOURAL DISORDERS DUE TO USE OF TOBACCO, UNSPECIFIED	F179
SCHIZOPHRENIA, UNSPECIFIED	F209
BIPOLAR AFFECTIVE DISORDER, UNSPECIFIED	F319
OTHER & UNSPEC SYMPTOMS & SIGNS INVOLVING COGNITIVE FUNCTIONS & AWARENESS	R418
INTENTIONAL SELF-HARM BY JUMPING FROM A HIGH PLACE	X80
**Intellectual disabilities**	
UNSPECIFIED MENTAL RETARDATION	F79
DEVELOPMENTAL DISORDER OF SCHOLASTIC SKILLS, UNSPECIFIED	F819

Nervous system

**Epilepsies**	
GENERALIZED IDIOPATHIC EPILEPSY AND EPILEPTIC SYNDROMES	G403
EPILEPSY, UNSPECIFIED	G409
STATUS EPILEPTICUS, UNSPECIFIED	G419
MYOTONIC DISORDERS	G711
OTHER AND UNSPECIFIED CONVULSIONS	R568
**Cerebral palsy**	
SPASTIC QUADRAPLEGIC CEREBRAL PALSY	G800
SPASTIC HEMIPLEGIC CEREBRAL PALSY	G802
OTHER CEREBRAL PALSY	G808
CEREBRAL PALSY, UNSPECIFIED	G809
TETRAPLEGIA, UNSPECIFIED	G825
**Other neurological conditions**	
SEQUELAE OF INFLAMMATORY DISEASES OF CENTRAL NERVOUS SYSTEM	G09
PARKINSON'S DISEASE	G20
MYONEURAL DISORDER, UNSPECIFIED	G709
ENCEPHALITIS, MYELITIS AND ENCEPHALOMYELITIS, UNSPECIFIED	G049
ANOXIC BRAIN DAMAGE, NOT ELSEWHERE CLASSIFIED	G931
BLINDNESS, BINOCULAR	H540
OTHER DISORDERS OF NERVOUS SYSTEM, NOT ELSEWHERE CLASSIFIED	G98

Rates, causes, place, and predictors of mortality in adults with intellectual disabilities with and without Down syndrome: cohort study with record linkage

Circulatory system

**Acute myocardial infarction**	
ACUTE MYOCARDIAL INFARCTION, UNSPECIFIED	I219
CARDIAC ARREST, UNSPECIFIED	I469
**Other ischaemic heart disease**	
HYPERTENSIVE HEART DISEASE WITHOUT (CONGESTIVE) HEART FAILURE	I119
ACUTE ISCHAEMIC HEART DISEASE, UNSPECIFIED	I249
ATHEROSCLEROTIC HEART DISEASE	I251
CHRONIC ISCHAEMIC HEART DISEASE, UNSPECIFIED	I259
ATHEROSCLEROSIS OF AORTA	I700
GENERALIZED AND UNSPECIFIED ATHEROSCLEROSIS	I709
**Heart failure**	
HEART FAILURE, UNSPECIFIED	I509
LEFT VENTRICULAR FAILURE	I501
CONGESTIVE HEART FAILURE	I500
**Other cardiovascular disease**	
PULMONARY EMBOLISM WITHOUT MENTION OF ACUTE COR PULMONALE	I269
OTHER SPECIFIED PULMONARY HEART DISEASES	I278
PULMONARY HEART DISEASE, UNSPECIFIED	I279
AORTIC (VALVE) STENOSIS	I350
ATRIAL FIBRILLATION AND FLUTTER	I48
VENTRICULAR FIBRILLATION AND FLUTTER	I490
OTHER ILL-DEFINED HEART DISEASES	I518
PULMONARY OEDEMA	J81
CARDIOGENIC SHOCK	R570
PERIPHERAL VASCULAR DISEASE, UNSPECIFIED	I739
PHLEBITIS AND THROMBOPHLEBITIS OF OTHER DEEP VESSELS OF LOWER EXTREMITIES	I802
EMBOLISM AND THROMBOSIS OF OTHER SPECIFIED VEINS	I828
ACUTE AND SUBACUTE INFECTIVE ENDOCARDITIS	I330
ACUTE ENDOCARDITIS, UNSPECIFIED	I339
ENDOCARDITIS, VALVE UNSPECIFIED	I38
DILATED CARDIOMYOPATHY	I420
CARDIOMEGALY	I517
ESSENTIAL (PRIMARY) HYPERTENSION	I10
**Stroke**	
INTRACEREBRAL HAEMORRHAGE, UNSPECIFIED	I619
CEREBRAL INFARCTION DUE TO THROMBOSIS OF PRECEREBRAL ARTERIES	I630
CEREB INFARCT DUE TO UNSPEC OCCL/STENOSIS OF PRECEREB ARTERIES	I632
CEREBRAL INFARCTION, UNSPECIFIED	I639
STROKE, NOT SPECIFIED AS HAEMORRHAGE OR INFARCTION	I64
CEREBROVASCULAR DISEASE, UNSPECIFIED	I679
SEQUELAE OF STROKE, NOT SPECIFIED AS HAEMORRHAGE OR INFARCTION	I694
SEQUELAE OF OTHER AND UNSPECIFIED CEREBROVASCULAR DISEASES	I698

Respiratory system

**Respiratory infection**	
ACUTE UPPER RESPIRATORY INFECTION, UNSPECIFIED	J069
INFLUENZA WITH PNEUMONIA, OTHER INFLUENZA VIRUS IDENTIFIED	J100
INFLUENZA WITH OTHER RESP MANIFESTATIONS, OTHER INFLUENZA VIRUS IDENTIFIED	J101

Rates, causes, place, and predictors of mortality in adults with intellectual disabilities with and without Down syndrome: cohort study with record linkage

PNEUMONIA DUE TO STREPTOCOCCUS PNEUMONIAE	J13
BRONCHOPNEUMONIA, UNSPECIFIED	J180
LOBAR PNEUMONIA, UNSPECIFIED	J181
HYPOSTATIC PNEUMONIA, UNSPECIFIED	J182
PNEUMONIA, UNSPECIFIED	J189
UNSPECIFIED ACUTE LOWER RESPIRATORY INFECTION	J22
CHRONIC OBSTRUCTIVE PULMONARY DISEASE WITH ACUTE LOWER RESP INFECTION	J440
**Aspiration/reflux/choking**	
PNEUMONITIS DUE TO FOOD AND VOMIT	J690
GASTRO-OESOPHAGEAL REFLUX DISEASE WITHOUT OESOPHAGITIS	K219
INHALATION AND INGESTION OF FOOD CAUSING OBSTRUCTION OF RESPIRATORY TRACT	W79
FOREIGN BODY IN RESPIRATORY TRACT, PART UNSPECIFIED	T179
INHALATION/INGESTION OF OTHER OBJECTS CAUSING OBSTRUCT OF RESP TRACT	W80
**Other respiratory disorders**	
UNSPECIFIED CHRONIC BRONCHITIS	J42
EMPHYSEMA, UNSPECIFIED	J439
CHRONIC OBSTRUCTIVE PULMONARY DISEASE, UNSPECIFIED	J440
ASTHMA, UNSPECIFIED	J459
BRONCHIECTASIS	J47
OTHER INTERSTITIAL PULMONARY DISEASES WITH FIBROSIS	J841
PLEURAL EFFUSION, NOT ELSEWHERE CLASSIFIED	J90
CHRONIC RESPIRATORY FAILURE	J961
RESPIRATORY FAILURE, UNSPECIFIED	J969
OTHER SPECIFIED RESPIRATORY DISORDERS	J988
DYSPNOEA	R060
RESPIRATORY ARREST	R092
ASPHYXIATION	T71
UNSPECIFIED THREAT TO BREATHING	W84

Digestive system

**Ulcer/gastrointestinal perforation**	
OESOPHAGITIS	K20
PERFORATION OF INTESTINE (NONTRAUMATIC)	K631
PERITONITIS, UNSPECIFIED	K659
GASTRIC ULCER, CHRONIC OR UNSPECIFIED WITH PERFORATION	K255
OTHER PERITONITIS	K658
ACUTE PERITONITIS	K650
GASTROINTESTINAL HAEMORRHAGE, UNSPECIFIED	K922
ULCER OF INTESTINE	K633
**Other gastrointestinal disorders**	
BARRETTS OESOPHAGUS	K227
DIAPHRAGMATIC HERNIA WITHOUT OBSTRUCTION OR GANGRENE	K449
OTHER SPECIFIED NONINFECTIVE GASTROENTERITIS AND COLITIS	K528
ACUTE VASCULAR DISORDERS OF INTESTINE	K550
VASCULAR DISORDER OF INTESTINE, UNSPECIFIED	K559
VOLVULUS	K562
OTHER AND UNSPECIFIED INTESTINAL OBSTRUCTION	K566
CONSTIPATION	K590

MEGACOLON, NOT ELSEWHERE CLASSIFIED	K593
ACUTE AND SUBACUTE HEPATIC FAILURE	K720
OTHER AND UNSPECIFIED CIRRHOSIS OF LIVER	K746
AUTOIMMUNE HEPATITIS	K754
INFLAMMATORY LIVER DISEASE, UNSPECIFIED	K759
OTHER SPECIFIED DISEASES OF LIVER	K768
CALCULUS OF GALLBLADDER WITH OTHER CHOLECYSTITIS	K801
CHOLANGITIS	K830
ACUTE PANCREATITIS, UNSPECIFIED	K859
PSEUDOCYST OF PANCREAS	K863
INTESTINAL MALABSORPTION, UNSPECIFIED	K909
DYSPHAGIA	R13

Genitourinary system

Renal failure

CHRONIC NEPHRITIC SYNDROME, UNSPECIFIED	N039
OTHER ACUTE RENAL FAILURE	N178
ACUTE RENAL FAILURE, UNSPECIFIED	N179
END-STAGE RENAL DISEASE	N180
CHRONIC KIDNEY DISEASE, STAGE 5	N185
CHRONIC KIDNEY DISEASE, UNSPECIFIED	N189
UNSPECIFIED KIDNEY FAILURE	N19

Chromosomal abnormalities

Down syndrome

DOWN'S SYNDROME, UNSPECIFIED	Q909

Other congenital condition

CONGENITAL HYDROCEPHALUS, UNSPECIFIED	Q039
SPINA BIFIDA, UNSPECIFIED	Q059
CONGENITAL MALFORMATION OF HEART, UNSPECIFIED	Q249
CONGENITAL DEFORMITY OF SPINE	Q675
CONGEN MALFORMATION SYNDROMES PREDOMINANTLY ASSOCIATED WITH SHORT STATURE	Q871
MARFAN'S SYNDROME	Q874
OTHER SPECIFIED CONGEN MALFORMATION SYNDROMES, NOT ELSEWHERE CLASSIFIED	Q878
CONGENITAL MALFORMATION, UNSPECIFIED	Q899
KLINEFELTER'S SYNDROME, UNSPECIFIED	Q984
FRAGILE X CHROMOSOME	Q992
OTHER LACK OF EXPECTED NORMAL PHYSIOLOGICAL DEVELOPMENT	R628

Other conditions occurring with small frequency

Other condition

DECUBITUS ULCER AND PRESSURE AREA	L89
SCOLIOSIS, UNSPECIFIED	M419
URETHRAL STRICTURE, UNSPECIFIED	N359
EPISTAXIS	R040
IMMOBILITY	R263
MALAISE AND FATIGUE	R53
GENERALIZED ENLARGED LYMPH NODES	R591

Rates, causes, place, and predictors of mortality in adults with intellectual disabilities with and without Down syndrome: cohort study with record linkage

INSUFFICIENT INTAKE OF FOOD AND WATER DUE TO SELF NEGLECT	R636
OTHER SPECIFIED GENERAL SYMPTOMS AND SIGNS	R688
OTHER ILL-DEFINED AND UNSPECIFIED CAUSES OF MORTALITY	R99
EXPOSURE TO UNSPECIFIED FACTOR	X59
MULTI-SYSTEM DEGENERATION	G903
BENIGN NEOPLASM, MENINGES, UNSPECIFIED	D329
AGRANULOCYTOSIS	D70
SARCOIDOSIS OF OTHER AND COMBINED SITES	D868
SARCOIDOSIS, UNSPECIFIED	D869
HYPOPITUITARISM	E230
HYPOTHYROIDISM, UNSPECIFIED	E039
OTHER THYROTOXICOSIS	E058
VOLUME DEPLETION	E86

Injuries and external causes

Injuries and accidents

INTRACRANIAL INJURY, UNSPECIFIED	S069
UNSPECIFIED INJURY OF HEAD	S099
INJURY OF COLON	S365
FRACTURE OF NECK OF FEMUR	S720
FRACTURE OF SHAFT OF TIBIA	S822
UNSPECIFIED MULTIPLE INJURIES	T07
FAT EMBOLISM (TRAUMATIC)	T791
SEQUELAE OF UNSPECIFIED INJURY OF HEAD	T909
UNSPECIFIED FALL	W19
SEQUELAE OF OTHER ACCIDENTS	Y86

Medical/surgical complication

POISONING BY OTHER ANTIDYSRHYTHMIC DRUGS, NOT ELSEWHERE CLASSIFIED	T462
ADVERSE EFFECTS OF OTHER ANTIDYSRHYTHMIC DRUGS, NOT ELSEWHERE CLASSIFIED	Y522
ABN REACT TO/LATER COMPLIC OF OP WITH IMPLANT OF ARTIFICIAL INTERN DEVICE	Y831
ABN REACT TO/LATER COMPLIC OF OP WITH ANASTOMOSIS, BYPASS OR GRAFT	Y832
ABN REACT TO/LATER COMPLIC OF OP WITH FORMATION OF EXTERNAL STOMA	Y833
ABNORMAL REACTION TO OR LATER COMPLICATION OF OTHER MEDICAL PROCEDURES	Y848
SEQ OF PROCED CAUSING ABN REACT/COMPLIC,W/O MENTION OF MISADV AT THE TIME	Y883
OTHER POSTPROCEDURAL RESPIRATORY DISORDERS	J958

Rates, causes, place, and predictors of mortality in adults with intellectual disabilities with and without Down syndrome: cohort study with record linkage

Supplementary table 3. Predictors of the outcome time to death from univariate analyses

Variable		N with event/ N in group	Hazard ratio (95% CI)	Individual p-value	Overall p-value
Demographics					
Age at time of health assessment		294/961	1.05 (1.04, 1.06)	<0.0001	
Sex	Male	154/525	0.88 (0.70, 1.11)	0.2730	
	Female	140/436	1.00 (-)		
Ability level	Mild ID	92/382	1.00 (-)		0.0007
	Moderate ID	73/236	1.38 (1.01, 1.87)	0.0411	
	Severe ID	67/180	1.75 (1.28, 2.40)	0.0005	
	Profound ID	62/163	1.77 (1.28, 2.45)	0.0005	
Type of accommodation	Family carer	70/374	1.00 (-)		<0.0001
	Independent of care	36/93	2.35 (1.57, 3.52)	<0.0001	
	Paid support	161/435	2.18 (1.65, 2.88)	<0.0001	
	Congregate	27/59	2.87 (1.84, 4.48)	<0.0001	
Neighbourhood deprivation	1 – most affluent	18/73	1.00 (-)		0.1890
	2	56/137	1.92 (1.13, 3.27)	0.0158	
	3	10/45	0.90 (0.42, 1.95)	0.7896	
	4	10/40	1.06 (0.49, 2.30)	0.8808	
	5	12/32	1.71 (0.82, 3.55)	0.1527	
	6	9/32	1.27 (0.57, 2.82)	0.5640	
	7	9/34	1.09 (0.49, 2.43)	0.8302	
	8	15/58	1.21 (0.61, 2.41)	0.5818	
	9	35/124	1.22 (0.69, 2.16)	0.4882	
	10 - most deprived	120/386	1.41 (0.86, 2.31)	0.1782	
Civil status	Single	288/938	1.28 (0.57, 2.87)	0.5485	
	Not single	6/23	1.00 (-)		
Employment/day activities	Yes	83/231	1.33 (1.03, 1.71)	0.0284	
	No	211/730	1.00 (-)		
Smoker	Yes	46/101	1.70 (1.24, 2.33)	0.0009	
	No	248/860	1.00 (-)		
**Health**					
Down syndrome	Yes	64/179	1.30 (0.98, 1.71)	0.0673	
	No	230/782	1.00 (-)		
Epilepsy	Yes	111/325	1.25 (0.99, 1.58)	0.0636	
	No	183/636	1.00 (-)		
Spastic quadriplegia	Yes	24/325	1.67 (1.10, 2.54)	0.0158	
	No	183/636	1.00 (-)		
Impaired mobility	Yes	195/735	0.51 (0.40, 0.65)	<0.0001	
	No	99 /226	1.00 (-)		
Body mass index	Underweight	9/43	0.63 (0.32, 1.25)	0.1847	0.1865
	Acceptable	83/265	1.00 (-)		
	Overweight	75/289	0.78 (0.57, 1.06)	0.1132	
	Obese	81/237	1.08 (0.80, 1.47)	0.6152	
	Morbidly obese	16/58	0.87 (0.51, 1.48)	0.6058	
Hearing impairment	Yes	112/267	1.79 (1.41, 2.26)	<0.0001	
	No	182/694	1.00 (-)		
Visual impairment	Yes	154/449	1.29 (1.02, 1.62)	0.0317	
	No	140/512	1.00 (-)		

Rates, causes, place, and predictors of mortality in adults with intellectual disabilities with and without Down syndrome: cohort study with record linkage

Urinary incontinence	Yes	158/632	0.52 (0.41, 0.65)	<0.0001	
	No	136/329	1.00 (-)		
Bowel incontinence	Yes	197/733	0.55 (0.43, 0.70)	<0.0001	
	No	97/228	1.00 (-)		
Diabetes	Yes	29/47	2.72 (1.86, 4.00)	<0.0001	
	No	265/914	1.00 (-)		
PEG/tube fed	Yes	N/7	4.99 (2.22, 11.20)	0.0001	
	No	288/954			
Constipation	Yes	112/316	1.34 (1.06, 1.70)	0.0145	
	No	182/645	1.00 (-)		
Ataxia/gait disorder	Yes	104/276	1.50 (1.18, 1.90)	0.0009	
	No	190/685	1.00 (-)		
Nail disorder	Yes	74/223	1.18 (0.91, 1.54)	0.2120	
	No	220/738	1.00 (-)		
Epidermal thickening	Yes	66/207	1.10 (0.84, 1.45)	0.4947	
	No	228/754	1.00 (-)		
Cerebral palsy	Yes	54/175	1.02 (0.76, 1.37)	0.8792	
	No	240/786	1.00 (-)		
Osteoporosis	Yes	76/174	1.71 (1.32, 2.22)	<0.0001	
	No	218/786	1.00 (-)		
Fungal infection	Yes	42/158	0.83 (0.61, 1.18)	0.3366	
	No	252/803	1.00 (-)		
Hypertension	Yes	56/146	1.36 (1.01, 1.82)	0.0399	
	No	238/815	1.00 (-)		
Dysphagia	Yes	51/132	1.51 (1.11, 2.04)	0.0080	
	No	243/829	1.00 (-)		
Dyspnoea	Yes	49/130	1.41 (1.04, 1.92)	0.0285	
	No	245/831	1.00 (-)		
Muskuloskeletal pain	Yes	48/148	1.14 (0.83, 1.55)	0.4153	
	No	246/813	1.00 (-)		
Bone deformity	Yes	50/139	1.32 (0.97, 1.79)	0.0769	
	No	244/822	1.00 (-)		
Dental/oral problem	Yes	38/120	1.07 (0.76, 1.50)	0.7128	
	No	256/841	1.00 (-)		
Eczema/dermatitis	Yes	38/138	0.86 (0.61, 1.21)	0.3790	
	No	256/823	1.00 (-)		
GORD	Yes	51/133	1.43 (1.06, 1.94)	0.0198	
	No	243/828	1.00 (-)		
Lower respiratory tract infection	Yes	55/126	1.75 (1.30, 2.34)	0.0002	
	No	239/835	1.00 (-)		
Total number of physical conditions		294/961	1.06 (1.04, 1.08)	<0.0001	
Psychosis	Yes	11 /42	0.81 (0.44, 1.48)	0.4990	
	No	283 /919	1.00 (-)		
Affective disorder including bipolar	Yes	24/68	1.19 (0.78, 1.80)	0.4216	
	No	270/893	1.00 (-)		
Autism	Yes	13/69	0.54 (0.31, 0.94)	0.0306	
	No	281/892	1.00 (-)		
Problem behaviour	Yes	71/218	1.09 (0.83, 1.42)	0.5251	
	No	223/743	1.00 (-)		
Eating disorder, including pica	Yes	5/17	0.99 (0.41, 2.40)	0.9857	
	No	289/944	1.00 (-)		
Any mental illness, excluding problem behaviours	Yes	73/217	1.16 (0.89, 1.51)	0.2849	
	No	221/744	1.00 (-)		
Service use					
Number of GP consultations in last 12 months		287/951	1.05 (1.03, 1.06)	<0.0001	

Number of A&E attendances in last 12 months		280/938	1.09 (0.99, 1.20)	0.0847	
Number of health professions providing care		294 /961	1.10 (1.03, 1.16)	0.0023	
Prescriptions					
Antipsychotics	Yes	79/226	1.12 (0.94, 1.57)	0.1421	
	No	215/735	1.00 (-)		
Antidepressants	Yes	39/118	1.16 (0.83, 1.63)	0.3778	
	No	255/843	1.00 (-)		
Anxiolytic/hypnotics	Yes	20/68	0.95 (0.60, 1.49)	0.8159	
	No	274/893	1.00 (-)		
Antiepileptics	Yes	90/253	1.31 (1.02, 1.68)	0.0315	
	No	204/708	1.00 (-)		
Number of drug classes taken		294/961	1.16 (1.12, 1.21)	<0.0001	

A&E=accident and emergency; CI=confidence interval; GORD=gastro-oesophageal reflux disorder; PEG=percutaneous endoscopic gastrostomy
