# Supplementary material for: Rates, causes, place and predictors of mortality in adults with intellectual disabilities with and without Down syndrome: cohort study with record linkage
Source: BMJ Open. 2020 May 17;10(5):e036465. doi: 10.1136/bmjopen-2019-036465 (PMC7239521; doi:10.1136/bmjopen-2019-036465)
Supplement: Author's manuscript [file bmjopen-2019-036465.draft_revisions.pdf]

BMJ Open is committed to open peer review. As part of this commitment we make the peer review history of every article we publish publicly available.

When an article is published we post the peer reviewers' comments and the authors' responses online. We also post the versions of the paper that were used during peer review. These are the versions that the peer review comments apply to.

The versions of the paper that follow are the versions that were submitted during the peer review process. They are not the versions of record or the final published versions. They should not be cited or distributed as the published version of this manuscript.

BMJ Open is an open access journal and the full, final, typeset and author-corrected version of record of the manuscript is available on our site with no access controls, subscription charges or pay-per-view fees (http://bmjopen.bmj.com).

If you have any questions on BMJ Open's open peer review process please email info.bmjopen@bmj.com

BMJ Open

Rates, causes, place, and predictors of mortality in adults with intellectual disabilities with and without Down syndrome: cohort study with record linkage

Journal:	*BMJ Open*
Manuscript ID	bmjopen-2019-036465
Article Type:	Original research
Date Submitted by the Author:	16-Dec-2019
Complete List of Authors:	Cooper, Sally-Ann; University of Glasgow, Institute of Health and Wellbeing<br>Allan, Linda; University of Glasgow, Institute of Health and Wellbeing<br>Greenlaw, Nicola; University of Glasgow, Robertson Centre for Biostatistics, Institute of Health and Wellbeing<br>Mcskimming, Paula; University of Glasgow, Robertson Centre for Biostatistics, Institute of Health and Wellbeing<br>Jasilek, Adam; University of Glasgow, Robertson Centre for Biostatistics, Institute of Health and Wellbeing<br>Henderson, Angela; University of Glasgow, Institute of Health and Wellbeing<br>McCowan, Colin ; University of St. Andrews<br>Kinnear, Deborah; University of Glasgow, Institute of Health and Wellbeing<br>Melville, Craig; University of Glasgow, Institute of Health and Wellbeing
Keywords:	PUBLIC HEALTH, Adult psychiatry < PSYCHIATRY, EPIDEMIOLOGY, GENERAL MEDICINE (see Internal Medicine)

SCHOLARONE™
Manuscripts

I, the Submitting Author has the right to grant and does grant on behalf of all authors of the Work (as defined in the below author licence), an exclusive licence and/or a non-exclusive licence for contributions from authors who are: i) UK Crown employees; ii) where BMJ has agreed a CC-BY licence shall apply, and/or iii) in accordance with the terms applicable for US Federal Government officers or employees acting as part of their official duties; on a worldwide, perpetual, irrevocable, royalty-free basis to BMJ Publishing Group Ltd ("BMJ") its licensees and where the relevant Journal is co-owned by BMJ to the co-owners of the Journal, to publish the Work in this journal and any other BMJ products and to exploit all rights, as set out in our licence.

The Submitting Author accepts and understands that any supply made under these terms is made by BMJ to the Submitting Author unless you are acting as an employee on behalf of your employer or a postgraduate student of an affiliated institution which is paying any applicable article publishing charge ("APC") for Open Access articles. Where the Submitting Author wishes to make the Work available on an Open Access basis (and intends to pay the relevant APC), the terms of reuse of such Open Access shall be governed by a Creative Commons licence – details of these licences and which Creative Commons licence will apply to this Work are set out in our licence referred to above.

Other than as permitted in any relevant BMJ Author's Self Archiving Policies, I confirm this Work has not been accepted for publication elsewhere, is not being considered for publication elsewhere and does not duplicate material already published. I confirm all authors consent to publication of this Work and authorise the granting of this licence.

For peer review only

Rates, causes, place, and predictors of mortality in adults with intellectual disabilities with and without Down syndrome: cohort study with record linkage

*Sally-Ann Cooper

Linda Allan

Nicola Greenlaw

Paula McSkimming

Adam Jasilek

Angela Henderson

Colin McCowan

Deborah Kinnear

Craig Melville

*Corresponding author: Sally-Ann.Cooper@glasgow.ac.uk

For peer review only

Abstract

Objectives

To investigate mortality in adults with intellectual disabilities: rates, causes, place, demographic and clinical predictors.

Design

Cohort study and record linkage to death data.

Setting

General community.

Participants

961/1,023 (94%) adults (16-83 years; mean=44.1 years; 54.6% male) with intellectual disabilities, clinically examined in 2001-2004; subsequently record-linked to their National Health Service number, allowing linkage to death certificate data, 2018.

Outcome measures

Standardised mortality ratios (SMRs), underlying, and all contributing causes of death, avoidable deaths, place, and demographic and clinical predictors of death.

Results

294/961 (30.6%) had died; 64/179 (35.8%) with Down syndrome, 230/783 (29.4%) without Down syndrome. SMR overall=2.24 (1.98, 2.49); Down syndrome adults=5.28 (3.98, 6.57), adults without Down syndrome=1.93 (1.68, 2.18); male=1.69 (1.42, 1.95), female=3.48 (2.90, 4.06). SMRs decreased as age increased. More severe intellectual disabilities increased SMR, but ability was not retained in the multivariate model. SMRs were higher for most ICD-10 chapters. For adults without Down syndrome, aspiration/reflux/choking, and respiratory infection were the commonest underlying causes of mortality; for Down syndrome adults “Down syndrome”, and dementia were most common. Amenable deaths (29.8%) were double that in the general population (14%). 60.3% died in hospital. Mortality risk related to: percutaneous endoscopic gastrostomy/tube fed, Down syndrome, diabetes, lower respiratory tract infection at

For peer review only

cohort-entry, smoking, epilepsy, hearing impairment, increasing number of prescribed drugs, increasing age. Bowel incontinence reduced mortality risk.

Conclusions

Adults with intellectual disabilities with and without Down syndrome have different SMRs and causes of death which should be separately reported. They die younger, from different causes than other people. Some mortality risks are similar to other people, with earlier mortality reflecting more multi-morbidity; amenable deaths are also common. This should inform actions to reduce early mortality, e.g. training to avoid aspiration/choking, pain identification to address problems before they are advanced, and reasonable adjustments to improve health-care quality.

Strengths and limitations of this study

- Thorough methods of case ascertainment for intellectual disabilities at baseline.
- Individual verification of intellectual disabilities and its severity, and detailed health assessments at baseline.
- Longitudinal design.
- Large cohort size and study duration, and successful record linkage for 94% of participants.
- Limitations include that the study was conducted in only one part of Scotland, and the reliance upon recorded cause of death from death certificates.

Word count: 5,052

For peer review only - http://bmjopen.bmj.com/site/about/guidelines.xhtml

Introduction

People with intellectual disabilities die at a younger age than other people; on average, 20 years younger,[1] or 28 years younger specifically for people with Down syndrome.[2] It has been demonstrated that people with intellectual disabilities receive poorer management of their long term conditions within primary health care services compared with the general population,[3] and it is conceivable that this is one contributor to earlier mortality. It has been suggested that as many as 40% of deaths of people with intellectual disabilities may have been amenable to good quality health care.[4-6]

Previous studies on death in people with intellectual disabilities had limitations such as small sample sizes, or non-representative populations. More recently, there have been large-scale studies which are more representative, having been drawn from intellectual disabilities registers, or social security or primary care data with record linkage to death certification. These have been undertaken in parts of Sweden, Australia, England, Finland, Canada, Ireland, and USA (supplementary table 1).[5-18] These studies fairly consistently report standardised mortality ratios (SMRs) to be high for people with intellectual disabilities, more so at younger ages, and higher for women than men. Adult studies have tended to report SMRs in the region of 2-4, although in some, SMR is only slightly above 1.[10,18] However, direct comparison between studies is not always possible, due to the different age ranges studied and methods of reporting.

- Supplementary table 1 -

In view of the methods that studies have used for population identification (typically, routine administrative data linked to death certifications), they provide little information on the socio-clinical factors that influence SMR, or the risk factors associated with death, beyond that of age and sex. Three studies reported SMR by level of intellectual disabilities, with, broadly speaking, higher SMR with more severe intellectual

For peer review only - http://bmjopen.bmj.com/site/about/guidelines.xhtml

disabilities.[7,10,16] Only three studies (different studies to those that reported on level of intellectual disabilities) were able to report data separately for adults with intellectual disabilities with and without Down syndrome; two found higher mortality rates for adults with Down syndrome (SMR=7.6,[9] and hazard ratio=9.21[5]) than for adults without Down syndrome, or an odds ratio showing Down syndrome as a risk of death.[12] A further study reported SMR=5.5 for children and adults (combined) with Down syndrome, but did not report SMR for those with intellectual disabilities without Down syndrome.[19] Two studies reported adults with intellectual disabilities to have higher SMRs if they have the co-morbidities of epilepsy,[5,7] and cerebral palsy,[7] as opposed to not having these comorbidities. One study reported adults with intellectual disabilities with comorbid autism to have lower hazard ratios than those without comorbid autism.[5] One study reported the risk factors for mortality in a population with intellectual disabilities to be: age, Down syndrome, cerebral palsy, blindness/low vision, technological dependence/medical fragility, wheelchair dependence, mobility impairment without wheelchair dependence, and epilepsy.[12] Factors not found to be risks, if any, were not reported, and a further limitation was that factors were reported by agency staff, rather than the individuals undergoing health assessments.[12] We have not identified any other studies that investigated risk factors for time to mortality in adults with intellectual disabilities.

There is less consistency regarding the most common certified underlying causes of death in adults with intellectual disabilities, partly as some studies do not report these separately for children and adults, or by age ranges. Pneumonia, other respiratory diseases, and diseases of the nervous system were reported to be the most common in one study,[11] diseases of the circulatory system and respiratory systems in another,[5] heart disease, neoplasm, and Alzheimer disease in a third,[17] and diseases of the circulatory system, neoplasm, and the nervous system in a fourth.[18] In adults with intellectual disabilities, cause specific SMRs have been reported to be high across most groups of disorders.[5,11] These studies did not report cause of death separately for adults

For peer review only - http://bmjopen.bmj.com/site/about/guidelines.xhtml

with and without Down syndrome. Given the different health profile of people with Down syndrome compared with people with intellectual disabilities of other causes, this is an important limitation.[20] In people with Down syndrome, most studies on mortality have been conducted with child populations. The most common causes of death in people with Down syndrome have been reported to be congenital heart disease, and pneumonia/diseases of the respiratory system.[2]

Overall, the existing body of literature on mortality in adults with intellectual disabilties does not include more detailed information on level of intellectual disabilities, nor separate out the population with, from those without, Down syndrome (for whom causes of death may differ), nor investigate health and demographic predictors of death other than age and sex, and is inconsistent with regards to causes of death. A better understanding of these factors may provide a pathway to action to reduce the observed earlier mortality in adults with intellectual disabilities.

This study aims to investigate the rates, causes, place, and demographic and clinical associations with mortality in adults with intellectual disabilities, with and without Down syndrome.

Methods

Approval

Ethical approved was gained from NHS Greater Glasgow Primary Care Trust - Community & Mental Health Research Ethics Committee, and NHS Greater Glasgow and Clyde Safe Haven. Individual consent to participate was taken in line with Scottish law, between 2001-2004.

For peer review only

Participants

The adult (aged 16+ years) intellectual disabilities population living within the NHS Greater Glasgow area was identified through multiple sources between 2000-2001. General practitioners were financially incentivised to identify their registered patients with intellectual disabilities, and all 631 (100%) did so. Adults were also identified via the intellectual disabilities health and social work services including day services, the Health Board register, and records of financial payments for any service by social work. This process led initially to an over-identification, such as people with IQ scores in the 70–80 range with additional complex health needs. All were systematically reviewed by nurses in the intellectual disabilities health service, and this group were removed. Thus, a register was compiled, and subsequently updated annually via general practices, with central support from the intellectual disabilities health service, until 2017 when services were redesigned. The identified adult prevalence of intellectual disabilities within the area was 3.33 per 1,000.

Process and data collection

With initial piloting in 2001, each participant had a detailed assessment of their general and mental health, and demographic factors, completed 2002-2004. One of six specially trained, registered nurses reviewed each person's primary health care records, then used a semi-structured tool, the C21st Health Check, to assess clinical factors and the level and cause of intellectual disabilities. In addition to a review of existing health problems and all bodily health systems, a physical examination was undertaken, including assessment of vision and hearing, measurement of height and weight, and a phlebotomy protocol followed. All information was then reviewed by the nurse with one of three general practitioners with a special interest in intellectual disabilities, and any further investigations that were indicated were completed. Previously known, and newly identified, conditions were then classified using the *International Statistical Classification of Diseases and Related Health Problems, 10th Revision (ICD-10).*[21] Anyone identified to have possible, probable, or definite mental ill-health, autism, or problem behaviours was

For peer review only - http://bmjopen.bmj.com/site/about/guidelines.xhtml

then fully assessed by the project's intellectual disabilities psychiatrists. Each person's assessment findings were then case conferenced by the two Consultant psychiatrists, and diagnoses were derived and agreed according to clinical diagnoses, *ICD-10 (Diagnostic Criteria for Research),*[22] *Diagnostic and Statistical Manuel of Mental Disorders-IV-TR,*[23] and *Diagnostic Criteria for Psychiatric Disorders for use with Adults with Learning Disabilities (DC-LD).*[24] Information was also collected on demographics, and community, hospital, and social service use. Further details are provided elsewhere.[25,26] The data were entered into a database by two dedicated data-entry staff.

Each person in Scotland is given a number unique to them at birth or first registration with a general practitioner, which is used in almost all subsequent health service encounters, and on certification of death. The numbers are held on the Community Health Index (CHI) database at National Services Scotland. These CHI numbers provided a means to record link each participant with National Records for Scotland death certification data. This linkage was performed in 2018, and the linked data were held in the NHS Greater Glasgow & Clyde (NHS GG&C) Safe Haven. Data on immediate, underlying, and contributory causes of deaths by ICD-10 codes, age at death, and place of death were extracted.

In order to provide finer granularity of cause of death, and in view of the recognised issue of variation between health staff in distinguishing and recording immediate, two clinical academics then grouped individual causes of death into narrower groupings than those provided by ICD-10 chapter headings (supplementary table 2).

- Supplementary table 2 –

Analyses

All statistical analyses were conducted using R for Windows v3.3.0 or SAS 9.3 (SAS Institute, Cary NC) and were performed within the NHS GG&C Safe Haven environment.

For peer review only - http://bmjopen.bmj.com/site/about/guidelines.xhtml

Due to disclosure principles of the Safe Haven, results with counts of less than 5 cannot be released; these have been referred to as <5 throughout. Similarly, if is deemed possible that participants may be identified from the results, these may be omitted. Details are provided if this occurred.

Data were summarised for the population of adults aged 16+ years with intellectual disabilities. Categorical variables were summarised with the number and percentages of people falling into each category and the number of missing data. Continuous variables were summarised with the number of observations and those missing, the mean and standard deviation (SD), and the minimum and maximum values, unless otherwise stated.

Participant characteristics were summarised overall and for those alive and those deceased. For those who are deceased, their data including age at death, underlying/contributing causes of death, and location of death were summarised for those with and without Down syndrome. Location codes for place of death are provided where available. We have assumed that those with the code for non-institutional location to have died at home. Due to small numbers, location codes have been grouped together for NHS hospitals, home, and other hospitals/care facilities including hospices.

Mortality incidence rates have been calculated using the number of deaths in the cohort divided by the number of person years alive within the study period multiplied by 100,000, overall and for those with and without Down syndrome. Standard mortality ratios were calculated using population data for those aged 15 and over within NHS GG&C in 2010.[27,28] Death rates for males and females by 5 year band ages groups spanning from 15-20 years old to 90 years and over were summed to form the expected death rates for the general population. The observed death rate for adults with intellectual disabilities was taken from our study results. The observed/expected death rates were calculated for the intellectual disabilities cohort overall then separately by age

For peer review only - http://bmjopen.bmj.com/site/about/guidelines.xhtml

group, sex, ability level, and for the adults with, and without, Down syndrome, and ICD-10 chapter for cause of death, and compared to the general population.

Deaths were also analysed for those that could be considered as deaths that would have been avoidable. The Office for National Statistics (ONS) published a definition of avoidable mortality,[29] which lists the causes of amenable deaths (deaths that should not occur in the presence of good health care), and causes of preventable deaths, by ICD-10 codes. Causes of death for the adults with intellectual disabilities have been summarised by ONS definition of avoidable deaths.

To determine the demographic and clinical factors associated with death in adults with intellectual disabilities, time to event analyses were explored using univariate Cox Proportional Hazards models. Variables were selected as potentially relevant on the basis of what is known on causes of death in people with intellectual disabilities, the 20 most common physical health conditions reported in the adult population with intellectual disabilities,[20] and other factors hypothesised as potentially clinically relevant (supplementary table 3):

- Demographics - 9 variables.
- Clinical conditions - 33 variables.
- Service use - 3 variables.
- Prescriptions - 5 variables.

These models were then extended consider a single multivariable analysis and using a stepwise regression method, a model of the factors most associated with death was identified. Results from the univariate Cox Proportional Hazards models (Supplementary table 3) and the multivariable model from the stepwise results have been presented with hazard ratios with corresponding 95% confidence intervals (HR, 95% CI) and p-values were obtained.

- Supplementary table 3 -

For peer review only - http://bmjopen.bmj.com/site/about/guidelines.xhtml

Patient and public involvement

This study was designed to respond to the growing concern expressed by people with intellectual disabilities, their families, and third sector organisations about the early deaths of people with intellectual disabilities. The Scottish Learning Disabilities Observatory, where this research was undertaken, has a specific remit for people with intellectual disabilities. Its steering group includes partners from third sector organisations, including Down syndrome Scotland, and people with intellectual disabilities, who approved the work plan for this project prior to it commencing. Results from this study will be disseminated for people with intellectual disabilities in an easy-read version via the Scottish Learning Disabilities Observatory.

Results

Population characteristics

962 of the original 1,023 (94.0%) adults with intellectual disabilities were linked to a CHI number enabling the extraction of relevant death data. Reasons for the unlinked 61 people could be due to moving out of the area, or a recording mistake. 1 further participant was removed from the analysis due to inaccurate recording of dates, leaving 961 adults in the cohort (93.9%). Of these 961 adults, 294 (30.6%) had a record of death. Table 1 shows the baseline characteristics of the full cohort of 961, the adults who died, and those still alive at the time of linkage.

- Insert table 1 about here –

Age at death, and mortality incidence

The mean age at death was 61.0 years (SD=7.0 years). Of the 961 adults, 64 (35.8%) of the 179 adults with Down syndrome, and 230 (29.4%) of the 782 adults without Down syndrome had a record of death. Their mean age of death was 56.9 years (SD=4.3 years) for the adults with Down syndrome, and 62.2 years (SD=7.5 years) for

For peer review only - http://bmjopen.bmj.com/site/about/guidelines.xhtml

the adults without Down syndrome. Mortality incidence for the cohort during the study period was 3,049.0 per 100,000 person years follow-up, with 3,832.1 per 100,000 for those with Down Syndrome and 2,885.0 for those without Down syndrome.

Standardised mortality ratios

Compared with the general population, the SMR was 2.24 (1.98, 2.49) overall; 5.28 (3.98, 6.57) for adults with Down syndrome, 1.93 (1.68, 2.18) for adults without Down syndrome; 1.69 (1.42, 1.95) for men and 3.48 (2.90, 4.06) for women. SMRs were higher the more severe the level of intellectual disabilities, with people with profound intellectual disabilities having an SMR of 4.14 (3.11, 5.17). SMR was high for all age groups (though for the 15-25 year age group, the wide confidence interval includes one, perhaps due to the smaller number of deaths in this group); this decreased as age increased. SMRs were high for most ICD-10 chapter groups of conditions, particularly so for congenital malformations at 17.26 (10.75, 23.78), diseases of the digestive system at 16.13 (8.23, 24.04), mental and behavioural disorders at 12.64 (3.27, 22.00), and external causes at 11.08 (3.40, 18.76). Details are shown in table 2.

- Insert table 2 about here –

Causes of death

Cause of death data was available from death certificates for 262 (89.1%) of 294 participants who had died, which include 57 (89.1%) participants with Down syndrome, and 205 (88.7%) participants without Down syndrome. Table 3 shows the underlying causes of death by ICD-10 chapters separately for the adults with, and without Down syndrome. For the whole cohort, diseases of the respiratory system were the most common (21.8%), then diseases of the circulatory system (19.1%), then diseases of the nervous system (13.0%), and neoplasms, followed by congenital anomalies (10.3%). For the adults with Down syndrome, congenital anomalies were the most common (in all cases this was a record of “Down syndrome”), then jointly diseases of the respiratory

For peer review only - http://bmjopen.bmj.com/site/about/guidelines.xhtml

system and diseases of the circulatory system, then diseases of the nervous system, followed by infections, and mental and behavioural disorders. For the adults without Down syndrome, diseases of the respiratory system were the most common, then diseases of the circulatory system, then neoplasms, then diseases of the nervous system, followed by diseases of the digestive system. Table 4 presents the most common underlying causes of death by individual causes, or related groups of causes, with finer granularity than ICD-10 chapter headings (groups are shown in supplementary table 2). Causes are listed in the order of how common they were in the whole cohort. Data are presented separately for the adults with, and without Down syndrome. For the whole cohort, the most common cause was aspiration/reflux/choking, then respiratory infection, then other malignancy (non gastrointestinal), then other condition (mostly unrelated conditions that could not be reported individually or as groups, due to individually occurring at a frequency of <5). For the adults with Down syndrome, Down syndrome was the most common cause, then dementia, then other infection. For the adults without Down syndrome, aspiration/reflux/choking was the most common cause, then respiratory infection, then other malignancy (non gastrointestinal).

- Insert tables 3 and 4 about here -

Table 5 shows the all contributing causes of death data, again presenting the most common causes by individual causes, or related groups of causes with finer granularity than ICD-10 chapter headings. Data is presented separately for the adults with, and without Down syndrome. For the whole cohort, respiratory infection was the most common cause (27.1%), followed by aspiration/reflux/choking (19.8%), other conditions (15.6%), other cardiovascular conditions (non acute myocardial nor other ischaemic heart disease: 14.5%), then other respiratory conditions. For the adults with Down syndrome, Down syndrome was the most common, then dementia, then respiratory infection, then aspiration/reflux/choking. For the adults without Down syndrome,

For peer review only - http://bmjopen.bmj.com/site/about/guidelines.xhtml

respiratory infection was the most common cause, then aspiration/reflux/choking, then other condition, then other respiratory conditions and intellectual disabilities.

- Insert table 5 about here –

Avoidable deaths

According to the ONS list of avoidable deaths, 102 (38.9%) of the 262 deaths were avoidable; 78 (29.8%) were deaths that are amenable to good health care, whilst 51 (19.5%) were preventable deaths. 27 (10.3%) deaths were classed as both amenable and preventable deaths. This compares to published Scottish death data showing in 2018 that 28% of deaths were avoidable; 14% amenable and 24% preventable, similar to the figures in the previous four years (data not available prior to 2014).[30] For the 57 deaths of adults with Down syndrome, 17 (29.8%) deaths were avoidable, 15 (26.3%) deaths were amenable to good health care, whilst 7 (12.3%) were preventable. 5 (8.8%) were both amenable and preventable. For the 205 deaths of adults without Down syndrome, 85 (41.5%) were avoidable, 63 (30.7%) deaths were amenable to good health care, whilst 44 (21.5%) were preventable. 22 (10.7%) were both amenable and preventable.

Place of death

Of the 262 participants for whom place of death was known, 158 (60.3%) died in an NHS Hospital, 70 (26.7%) died at home, and 34 (13.0%) died within other hospitals/care facilities. This was similar for both the adults with Down syndrome: 31 (54.4%) in an NHS hospital, 17 (29.8%) at home, and 9 (15.8%) within other hospitals/care facilities; and the adults without Down syndrome: 127 (62.0%) in an NHS hospital, 53 (25.9%) at home, and 25 (12.2%) within other hospitals/care facilities.

For peer review only - http://bmjopen.bmj.com/site/about/guidelines.xhtml

Factors associated with risk of death

The results from the univariate cox proportional hazards models indicated that of the original 50 potential variables, factors associated with risk of death were (supplementary table 3):

- Demographics – age, more severe learning disabilities, accommodation type (not living with family carer), not having day-time occupation, and being a smoker (but not sex, the extent of neighbourhood deprivation, civil status, nor Down syndrome, in view of the confidence intervals).
- Clinical conditions – having spastic quadriplegia, hearing impairment, visual impairment, diabetes, percutaneous endoscopic gastrostomy/tube fed, constipation, ataxia/gait disorder, osteoporosis, hypertension, dysphagia, dyspnoea, gastro-oesophageal reflux disorder, lower respiratory tract infection, total number of physical health disorders, not having impaired mobility, not having urinary incontinence, not having bowel incontinence, and not having autism (but not epilepsy, body mass index, nail disorder, epidermal thickening, cerebral palsy, fungal infection, musculoskeletal pain, bone deformity, dental/oral problem, eczema/dermatitis, psychosis, affective disorder including bipolar affective disorder, problem behaviour, eating disorder including pica, nor any mental illness).
- Service use – number of general practitioner consultations in the previous 12 months, total number of different types of health professionals providing care at the time of the clinical assessment, (but not number of accident and emergency attendances in the previous 12 months).
- Prescriptions – antiepileptic drugs, total number of different types of drugs, (but not antipsychotic drugs, antidepressant drugs, nor anxiolytic drugs).

Table 6 shows the final model of the variables retained in the multivariable analysis for time to death. The significant factors indicating an increased risk of death were increased age, smoking, Down syndrome, diabetes, being percutaneous endoscopic gastrostomy/tube fed, lower respiratory tract infection at cohort inception, epilepsy,

For peer review only - http://bmjopen.bmj.com/site/about/guidelines.xhtml

hearing impairment, and total number of different types of drugs prescribed, whilst bowel incontinence showed a reduced risk of death. Of note, level of intellectual disabilities whilst significant in the univariate analysis, was not retained in the multivariable model.

- Insert table 6 about here –

Discussion

Principle findings and interpretation

As far as we are aware, this is the first population-based study of adults with intellectual disabilities to report in detail the factors associated with time to death, and to describe their causes of death and quantify the SMR separately for adults with Down syndrome and adults without Down syndrome. This is important, since adults with Down syndrome form a notable proportion of all adults with intellectual disabilities (19% in this cohort), and because they have a different pattern of clinical conditions compared with other adults with intellectual disabilities.[20] We found that aspiration/reflux/choking is the most common underlying cause of death in adults with intellectual disabilities, followed by respiratory infection. They are also the most common all contributing causes of death. The profile differed in the adults with Down syndrome for whom "Down syndrome", followed by dementia, were recorded as the most common underlying cause of death, and all contributing causes of death; with the next most common all contributing cause of death being respiratory infection, then aspiration/reflux/choking. The proportion of deaths that would have been amenable to good care for adults with intellectual disabilities was more than double that seen in the general population. Although aspiration/reflux/choking is not included in the ONS list of avoidable deaths, and therefore not included in the figures we report on amenable deaths, we consider that good care could have prevented many of these deaths. This appears to be very important for adults with intellectual disabilities irrespective of whether they have Down

For peer review only - http://bmjopen.bmj.com/site/about/guidelines.xhtml

syndrome. Similarly, some other causes of deaths within this cohort, such as constipation/mega-colon, and urinary tract infections do not appear on the ONS list of avoidable deaths.

Clearly, this pattern of causes of death differs from that seen in the general population, in whom the most common underlying causes of death are heart disease, then dementia, then lung cancer in men, and dementia, then heart disease, then stroke in women.[31] When all cancers are grouped together, in the general population, cancer is the leading underlying cause of death in 30% of men and 26% of women, compared with this study reporting 0% for adults with Down syndrome, and 15.2% for adults with intellectual disabilities without Down syndrome – presumably as the adults with intellectual disabilities are dying younger from other causes, and cancers increase with age.

We found an overall SMR of 2.24; 5.28 in the adults with Down syndrome and 1.93 for the adults without Down syndrome. SMRs were higher for most ICD-10 chapter groupings of conditions. It was higher in the women than the men, as has been previously reported in most (supplementary table 1), but not all[10,18] previous reports. The reason for this is unknown; in the general population, mortality rates have fallen in recent decades, and more so in middle and older aged men than women (i.e. the sex gap is narrowing at these ages), but we do not know what trends over time there have been for people with intellectual disabilities. Having intellectual disabilities removes differences in lifespan by sex compared with the general population; but sex was not a predictor of mortality in our study, so the SMR difference may only be because of the difference found in the general population by sex. SMRs were lowest with older age groups, likely to be due to increased illness in the older general population and conversely a healthier group with intellectual disabilities living to older ages compared with those who die younger. Although SMR was higher with increasing severity of intellectual disabilities, ability level was not retained within the multivariable model on time to death. The factors that were independently associated with increased risk of

For peer review only - http://bmjopen.bmj.com/site/about/guidelines.xhtml

death, in order, were being percutaneous endoscopic gastrostomy/tube fed, Down syndrome, diabetes, having a lower respiratory tract infection at entry to the cohort, smoking, epilepsy, hearing impairment, total number of prescribed drugs, and age, whilst bowel incontinence had a reduced risk of death. Some of these predictors are similar to those reported in the general population, suggesting earlier mortality of adults with intellectual disabilities is largely accounted for by the higher rates of multi-morbidities that they experience compared with other people, and amenable deaths.[32]

Whilst accommodation type (not living with a family carer), ability level, not having day-time occupation, having spastic quadriplegia, visual impairment, constipation, ataxia/gait disorder, osteoporosis, hypertension, dysphagia, dyspnoea, gastro-oesophageal reflux disorder, total number of physical health disorders, not having impaired mobility, not having urinary incontinence, and not having autism, number of general practitioner consultations in the previous 12 months, total number of different types of health professionals providing care at the time of the health assessment, and antiepileptic drugs were related to time of death on univariate analyses, they were not retained in the multivariable model.

The majority of the adults with intellectual disabilities, with and without Down syndrome, died in an NHS hospital.

Comparison with previous literature

The overall SMR we report, higher SMR in women than men, and higher SMR at younger age groups is similar to the majority of previous reports. Most mortality studies with people with Down syndrome have been conducted with children. Previous reports of children and adults (combined) gave an SMR=5.5,[19] and for adults SMR=7.6,[9] compared with our finding for adults with Down syndrome of SMR=5.28. Recent systematic reviews reported people with intellectual disabilities on average died 20 years younger than other people, and people with Down syndrome died 28 years younger, although the majority

For peer review only - http://bmjopen.bmj.com/site/about/guidelines.xhtml

of the Down syndrome studies were not recent.[1,2] In our study we found the gap between the age at death of people with intellectual disabilities with and without Down syndrome to be only 5.3 years, possibly reflecting the increasing lifespan of people with Down syndrome exceeding increases in lifespan for people with intellectual disabilities without Down syndrome. Notably, after "Down syndrome", dementia was the most commonly reported underlying, and all contributing cause of death for the adults with Down syndrome, whereas studies in the past commented on congenital heart disease and respiratory causes.

For the cohort overall, respiratory infection and aspiration/reflux/choking were the most common all contributing causes of death. These conditions feature in previous studies on causes of death[5,6,8,10,11], although there are inconsistencies between studies. By ICD-10 chapter, our study found the most common underlying causes of death were diseases of the respiratory system, then of the circulatory system, followed by neoplasms. Others reported the most common to be vascular,[10] circulatory,[5] heart disease, [17] and jointly circulatory and neoplasm.[18]

Studies that investigated avoidable deaths in adults with intellectual disabilities found them to be more common than in the general population, due to deaths that would have been amenable to good care. Avoidable deaths have been reported in 44.7% of deaths of people with intellectual disabilities in England (mostly amenable deaths – figure not reported),[6] and in 31% in Australia,[18] compared with our figure of 38.9%. Avoidable deaths that would have been amenable to good care have been reported to occur in 37% of deaths of people with intellectual disabilities in England.[5] Our figure is slightly lower at 29.8% but still more than double that found in the Scottish general population.[30] It should be noted that the ONS list of avoidable deaths was not designed specifically for people with intellectual disabilities, and it may emphasise some causes less relevant, and omit others that might be highly relevant in this population.[5]

For peer review only - http://bmjopen.bmj.com/site/about/guidelines.xhtml

Strengths and limitations

The strengths of the study include the thorough methods of case ascertainment for intellectual disabilities at baseline with verification of intellectual disabilities and its severity, suggesting results are generalisable in other high income countries. Additionally, there were detailed clinical assessments at baseline, and a longitudinal design. The size of the cohort and the duration of follow-up is also a strength, as is the successful record linkage for 94% of participants. Our study does have a couple of limitations, specifically that the study was only conducted in one region of Scotland, and the reliance upon death certificate data to obtain cause of death.

Implications

It is important to know the factors that are associated with risk of death, and the common causes of death in this population, as these then inform the actions needed to reduce the unacceptably high SMRs experienced by people with intellectual disabilities. It is not adequate to solely rely on the public health interventions available to everyone, even when they are accessible. Aspiration, reflux, and choking could, and should, be avoided by raising awareness of its consequences (death), and putting in place training on simple measures related to feeding, positioning, food consistency, and when to seek health advice from speech and language therapy, physiotherapy, nursing, and medical advice. Carers need to be aware of how the adults they care for express pain, so that conditions such as gastrointestinal ulcers are attended to, prior to the extreme point of perforation, and so treatable conditions such as constipation and urinary tract infections are managed before they lead to respiratory distress and sepsis. Quality of care is important; adults with intellectual disabilities need just as good care for their diabetes and epilepsy (and other conditions) as the rest of the population, with reasonable adjustments to address accessibility, and accessible smoking cessation programs.

For peer review only - http://bmjopen.bmj.com/site/about/guidelines.xhtml

Future research

Further research on larger samples is needed, particularly with regards to replicating and extending our findings on the factors that are associated with risk of death, and any sex differences in them, so that practitioners can focus on actions to improve the life expectancy of adults with intellectual disabilities, with and without Down syndrome.

References

1. O'Leary L, Hughes-McCormack L, Cooper S-A. Early death and causes of death of people with intellectual disabilities: A systematic review. *J Appl Res Intellect Disabil* 2017;31:325-42.
2. O'Leary L, Hughes-McCormack L, Cooper S-A. Life expectancy and causes of death of people with Down syndrome: a systematic review. *J Appl Res Intellect Disabil* 2018;31:687-708.
3. Cooper S-A, Hughes-McCormack L, Greenlaw N, *et al.* Management and prevalence of long-term conditions in primary health care for adults with intellectual disabilities compared with the general population: a population-based cohort study. *J Appl Res Intellect Disabil* 2017;31(S1):68–81. 10.1111/jar.12386
4. Heslop P, Blair PS, Fleming P, *et al.* The Confidential Inquiry into premature deaths of people with intellectual disabilities in the UK: a population-based study. *Lancet* 2014;383:889–95.
5. Hosking FJ, Carey IM, Shah SM, *et al.* Mortality among adults with intellectual disability in England: Comparisons with the general population. *Am J Public Healt,* 2016;106:1483–90. ERRATUM: *Am J Public Health* 2016;106:e11–e12. doi: 10.2105/AJPH.2016.303240e
6. Glover G, Williams R, Heslop P, *et al.* Mortality in people with intellectual disabilities in England. *J Intellect Disabil Res* 2017;61:62-74.
7. Fosgren L, Edvinsson SO, Nystrom L, *et al.* Influence of epilepsy on mortality in mental retardation: an epidemiological study. *Epilepsia 1996;*37:956-63.

For peer review only - http://bmjopen.bmj.com/site/about/guidelines.xhtml

8. Durvasula S, Beange H, Baker W. Mortality of people with intellectual disability in northern Sydney. *J Intellect Development Disabil* 2002; 27:255-64.
9. Tyrer F, Smith LK, McGrother CW. Mortality in adults with moderate to profound intellectual disability: a population-based study. *J Intellectl Disabil Res* 2007;51:520–7.
10. Patja K, Mölsä P, Iivanainen M. Cause-specific mortality of people with intellectual disability in a population-based, 35-year follow-up study. *J Intellect Disabil Res* 201;45:30–40.
11. Tyrer F, McGrother C. Cause-specific mortality and death certificate reporting in adults with moderate to profound intellectual disability. *J Intellect Disabil Res* 2009;53:898–904.
12. Ouellette-Kuntz H, Shooshtari S, Balogh R, *et al.* Understanding information about mortality among people with intellectual and developmental disabilities in Canada. *J Appl Res Intellect Disabil* 2015;28:423–35.
13. Florio T, Trollor J. Mortality among a cohort of persons with an intellectual disability in New South Wales, Australia. *J Appl Res Intellect Disabil* 2015;28:383–93.
14. McCarron M, Carroll R, Kelly C, *et al.* Mortality rates in the general Irish population compared to those with an intellectual disability from 2003 to 2012. *J Appl Res Intellect Disabil* 2015;28:406–13.
15. Heslop P, Glover G. Mortality of people with intellectual disabilties in England: a comparison of data from existing sources. *J Appl Res Intellect Disabil* 2015;28:414-22.
16. Arvio M, Salokivi T, Tiitinen A, *et al.* Mortality in individuals with intellectual disabilities in Finland. *Brain Behavior* 2016;6:e00431.
17. Lauer E. 2012 & 2013 Mortality Report, Massachusetts. 2016; https://shriver.umassmed.edu/sites/shriver.umassmed.edu/files/2012-13%20DDS%20Mortality%20Report%20Final_v2.pdf [accessed 9.7.19].

For peer review only - http://bmjopen.bmj.com/site/about/guidelines.xhtml

18. Troller J, Srasuebkul P, Xu H, *et al.* Cause of death and potentially avoidable deaths in Australian adults with intellectual disability using retrospective linked data. *BMJOpen* 2017;7:e013489
19. Day SM, Strauss DJ, Shavelle RM, Reynolds RJ. Mortality and causes of death in persons with Down syndrome in California. *Developmental Med Child Neurology* 2005;47:171-6.
20. Kinnear D, Morrison J, Allan L, *et al.* The prevalence of multi-morbidity in a cohort of adults with intellectual disabilities, with and without Down syndrome. *BMJOpen* 2018; **0**:e018292
21. World Health Organisation. *International Statistical Classification of Diseases and Related Health Problems, 10th Revision.* Geneva: World Health Organisation, 1992.
22. World Health Organisation. *The ICD-10 Classification of Mental and Behavioural Disorders: Diagnostic Criteria for Research.* Geneva: World Health organisation, 1993.
23. American Psychiatric Association. *Diagnostic and Statistical Manual of Mental Disorders, Fourth Edition Revised.* Washington, DC: American Psychiatric Association, 2000.
24. Royal College of Psychiatrists. *DC-LD [Diagnostic Criteria for Psychiatric Disorders for Use with Adults with Learning Disabilities / Mental Retardation],* London: Gaskell Press, 2001.
25. Cooper S-A, Smiley E, Morrison J, *et al.* Prevalence of and associations with mental ill-health in adults with intellectual disabilities. *Br J Psychiatry 2007;*190:27-35**.**
26. Finlayson J, Jackson A, Cooper S-A, *et al.* Understanding predictors of low physical exercise in adults with intellectual disabilities. *J Appl Res Intellect Disabil* 2009;22:236-47.
27. National Records of Scotland Vital Events Reference Tables 2010. Section 5: Deaths https://www.nrscotland.gov.uk/statistics-and-data/statistics/statistics-by-theme/vital-events/general-publications/vital-events-reference-tables/archive/2010/section-5-deaths

For peer review only - http://bmjopen.bmj.com/site/about/guidelines.xhtml

28. National Records of Scotland. Mid-2010 Population Estimates Scotland Population estimates by sex, age and administrative area. https://www.nrscotland.gov.uk/files/statistics/population-estimates/mid-2010/mid-year-pop-est-2010.pdf

29. Office for National Statistics Avoidable Mortality in England and Wales, 2013, Newport, South Wales. 2015. https://www.ons.gov.uk/peoplepopulationandcommunity/healthandsocialcare/causesofdeath/bulletins/avoidablemortalityinenglandandwales/2015

30. National Records of Scotland. Avoidable mortality. https://www.nrscotland.gov.uk/statistics-and-data/statistics/statistics-by-theme/vital-events/deaths/avoidable-mortality

31. Public Health England. Health profile for England. 2018 https://www.gov.uk/government/publications/health-profile-for-england-2018/chapter-2-trends-in-mortality

32. Cooper S-A, McLean G, Guthrie B, *et al.* Multiple physical and mental health comorbidity in adults with intellectual disabilities: population-based cross-sectional analysis. *BMC Family Practice*, 2015;DOI:10.1186/s12875-015-0329-3.

Funding

This work was supported by the UK Medical Research Council, grant number: MC_PC_1717), and the Scottish Government via the Scottish Learning Disabilities Observatory.

The study sponsor and funders had no role in the study design; in the collection, analysis, and interpretation of data; in the writing of the report; and in the decision to submit the article for publication.

The researchers are independent from the funders.

For peer review only - http://bmjopen.bmj.com/site/about/guidelines.xhtml

Competing interests

The authors declare no competing interests.

Author's contributions

S-AC is principle investigator, she conceived and managed the project, interpreted data, and wrote the first draft of the manuscript. LA contributed to the conception of the project, and project management. NG designed and supervised the statistical analysis, and contributed to data interpretation and drafting of the manuscript. PMcS implemented and refined the statistical analysis, and contributed to data interpretation, and drafting of the manuscript. AJ implemented and refined the statistical analysis, and contributed to data interpretation. AH contributed to data linkage and interpretation, and drafting of the manuscript. CMcC provided expertise on data linkage and methods, and drafting of the manuscript. DK contributed to data interpretation and drafting of the manuscript. CM contributed to data interpretation, and drafting of the manuscript. All approved the final version of the manuscript. S-AC is the study guarantor.

Data sharing

Data is available via NHS GG&C safe haven upon application.

Acknowledgments

We thank the NHS Greater Glasgow and Clyde learning disabilities primary care liaison team for their contribution to the study, and the participants with intellectual disabilities and their carers.

For peer review only - http://bmjopen.bmj.com/site/about/guidelines.xhtml

Table 1. Cohort characteristics at time of the health assessment, summarised overall and by death status during the follow-up period

Variable	Statistics / Groups	All participants (N=961)	Deceased participants (N=294)	Alive participants (N=667)
Age (years)	Mean (SD)	44.1 (14.6)	52.4 (13.6)	40.5 (13.6)
	Min, max	16, 83	18, 83	16, 77
Age group	16-25 years	127 (13.2%)	10 (3.4%)	117 (17.5%)
	26-35 years	153 (15.9%)	26 (8.8%)	127 (19.0%)
	36-45 years	246 (25.6%)	49 (16.7%)	197 (29.5)
	46-55 years	205 (21.3%)	85 (28.8%)	120 (18.0%)
	>55 years	230 (23.9%)	124 (42.0%)	106 (15.9%)
Sex	Male	525 (54.6%)	154 (52.4%)	371 (55.6%)
	Female	436 (45.3%)	140 (47.5%)	296 (44.4%)
Ability level	Mild ID	382 (39.7%)	92 (31.2%)	290 (43.5%)
	Moderate ID	236 (24.5%)	73 (24.7%)	163 (24.4%)
	Severe ID	180 (18.7%)	67 (22.7%)	113 (16.9%)
	Profound ID	163 (17.0%)	62 (21.1%)	101 (15.1%)
Accommodation type	Family carer	374 (38.9%)	70 (23.8%)	304 (45.6%)
	Independent	93 (9.7%)	36 (12.2%)	57 (8.5%)
	Paid support	435 (45.2%)	161 (54.6%)	274 (41.1%)
	Congregate care	59 (6.1%)	27 (9.2%)	32 (4.8%)
Down syndrome	No	782 (81.4%)	230 (78.2%)	552 (82.8%)
	Yes	179 (18.6%)	64 (21.7%)	115 (17.2%)

ID=intellectual disabilities; SD=standard deviation

For peer review only

For peer review only - http://bmjopen.bmj.com/site/about/guidelines.xhtml

Table 2. Standardised mortality ratios

Variable	Groups	SMR (95% CI)
All participants	-	2.24 (1.99, 2.50)
Age group*	15-25 years	18.73 (0.37, 37.09)
	26-35 years	4.21 (1.29, 7.13)
	36-45 years	3.86 (2.28, 5.44)
	46-55 years	3.77 (2.90, 4.74)
	>55 years	1.86 (1.60, 2.12)
Sex	Male	1.69 (1.42, 1.95)
	Female	3.48 (2.90, 4.06)
Ability level	Mild ID	1.60 (1.27, 1.92)
	Moderate ID	2.10 (1.62, 2.58)
	Severe ID	2.78 (2.11, 3.44)
	Profound ID	4.14 (3.11, 5.17)
Down syndrome	No	1.93 (1.68, 2.18)
	Yes	5.28 (3.98, 6.57)
Underlying causes of death grouped by ICD-10 chapter**	Congenital malformations, deformations and chromosomal abnormalities	17.26 (10.75, 23.78)
	Diseases of the blood and blood-forming organs and certain disorders involving the immune mechanism	7.50 (-7.20, 22.20)
	Diseases of the circulatory system	5.55 (4.01, 7.09)
	Diseases of the digestive system	16.13 (8.23, 24.04)
	Diseases of the genitourinary system	3.65 (0.73, 6.57)
	Diseases of the musculoskeletal system and connective tissue	5.40 (-0.71, 11.52)
	Diseases of the nervous system	7.73 (5.13, 10.32)
	Diseases of the respiratory system	6.78 (5.02, 8.54)

For peer review only - http://bmjopen.bmj.com/site/about/guidelines.xhtml

	Diseases of the skin and subcutaneous tissue	2.75 (-2.64, 8.15)
	Endocrine, nutritional and metabolic diseases	3.43 (1.05, 5.81)
	External causes of morbidity and mobility	11.08 (3.40, 18.76)
	Infectious and parasitic diseases	8.93 (1.78, 16.07)
	Mental and behavioural disorders	12.64 (3.27, 22.00)
	Neoplasms	6.31 (4.19, 8.43)
	Symptoms, signs and abnormal clinical and laboratory findings, not elsewhere classified	19.51 (0.39, 38.63)

CI=confidence intervals; ID=intellectual disabilities; SMR=standardised mortality ratios

*Data used for comparison with General Population (GG&C Health Board) provides data in 5 year age bands therefore 15+. Data on adults with ID are 16+

** Negative Lower CI and wide CIs indicate low number of observed deaths in study population

For peer review only

For peer review only - http://bmjopen.bmj.com/site/about/guidelines.xhtml

Table 3. Underlying causes of death grouped by ICD-10 chapter, where cause of death is known

ICD-10 chapter	Participants with Down syndrome (N=57)	Participants without Down syndrome (N=205)
Certain infectious and parasitic diseases	5 (8.8%)	<5
Neoplasms	<5	33 (16.1%)
Diseases of the blood and blood-forming organs and certain disorders involving the immune mechanism	0	<5
Endocrine, nutritional and metabolic diseases	0	8 (3.9%)
Mental and behavioural disorders	5 (8.8%)	<5
Diseases of the nervous system	7 (12.3%)	27 (13.2%)
Diseases of the eye and adnexa	0	0
Diseases of the ear and mastoid process	0	0
Diseases of the circulatory system	8 (14.0%)	42 (20.5%)
Diseases of the respiratory system	8 (14.0%)	49 (23.9%)
Diseases of the digestive system	0	16 (7.8%)
Diseases of the skin and subcutaneous tissue	0	<5
Diseases of the musculoskeletal system and connective tissue	0	<5
Diseases of the genitourinary system	<5	5 (2.4%)
Pregnancy, childbirth and the puerperium	0	0
Certain conditions originating in the perinatal period	0	0
Congenital malformations, deformations and chromosomal abnormalities	21 (36.8%)	6 (2.9%)
Symptoms, signs and abnormal clinical and laboratory findings, not elsewhere classified	0	<5
External causes of morbidity and mortality	<5	7 (3.4%)
All deaths	57 (100%)	205 (100%)

ICD-10=International Statistical Classification of Diseases and Related Health Problems, 10th Revision

For peer review only - http://bmjopen.bmj.com/site/about/guidelines.xhtml

Table 4. Underlying causes of death grouped by specific individual causes or related groups of causes, where cause of death is known

Causes	Participants with Down syndrome (N=57)	Participants without Down syndrome (N=205)
Aspiration/reflux/choking	<5	22 (10.8%)
Respiratory infection	<5	21 (10.3%)
Down syndrome	21 (36.8%)	0
Other malignancy	0	19 (9.3%)
Other condition	<5	17 (8.3%)
Epilepsies	<5	13 (6.4%)
Acute myocardial infarction	<5	13 (6.4%)
Gastro-intestinal malignancy	<5	12 (5.9%)
Stroke	<5	11 (5.4%)
Other cardiovascular disease	<5	11 (5.4%)
Other respiratory condition	<5	9 (4.4%)
Other infection	5 (8.8%)	6 (2.9%)
Cerebral palsy	0	11 (5.4%)
Dementia	9 (15.8%)	0
Other gastrointestinal disorders	0	8 (3.9%)
Ulcer/gastrointestinal perforation	0	7 (3.4%)
Diabetes	0	7 (3.4%)
Other congenital condition	0	6 (2.9%)
Other ischaemic heart condition	0	6 (2.9)
Mental health	0	<5
Other neurological conditions	<5	<5
Renal failure	<5	<5
All deaths	57 (100%)	205 (100%)

For peer review only - http://bmjopen.bmj.com/site/about/guidelines.xhtml

Table 5. All contributing causes of death grouped by specific individual causes or related groups of causes, where cause of death is known

Causes	Participants with Down syndrome (N=57)	Participants without Down syndrome (N=205)
Respiratory infection	22 (38.6%)	49 (23.9%)
Aspiration/reflux/choking	11 (19.3%)	41 (20.0%)
Down syndrome	43 (75.4%)	<5
Other condition	8 (14.0%)	33 (16.1%)
Other cardiovascular disease	8 (14.0%)	30 (14.6%)
Other respiratory conditions	<5	31 (15.1%)
Other infection	9 (15.8%)	24 (11.7%)
Intellectual disabilities	<5	31 (15.1%)
Epilepsies	8 (14.0%)	24 (11.7%)
Dementia	24 (42.1%)	<5
Other neoplasms	<5	23 (11.2%)
Cerebral palsy	<5	24 (11.7%)
Acute myocardial infarction	5 (8.8%)	19 (9.3%)
Other gastrointestinal disorders	<5	18 (8.8%)
Diabetes	<5	19 (9.3%)
Other ischaemic heart disease	<5	19 (9.3%)
Renal failure	<5	16 (7.8%)
Stroke	<5	17 (8.3%)
Other congenital condition	<5	15 (7.3%)
Gastrointestinal malignant neoplasm	<5	12 (5.9%)
Ulcer/gastrointestinal perforation	<5	10 (4.9%)
Mental health	<5	10 (4.9%)
Other neurological condition	<5	8 (3.9%)
Heart failure	<5	7 (3.4%)
Injuries and accidents	<5	8 (3.9%)
Medical/surgical complications	<5	<5
Secondary malignancies	<5	<5
Thyroid disorders	<5	<5
Metabolic disorder	<5	<5
All deaths	57 (100%)	205 (100%)

For peer review only - http://bmjopen.bmj.com/site/about/guidelines.xhtml

Table 6. Multivariable model results for the outcome time to death

Variable		Hazard ratio	95% CI	p-value
Age		1.056	1.046, 1.066	<0.0001
Smoker	No	1	-	
	Yes	1.531	1.1011, 2.128	0.0112
Down syndrome	No	1	-	
	Yes	2.440	1.787, 3.332	<0.0001
Epilepsy	No	1	-	
	Yes	1.511	1.173, 1.946	0.0014
Hearing impairment	No	1	-	
	Yes	1.320	1.030, 1.692	0.0284
Bowel incontinence	No	1	-	
	Yes	0.490	0.376, 0.640	<0.0001
Diabetes	No	1	-	
	Yes	2.346	1.553, 3.542	<0.0001
PEG/tube fed	No	1	-	
	Yes	2.346	1.135, 5.989	0.00240
Lower respiratory track infection	No	1	-	
	Yes	1.782	1.315, 2.415	0.0002
Total number of prescribed drugs		1.066	1.016, 1.118	0.0085

CI=confidence interval; PEG=percutaneous endoscopic gastrostomy

For peer review only

Supplementary table 1. Previously reported standardised mortality ratios, causes, and risks for death

Author	Country	SMR (95% confidence interval)	Number of deaths	Causes of death and risk factors for death
Forsgren et al (1996)[7]	Sweden	4.2 (3.3, 5.3) at 20-59y; 1.1 (0.9, 1.5) at 60+y<br>*Without epilepsy:*<br>3.8 (2.8, 5.0) at 20-59y; 1.1 (0.8, 1.5) at 60+y<br>*With epilepsy:*<br>5.0 (2.9, 8.7) at 20-59y; 2.4 (0.9, 6.1) at 60+y<br>*With epilepsy and cerebral palsy:*<br>8.0 (4.1, 15.7) at 20-59y; 0.9 (0.1, 6.6) at 60+y<br>*M:* 1.6 (1.2, 2.0) at 0-60+y<br>*F:* 2.6 (2.0, 3.3) at 0-60+y<br>*Mild ID:* 1.8 (1.1, 2.7) at 0-60+y<br>*Moderate ID:* 1.5 (1.1, 2.0) at 0-60+y<br>*Severe ID:* 2.0 (1.5, 2.6) at 0-60+y<br>*Profound ID:* 8.1 (5.6, 11.7) at 0-60+y	124 at 0-60+y; 112 at 20-60+y	*Underlying cause at 0-60+y:*<br>Congenital anomalies: SMR=46.3 (32.9, 65.0)<br>Nervous system: SMR=9.7 (5.5, 17.0)<br>Mental disorder: SMR=4.0 (1.9, 8.4)<br>Respiratory: SMR=3.3 (2.0, 5.5)<br>Circulatory: SMR=2.1 (1.6, 2.7)<br>Violent death: SMR=1.4 (0.6, 2.8)<br>Neoplasm: SMR=0.9 (0.6, 1.6)
Durvasula & Beange (2002)[8]	Australia	4.9 (3.4, 6.4) at 10-59y<br>*M:* 4.1 (2.4, 5.9) at 10-59y<br>*F:* 6.2 (3.3, 9.1) at 10-59y	40 at 10-59y; 31 at 20-59y	*Underlying cause at 10-59y:*<br>Respiratory: 35% (pneumonia, aspiration)<br>External causes: 20%<br>Neoplasm: 17.5%<br>Heart disease: 15% (congenital heart disease 50%)<br>Gastrointestinal: 7.5% (ischaemic bowel, perforated peptic ulcer, post-operative peritonitis)<br>Seizure: 5%
Tyrer et al (2007)[9]	England	3.24 (2.93, 3.56) at 20-70+y<br>*M:* 2.86 (2.50, 3.26) at 20-70+y<br>*F:* 3.63 (3.12, 4.20) at 20-70+y<br>1.51 (1.23, 1.83) to 11.50 (8.14, 15.78) at 20-70+y<br>*M:* 1.39 (1.03, 1.82) to 8.83 (5.60, 13.25) at 20-70+y<br>*F:* 1.60 (1.18, 2.12) to 17.22 (9.64, 28.4) at 20-70+y<br>*With Down syndrome:* 7.60 at 20-70+y<br>*Without Down syndrome:* 2.70 at 20-70+y	409 at 20-70+y	Not reported
Patja et al (2008)[10]	Finland	*M:* 2.2 at 20-39y, 1.0 at 40-59y, 1.0 at 60+y<br>*F:* 1.4 at 20-39y, 1.1 at 40-49y, 1.0 at 60+y<br>*Mild ID:*<br>M: 1.6 at 20-39y, 1.0 at 40-59y, 1.0 at 60+y<br>F: 1.2 at 20-39y, 1.1 at 40-49y, 1.0 at 60+y	1,046 at 20-97y	*Underlying cause at 2-97y:*<br>Vascular: 36% (cardiac infarct 33%, cerebral infarct 33%, congenital heart disease 18%, pulmonary infarct 6%)<br>Respiratory: 22% (pneumonia 83%, COPD 11%)

For peer review only

Rates, causes, place, and predictors of mortality in adults with intellectual disabilities with and without Down syndrome: cohort study with record linkage

		Moderate ID:<br>M: 2.3 at 20-39y, 1.1 at 40-59y, 1.0 at 60+y<br>F: 1.5 at 20-39y, 1.1 at 40-49y, 1.0 at 60+y<br>*Severe ID:*<br>M: 2.6 at 20-39y, 1.2 at 40-59y, 1.0 at 60+y<br>F: 1.6 at 20-39y, 1.0 at 40-49y, 1.0 at 60+y<br>*Profound ID:*<br>M: 2.1 at 20-39y, 1.1 at 40-59y, 1.0 at 60+y<br>F: 1.3 at 20-39y, 1.2 at 40-49y, 1.0 at 60+y		Neoplasm: 11% (digestive 44%, respiratory 15%, urogenital, 12%)<br>Digestive: 7% (intestinal obstruction 25%, ulcer perforation 13%)<br>Accidents and poisonings: 7% (commonest was fatal fracture, then drowning)<br>Vascular, neoplasm, and accident causes were less common than sex-age-matched general population; Digestive were 2.5 times, Respiratory 2.6-5.8 times more common
Tyrer & McGother (2009)[11]	England	2.77 (2.53, 3.03) at 20+y<br>*M:* 2.28 (2.02, 2.56) at 20+y<br>*F:* 3.24 (2.83, 3.69) at 20+y	503 at 20+y	*Underlying cause at 20+y:*<br>Pneumonia: 13.1%, SMR=6.47 (5.00, 8.23)<br>Nervous system: 13.1%, SMR=16.30 (12.61, 20.74)<br>Other respiratory: 12.9%, SMR=4.64 (3.58, 5.91)<br>Ischaemic heart disease: 11.5%, SMR=1.49 (1.13, 1.92)<br>Neoplasm: 9.3%<br>Congenital anomalies: 9.1%, SMR=85.60 (62.67, 114.18)<br>Cerebrovascular disease: 7.8%, SMR=2.40 (1.71. 3.28)
Oullette-Kuntz et al (2015)[12]	Canada	2.5 (2.1, 2.9) at 0-60+y<br>*M:* 2.1 (1.7, 2.6) at 0-60+y<br>*F:* 3.0 (2.4, 3.8) at 0-60+y<br>*M:* 1.7 (1.3, 2.3) to 3.4 (2.3, 4.7) at 20-60+y<br>*F:* 2.1 (1.4, 2.9) to 6.1 (4.1, 8.6) at 20-60+y	172 at 0-60+y; 158 at 20-60+y	*Risk factors for death:*<br>Age, Down syndrome (OR=1.76 at 20-39y; OR=1.69 at 40-59y: OR=22.34 at 60+y), cerebral palsy (OR=2.39 at 20-39y; OR=0.93 at 40-59y: OR=0.50 at 60+y), blindness/low vision (OR not given), technological dependance/medical fragility (OR=11.96 at 20-39y; OR=7.28 at 40-59y: OR=3.42 at 60+y), wheelchair dependence (OR=5.96 at 20-39y; OR=2.89 at 40-59y: OR=2.56 at 60+y), mobility impairment without wheelchair dependence (OR not given), epilepsy (OR=1.83 at 20-39y; OR=1.80 at 40-59y: OR=1.09 at 60+y)
Florio & Troller (2015)[13]	Australia	2.48 (2.32, 2.64) at 0-85+y<br>3.15 (2.94, 3.38) at 5-69y<br>*M:* 2.52 (2.29, 2.77) at 5-69y<br>*F:* 4.26 (3.83, 4.74) at 5-69y	953 at 0-85+y; 831 at 15+y	Not reported
McCarron et al (2015)[14]	Republic of Ireland	3.85 (3.70, 4.00) at 0-80+y<br>*M:* 3.09 (2.93, 3.25) at 0-80+y<br>*F:* 4.90 (4.63, 5.17) at 0-80+y<br>2.71 (2.41, 3.04) to 6.09 (5.29, 6.96) at 20-80y<br>*M:* 2.50 (2.18, 2.86) to 4.50 (3.69, 5.44) at 20-80y<br>*F:* 2.71 (2.32, 3.14) to 10.07 (8.99, 13.10) at 20-80y	2,666 at 0-80+y; 2,394 at 20-80+y	Not reported

For peer review only

Rates, causes, place, and predictors of mortality in adults with intellectual disabilities with and without Down syndrome: cohort study with record linkage

Heslop & Glover (2015)[15]	England	Median 2.13 (interquartile range 1.09, 2.83) across geographic areas at 18-65+y	18-65+y	Not reported
Arvio et al (2016)[16]	Finland	*Mild ID:*<br>2.28 (2.18, 2.39) at 0-60+y<br>1.99 (1.85, 2.13) to 2.77 (2.36, 3.23) at 15-60+y<br>*M:* 2.01 (1.88, 2.14) at 0-60+y<br>*F:* 2.80 (2.60, 3.01) at 0-60+y<br>*Severe ID:*<br>3.41 (3.30, 3.52) at 0-60+y<br>2.07 (1.96, 2.19) to 8.77 (7.77, 9.87) at 15-60+y<br>*M:* 2.59 (2.48, 2.72) at 0-60+y<br>*F:* 5.24 (4.99, 5.50) at 0-60+y	5,171 at 0-60+y; 5,053 at 15-60y	Not reported
Hosking et al (2016)[5]	England	HR=3.62 (3.33, 3.93) at 18-84y<br>*M:* HR=3.30 (2.96, 3.68) at 18-84y<br>*F:* HR=4.10 (3.61, 4.66) at 18-84y<br>*With Down syndrome:* HR=9.21 (7.22, 11.76)<br>*Without Down syndrome:* HR=3.19 (2.92, 3.49)<br>*With epilepsy:* HR=6.04 (5.04, 7.24)<br>*Without epilepsy:* HR=3.18 (2.90, 3.50)<br>*With high level of support needs:* HR=4.77 (4.08, 5.59)<br>*Without high level of support needs:* HR=3.28 (2.98, 3.62)<br>*With autism:* HR=2.39 (1.45, 3.96)<br>*Without autism:* HR=3.66 (3.37, 3.98)<br>*In communal/shared homes:* HR=4.99 (4.36, 5.73)<br>*Not in communal/shared homes:* HR=3.05 (2.74, 3.30)	656 at 18-84y	*Underlying cause at 18-84y:*<br>Circulatory: 21.6%, HR=3.05 (2.56, 3.64)<br>Respiratory: 18.8% (pneumonia and aspiration pneumonia), HR=6.68 (5.38, 8.29)<br>Neoplasm: 14.9%, HR=1.44 (1.18, 1.76)<br>Nervous system: 11.6%, HR=13.79 (9.70, 19.62)<br>Digestive: 7.0%, HR=4.02 (2.92, 5.54)<br>Congenital anomalies: 6.9%, HR could not be estimated<br>Mental disorders: 5.3%, HR=7.99 (5.19, 12.31)<br>External causes: 4.1%, HR=1.85 (1.26, 2.71)<br>Genitourinary: 3.5%, HR=10.89 (6.09, 19.47)<br>Endocrine, nutritional, and metabolic: 2.0%, HR=5.38 (2.79, 10.07)<br>*Down syndrome:* Respiratory: 20.3% (or 42.4% if "Down syndrome" is excluded as an underlying cause of death)<br>*Avoidable deaths:*<br>37% amenable (23% controls), 19% preventable (40% controls)
Lauer (2016)[17]	USA	Not reported	438 in 2012, 409 in 2013, at 18+y	*Major cause of death, 2012, 2013*<br>Heart disease: 16.0%, 13.7%<br>Neoplasm: 13.7%, 13.4%<br>Alzheimer disease: 13.0%-12.2% (48% in Down syndrome)<br>Aspiration pneumonia: 9.4%, 8.6%<br>Septicaemia: 10.0%, 8.6%<br>Chronic lower respiratory diseases: 4.6%, 6.6%<br>Unintentional injury: 4.8%, 3.2%

For peer review only

Rates, causes, place, and predictors of mortality in adults with intellectual disabilities with and without Down syndrome: cohort study with record linkage

Troller et al (2017)[18]	Australia	1.3 (1.2, 1.5) at 20+y<br>4.0 (3.1, 5.2) at 20-44y<br>2.3 (2.0, 2.7) at 45-64y<br>1.0 (0.8, 1.20 at 65+y<br>*M:*1.4 (1.1, 1.6) at 20+y<br>*F:* 1.3 (1.1, 1.6) at 20+y	732 at 20-65+y	*Underlying cause at 20-65+y:*<br>Circulatory: 18%<br>Neoplasm: 18%<br>Nervous: 16%<br>Respiratory: 12%<br>Congenital anomaly: 11%<br>Injury and poisoning: 6%<br>Digestive: 5%<br>*Avoidable deaths:* 31%
Glover et al (2017)[6]	England	3.18 (2.94, 3.43) at 0-99y<br>*M:* 3.03 (2.73, 3.35) at 0-99y<br>*F:* 3.40 (3.02, 3.81) at 0-99y<br>1.6 (1.2, 2.1) to 7.8 (5.4, 11.1) at 18-99y<br>*M:* 1.5 (0.9, 2.2) to 6.6 (4.0, 10.1) at 18-99y<br>*F:* 1.7 (1.1, 2.4) to 11.6 (6.0, 20.2) at 18-99y	664 at 0-99y	*Underlying cause at 0-99y:*<br>Circulatory: 22.9% (ischaemic heart disease 37.5%, cerebrovascular 25.7%, thrombophlebitis 6.6%, cardiomyopathy 5.9%, PE 3.9%), SMR=2.8 (2.4, 3.3)<br>Respiratory: 17.2% (pneumonia 50.0%, pneumonitis 21.0%), SMR=4.9 (4.0, 5.9)<br>Neoplasm: 3.1% (digestive 36.8%, respiratory 13.8%, female genital tract 10.3%, lymphoid and haematopoietic 10.3%), SMR=1.1 (0.9, 1.4)<br>Nervous: 12.8%, SMR=9.8 (7.8, 12.1)<br>Congenital anomalies: 8.4%, SMR=72.9 (55.1, 94.7)<br>Digestive: 7.8%, SMR=4.0 (3.0, 5.2)<br>No ICD10 chapters had fewer than expected deaths<br>Other common single causes: dementia 33/664, epilepsy 26/664, cerebral palsy 23/664<br>*Avoidable deaths:*<br>44.7% (41.0%, 48.5%), mostly amenable<br>M: 50.9% (45.9%, 56.0%); F: 36.9% (31.5%, 42.5%)

COPD=chronic obstructive pulmonary disease; HR=hazard ratio; ID=intellectual disabilities; OR=odds ratio; PE=pulmonary embolism; SMR=standardised mortality ratio; y=years

For peer review only

Rates, causes, place, and predictors of mortality in adults with intellectual disabilities with and without Down syndrome: cohort study with record linkage

Supplementary table 2. Groupings of related causes of deaths

Infectious diseases

Infection

ENTEROCOLITIS DUE TO CLOSTRIDIUM DIFFICILE
SEPSIS DUE TO STAPHYLOCOCCUS AUREUS
SEPSIS, UNSPECIFIED
BACTERIAL INFECTION, UNSPECIFIED
SUBACUTE SCLEROSING PANENCEPHALITIS
CHRONIC VIRAL HEPATITIS B WITHOUT DELTA-AGENT
PULMONARY CANDIDIASIS
NECROTISING FASCIITIS
URINARY TRACT INFECTION, SITE NOT SPECIFIED

Neoplasms

Gastrointestinal malignant neoplasms

MALIGNANT NEOPLASM OF PAROTID GLAND
MALIGNANT NEOPLASM, OESOPHAGUS, UNSPECIFIED
MALIGNANT NEOPLASM, STOMACH, UNSPECIFIED
MALIGNANT NEOPLASM, CAECUM
MALIGNANT NEOPLASM, SIGMOID COLON
MALIGNANT NEOPLASM, COLON, UNSPECIFIED
INTRAHEPATIC BILE DUCT CARCINOMA
NEOPLASM OF UNCERTAIN OR UNKNOWN BEHAVIOUR, OTHER DIGESTIVE ORGANS

Other neoplasms

MALIGNANT NEOPLASM, LOWER LOBE, BRONCHUS OR LUNG
MALIGNANT NEOPLASM, BRONCHUS OR LUNG, UNSPECIFIED
MALIGNANT NEOPLASM, BREAST, UNSPECIFIED
MALIGNANT NEOPLASM, ENDOMETRIUM
MALIGNANT NEOPLASM OF OVARY
MALIGNANT NEOPLASM, TESTIS, UNSPECIFIED
MALIGNANT NEOPLASM, BLADDER, UNSPECIFIED
MALIGNANT NEOPLASMS OF THYROID GLAND
WALDENSTROM MACROGLOBULINAEMIA
NON-HODGKIN'S LYMPHOMA, UNSPECIFIED
MALIGNANT NEOPLASM OF UNSPECIFIED SITE
NEOPLASM OF UNCERTAIN OR UNKNOWN BEHAVIOUR, TRACHEA, BRONCHUS AND LUNG
SECONDARY MALIGNANT NEOPLASM OF LUNG
SECONDARY MALIGNANT NEOPLASM OF LIVER AND INTRAHEPATIC BILE DUCT
SECONDARY MALIGNANT NEOPLASM OF BRAIN AND CEREBRAL MENINGES
SECONDARY MALIGNANT NEOPLASM OF OTHER SPECIFIED SITES

Endocrine and metabolic diseases

Diabetes

INSULIN-DEPENDENT DIABETES MELLITUS WITHOUT COMPLICATIONS
NON-INSULIN-DEPENDENT DIABETES MELLITUS WITH RENAL COMPLICATIONS
NON-INSULIN-DEPENDENT DIABETES MELLITUS W. PERIPHERAL CIRC. COMPLICATIONS
NON-INSULIN-DEPENDENT DIABETES MELLITUS WITHOUT COMPLICATIONS
UNSPECIFIED DIABETES MELLITUS WITH RENAL COMPLICATIONS

For peer review only

UNSPECIFIED DIABETES MELLITUS WITHOUT COMPLICATIONS
ABNORMAL GLUCOSE TOLERANCE TEST
HYPERGLYCAEMIA, UNSPECIFIED

Metabolic disorders

OTHER HYPERPHENYLALANINAEMIAS
DISORDERS OF PHOSPHORUS METABOLISM & PHOSPHATASES
DISORDERS OF PLASMA-PROTEIN METABOLISM, NOT ELSEWHERE CLASSIFIED

Mental disorders

Dementias

VASCULAR DEMENTIA, UNSPECIFIED
UNSPECIFIED DEMENTIA
ALZHEIMER'S DISEASE WITH LATE ONSET
ALZHEIMER'S DISEASE, UNSPECIFIED

Mental health

MENTAL AND BEHAVIOURAL DISORDERS DUE TO ACUTE INTOXICATION WITH ALCOHOL
MENTAL AND BEHAVIOURAL DISORDERS DUE TO ALCOHOL DEPENDENCE SYNDROME
MENTAL AND BEHAVIOURAL DISORDERS DUE TO USE OF TOBACCO, UNSPECIFIED
SCHIZOPHRENIA, UNSPECIFIED
BIPOLAR AFFECTIVE DISORDER, UNSPECIFIED
OTHER & UNSPEC SYMPTOMS & SIGNS INVOLVING COGNITIVE FUNCTIONS & AWARENESS
INTENTIONAL SELF-HARM BY JUMPING FROM A HIGH PLACE

Intellectual disabilities

UNSPECIFIED MENTAL RETARDATION
DEVELOPMENTAL DISORDER OF SCHOLASTIC SKILLS, UNSPECIFIED

Nervous system

Epilepsies

GENERALIZED IDIOPATHIC EPILEPSY AND EPILEPTIC SYNDROMES
EPILEPSY, UNSPECIFIED
STATUS EPILEPTICUS, UNSPECIFIED
MYOTONIC DISORDERS
OTHER AND UNSPECIFIED CONVULSIONS

Cerebral palsy

SPASTIC QUADRAPLEGIC CEREBRAL PALSY
SPASTIC HEMIPLEGIC CEREBRAL PALSY
OTHER CEREBRAL PALSY
CEREBRAL PALSY, UNSPECIFIED
TETRAPLEGIA, UNSPECIFIED

Other neurological conditions

SEQUELAE OF INFLAMMATORY DISEASES OF CENTRAL NERVOUS SYSTEM
PARKINSON'S DISEASE
MYONEURAL DISORDER, UNSPECIFIED
ENCEPHALITIS, MYELITIS AND ENCEPHALOMYELITIS, UNSPECIFIED
ANOXIC BRAIN DAMAGE, NOT ELSEWHERE CLASSIFIED
BLINDNESS, BINOCULAR
OTHER DISORDERS OF NERVOUS SYSTEM, NOT ELSEWHERE CLASSIFIED

For peer review only

Rates, causes, place, and predictors of mortality in adults with intellectual disabilities with and without Down syndrome: cohort study with record linkage

Circulatory system

Acute myocardial infarction

ACUTE MYOCARDIAL INFARCTION, UNSPECIFIED

CARDIAC ARRECT, UNSPECIFIED

Other ischaemic heart disease

HYPERTENSIVE HEART DISEASE WITHOUT (CONGESTIVE) HEART FAILURE

ACUTE ISCHAEMIC HEART DISEASE, UNSPECIFIED

ATHEROSCLEROTIC HEART DISEASE

CHRONIC ISCHAEMIC HEART DISEASE, UNSPECIFIED

ATHEROSCLEROSIS OF AORTA

GENERALIZED AND UNSPECIFIED ATHEROSCLEROSIS

Heart failure

HEART FAILURE, UNSPECIFIED

LEFT VENTRICULAR FAILURE

CONGESTIVE HEART FAILURE

Other cardiovascular disease

PULMONARY EMBOLISM WITHOUT MENTION OF ACUTE COR PULMONALE

OTHER SPECIFIED PULMONARY HEART DISEASES

PULMONARY HEART DISEASE, UNSPECIFIED

AORTIC (VALVE) STENOSIS

ATRIAL FIBRILLATION AND FLUTTER

VENTRICULAR FIBRILLATION AND FLUTTER

OTHER ILL-DEFINED HEART DISEASES

PULMONARY OEDEMA

CARDIOGENIC SHOCK

PERIPHERAL VASCULAR DISEASE, UNSPECIFIED

PHLEBITIS AND THROMBOPHLEBITIS OF OTHER DEEP VESSELS OF LOWER EXTREMITIES

EMBOLISM AND THROMBOSIS OF OTHER SPECIFIED VEINS

ACUTE AND SUBACUTE INFECTIVE ENDOCARDITIS

ACUTE ENDOCARDITIS, UNSPECIFIED

ENDOCARDITIS, VALVE UNSPECIFIED

DILATED CARDIOMYOPATHY

CARDIOMEGALY

ESSENTIAL (PRIMARY) HYPERTENSION

Stroke

INTRACEREBRAL HAEMORRHAGE, UNSPECIFIED

CEREBRAL INFARCTION DUE TO THROMBOSIS OF PRECEREBRAL ARTERIES

CEREB INFARCT DUE TO UNSPEC OCCL/STENOSIS OF PRECEREB ARTERIES

CEREBRAL INFARCTION, UNSPECIFIED

STROKE, NOT SPECIFIED AS HAEMORRHAGE OR INFARCTION

CEREBROVASCULAR DISEASE, UNSPECIFIED

SEQUELAE OF STROKE, NOT SPECIFIED AS HAEMORRHAGE OR INFARCTION

SEQUELAE OF OTHER AND UNSPECIFIED CEREBROVASCULAR DISEASES

Respiratory system

Respiratory infection

ACUTE UPPER RESPIRATORY INFECTION, UNSPECIFIED

INFLUENZA WITH PNEUMONIA, OTHER INFLUENZA VIRUS IDENTIFIED

INFLUENZA WITH OTHER RESP MANIFESTATIONS, OTHER INFLUENZA VIRUS IDENTIFIED

For peer review only

Rates, causes, place, and predictors of mortality in adults with intellectual disabilities with and without Down syndrome: cohort study with record linkage

PNEUMONIA DUE TO STREPTOCOCCUS PNEUMONIAE
BRONCHOPNEUMONIA, UNSPECIFIED
LOBAR PNEUMONIA, UNSPECIFIED
HYPOSTATIC PNEUMONIA, UNSPECIFIED
PNEUMONIA, UNSPECIFIED
UNSPECIFIED ACUTE LOWER RESPIRATORY INFECTION
CHRONIC OBSTRUCTIVE PULMONARY DISEASE WITH ACUTE LOWER RESP INFECTION

Aspiration/reflux/choking
PNEUMONITIS DUE TO FOOD AND VOMIT
GASTRO-OESOPHAGEAL REFLUX DISEASE WITHOUT OESOPHAGITIS
INHALATION AND INGESTION OF FOOD CAUSING OBSTRUCTION OF RESPIRATORY TRACT
FOREIGN BODY IN RESPIRATORY TRACT, PART UNSPECIFIED
INHALATION/INGESTION OF OTHER OBJECTS CAUSING OBSTRUCT OF RESP TRACT

Other respiratory disorders
UNSPECIFIED CHRONIC BRONCHITIS
EMPHYSEMA, UNSPECIFIED
CHRONIC OBSTRUCTIVE PULMONARY DISEASE, UNSPECIFIED
ASTHMA, UNSPECIFIED
BRONCHIECTASIS
OTHER INTERSTITIAL PULMONARY DISEASES WITH FIBROSIS
PLEURAL EFFUSION, NOT ELSEWHERE CLASSIFIED
CHRONIC RESPIRATORY FAILURE
RESPIRATORY FAILURE, UNSPECIFIED
OTHER SPECIFIED RESPIRATORY DISORDERS
DYSPNOEA
RESPIRATORY ARREST
ASPHYXIATION
UNSPECIFIED THREAT TO BREATHING

Digestive system

Ulcer/gastrointestinal perforation
OESOPHAGITIS
PERFORATION OF INTESTINE (NONTRAUMATIC
PERITONITIS, UNSPECIFIED
GASTRIC ULCER, CHRONIC OR UNSPECIFIED WITH PERFORATION
OTHER PERITONITIS
ACUTE PERITONITIS
GASTROINTESTINAL HAEMORRHAGE, UNSPECIFIED
ULCER OF INTESTINE

Other gastrointestinal disorders
BARRETTS OESOPHAGUS
DIAPHRAGMATIC HERNIA WITHOUT OBSTRUCTION OR GANGRENE
OTHER SPECIFIED NONINFECTIVE GASTROENTERITIS AND COLITIS
ACUTE VASCULAR DISORDERS OF INTESTINE
VASCULAR DISORDER OF INTESTINE, UNSPECIFIED
VOLVULUS
OTHER AND UNSPECIFIED INTESTINAL OBSTRUCTION
CONSTIPATION
MEGACOLON, NOT ELSEWHERE CLASSIFIED

For peer review only

Rates, causes, place, and predictors of mortality in adults with intellectual disabilities with and without Down syndrome: cohort study with record linkage

ACUTE AND SUBACUTE HEPATIC FAILURE
OTHER AND UNSPECIFIED CIRRHOSIS OF LIVER
AUTOIMMUNE HEPATITIS
INFLAMMATORY LIVER DISEASE, UNSPECIFIED
OTHER SPECIFIED DISEASES OF LIVER
CALCULUS OF GALLBLADDER WITH OTHER CHOLECYSTITIS
CHOLANGITIS
ACUTE PANCREATITIS, UNSPECIFIED
PSEUDOCYST OF PANCREAS
INTESTINAL MALABSORPTION, UNSPECIFIED
DYSPHAGIA

Genitourinary system

Renal failure

CHRONIC NEPHRITIC SYNDROME, UNSPECIFIED
OTHER ACUTE RENAL FAILURE
ACUTE RENAL FAILURE, UNSPECIFIED
END-STAGE RENAL DISEASE
CHRONIC KIDNEY DISEASE, STAGE 5
CHRONIC KIDNEY DISEASE, UNSPECIFIED
UNSPECIFIED KIDNEY FAILURE

Chromosomal abnormalities

Down syndrome

DOWN'S SYNDROME, UNSPECIFIED

Other congenital condition

CONGENITAL HYDROCEPHALUS, UNSPECIFIED
SPINA BIFIDA, UNSPECIFIED
CONGENITAL MALFORMATION OF HEART, UNSPECIFIED
CONGENITAL DEFORMITY OF SPINE
CONGEN MALFORMATION SYNDROMES PREDOMINANTLY ASSOCIATED WITH SHORT STATURE
MARFAN'S SYNDROME
OTHER SPECIFIED CONGEN MALFORMATION SYNDROMES, NOT ELSEWHERE CLASSIFIED
CONGENITAL MALFORMATION, UNSPECIFIED
KLINEFELTER'S SYNDROME, UNSPECIFIED
FRAGILE X CHROMOSOME
OTHER LACK OF EXPECTED NORMAL PHYSIOLOGICAL DEVELOPMENT

Other conditions occurring with small frequency

Other condition

DECUBITUS ULCER AND PRESSURE AREA
SCOLIOSIS, UNSPECIFIED
URETHRAL STRICTURE, UNSPECIFIED
EPISTAXIS
IMMOBILITY
MALAISE AND FATIGUE
GENERALIZED ENLARGED LYMPH NODES
INSUFFICIENT INTAKE OF FOOD AND WATER DUE TO SELF NEGLECT

For peer review only

Rates, causes, place, and predictors of mortality in adults with intellectual disabilities with and without Down syndrome: cohort study with record linkage

OTHER SPECIFIED GENERAL SYMPTOMS AND SIGNS
OTHER ILL-DEFINED AND UNSPECIFIED CAUSES OF MORTALITY
EXPOSURE TO UNSPECIFIED FACTOR
MULTI-SYSTEM DEGENERATION
BENIGN NEOPLASM, MENINGES, UNSPECIFIED
AGRANULOCYTOSIS
SARCOIDOSIS OF OTHER AND COMBINED SITES
SARCOIDOSIS, UNSPECIFIED
HYPOPITUITARISM
HYPOTHYROIDISM, UNSPECIFIED
OTHER THYROTOXICOSIS
VOLUME DEPLETION

Injuries and external causes

Injuries and accidents

INTRACRANIAL INJURY, UNSPECIFIED
UNSPECIFIED INJURY OF HEAD
INJURY OF COLON
FRACTURE OF NECK OF FEMUR
FRACTURE OF SHAFT OF TIBIA
UNSPECIFIED MULTIPLE INJURIES
FAT EMBOLISM (TRAUMATIC)
SEQUELAE OF UNSPECIFIED INJURY OF HEAD
UNSPECIFIED FALL
SEQUELAE OF OTHER ACCIDENTS

Medical/surgical complication

POISONING BY OTHER ANTIDYSRHYTHMIC DRUGS, NOT ELSEWHERE CLASSIFIED
ADVERSE EFFECTS OF OTHER ANTIDYSRHYTHMIC DRUGS, NOT ELSEWHERE CLASSIFIED
ABN REACT TO/LATER COMPLIC OF OP WITH IMPLANT OF ARTIFICIAL INTERN DEVICE
ABN REACT TO/LATER COMPLIC OF OP WITH ANASTOMOSIS, BYPASS OR GRAFT
ABN REACT TO/LATER COMPLIC OF OP WITH FORMATION OF EXTERNAL STOMA
ABNORMAL REACTION TO OR LATER COMPLICATION OF OTHER MEDICAL PROCEDURES
SEQ OF PROCED CAUSING ABN REACT/COMPLIC,W/O MENTION OF MISADV AT THE TIME
OTHER POSTPROCEDURAL RESPIRATORY DISORDERS

For peer review only

Rates, causes, place, and predictors of mortality in adults with intellectual disabilities with and without Down syndrome: cohort study with record linkage

Supplementary table 3. Predictors of the outcome time to death from univariate analyses

Variable		N with event/ N in group	Hazard ratio (95% CI)	Individual p-value	Overall p-value
Demographics					
Age		294/961	1.05 (1.04, 1.06)	<0.0001	
Sex	Male	154/525	0.88 (0.70, 1.11)	0.2730	
	Female	140/436	1.00 (-)		
Ability level	Mild ID	92/382	1.00 (-)		0.0007
	Moderate ID	73/236	1.38 (1.01, 1.87)	0.0411	
	Severe ID	67/180	1.75 (1.28, 2.40)	0.0005	
	Profound ID	62/163	1.77 (1.28, 2.45)	0.0005	
Type of accommodation	Family carer	70/374	1.00 (-)		<0.0001
	Independent of care	36/93	2.35 (1.57, 3.52)	<0.0001	
	Paid support	161/435	2.18 (1.65, 2.88)	<0.0001	
	Congregate	27/59	2.87 (1.84, 4.48)	<0.0001	
Neighbourhood deprivation	1 – most affluent	18/73	1.00 (-)		0.1890
	2	56/137	1.92 (1.13, 3.27)	0.0158	
	3	10/45	0.90 (0.42, 1.95)	0.7896	
	4	10/40	1.06 (0.49, 2.30)	0.8808	
	5	12/32	1.71 (0.82, 3.55)	0.1527	
	6	9/32	1.27 (0.57, 2.82)	0.5640	
	7	9/34	1.09 (0.49, 2.43)	0.8302	
	8	15/58	1.21 (0.61, 2.41)	0.5818	
	9	35/124	1.22 (0.69, 2.16)	0.4882	
	10 - most deprived	120/386	1.41 (0.86, 2.31)	0.1782	
Civil status	Single	288/938	1.28 (0.57, 2.87)	0.5485	
	Not single	6/23	1.00 (-)		
Employment/day activities	Yes	83/231	1.33 (1.03, 1.71)	0.0284	
	No	211/730	1.00 (-)		
Smoker	Yes	46/101	1.70 (1.24, 2.33)	0.0009	
	No	248/860	1.00 (-)		
Down syndrome	Yes	64/179	1.30 (0.98, 1.71)	0.0673	
	No	230/782	1.00 (-)		
Epilepsy	Yes	111/325	1.25 (0.99, 1.58)	0.0636	
	No	183/636	1.00 (-)		
Spastic quadriplegia	Yes	24/325	1.67 (1.10, 2.54)	0.0158	
	No	183/636	1.00 (-)		
Impaired mobility	Yes	195 /735	0.51 (0.40, 0.65)	<0.0001	
	No	99 /226	1.00 (-)		
Body mass index	Underweight	9/43	0.63 (0.32, 1.25)	0.1847	0.1865
	Acceptable	83/265	1.00 (-)		
	Overweight	75/289	0.78 (0.57, 1.06)	0.1132	
	Obese	81/237	1.08 (0.80, 1.47)	0.6152	
	Morbidly obese	16/58	0.87 (0.51, 1.48)	0.6058	
Hearing impairment	Yes	112/267	1.79 (1.41, 2.26)	<0.0001	
	No	182/694	1.00 (-)		
Visual impairment	Yes	154/449	1.29 (1.02, 1.62)	0.0317	
	No	140/512	1.00 (-)		

Rates, causes, place, and predictors of mortality in adults with intellectual disabilities with and without Down syndrome: cohort study with record linkage

Urinary incontinence	Yes	158/632	0.52 (0.41, 0.65)	<0.0001	
	No	136/329	1.00 (-)		
Bowel incontinence	Yes	197/733	0.55 (0.43, 0.70)	<0.0001	
	No	97/228	1.00 (-)		
Diabetes	Yes	29/47	2.72 (1.86, 4.00)	<0.0001	
	No	265/914	1.00 (-)		
PEG/tube fed	Yes	N/7	4.99 (2.22, 11.20)	0.0001	
	No	288/954			
Constipation	Yes	112/316	1.34 (1.06, 1.70)	0.0145	
	No	182/645	1.00 (-)		
Ataxia/gait disorder	Yes	104/276	1.50 (1.18, 1.90)	0.0009	
	No	190/685	1.00 (-)		
Nail disorder	Yes	74/223	1.18 (0.91, 1.54)	0.2120	
	No	220/738	1.00 (-)		
Epidermal thickening	Yes	66/207	1.10 (0.84, 1.45)	0.4947	
	No	228/754	1.00 (-)		
Cerebral palsy	Yes	54/175	1.02 (0.76, 1.37)	0.8792	
	No	240/786	1.00 (-)		
Osteoporosis	Yes	76/174	1.71 (1.32, 2.22)	<0.0001	
	No	218/786	1.00 (-)		
Fungal infection	Yes	42/158	0.83 (0.61, 1.18)	0.3366	
	No	252/803	1.00 (-)		
Hypertension	Yes	56/146	1.36 (1.01, 1.82)	0.0399	
	No	238/815	1.00 (-)		
Dysphagia	Yes	51/132	1.51 (1.11, 2.04)	0.0080	
	No	243/829	1.00 (-)		
Dyspnoea	Yes	49/130	1.41 (1.04, 1.92)	0.0285	
	No	245/831	1.00 (-)		
Muskuloskeletal pain	Yes	48/148	1.14 (0.83, 1.55)	0.4153	
	No	246/813	1.00 (-)		
Bone deformity	Yes	50/139	1.32 (0.97, 1.79)	0.0769	
	No	244/822	1.00 (-)		
Dental/oral problem	Yes	38/120	1.07 (0.76, 1.50)	0.7128	
	No	256/841	1.00 (-)		
Eczema/dermatitis	Yes	38/138	0.86 (0.61, 1.21)	0.3790	
	No	256/823	1.00 (-)		
GORD	Yes	51/133	1.43 (1.06, 1.94)	0.0198	
	No	243/828	1.00 (-)		
Lower respiratory tract infection	Yes	55/126	1.75 (1.30, 2.34)	0.0002	
	No	239/835	1.00 (-)		
Total number of physical conditions		294/961	1.06 (1.04, 1.08)	<0.0001	
Psychosis	Yes	11 /42	0.81 (0.44, 1.48)	0.4990	
	No	283 /919	1.00 (-)		
Affective disorder including bipolar	Yes	24/68	1.19 (0.78, 1.80)	0.4216	
	No	270/893	1.00 (-)		
Autism	Yes	13/69	0.54 (0.31, 0.94)	0.0306	
	No	281/892	1.00 (-)		
Problem behaviour	Yes	71/218	1.09 (0.83, 1.42)	0.5251	
	No	223/743	1.00 (-)		
Eating disorder, including pica	Yes	5/17	0.99 (0.41, 2.40)	0.9857	
	No	289/944	1.00 (-)		
Any mental illness, excluding problem behaviours	Yes	73/217	1.16 (0.89, 1.51)	0.2849	
	No	221/744	1.00 (-)		
Service use					
Number of GP consultations in last 12 months		287/951	1.05 (1.03, 1.06)	<0.0001	

Number of A&E attendances in last 12 months		280/938	1.09 (0.99, 1.20)	0.0847	
Number of health professions providing care		294 /961	1.10 (1.03, 1.16)	0.0023	
Prescriptions					
Antipsychotics	Yes	79/226	1.12 (0.94, 1.57)	0.1421	
	No	215/735	1.00 (-)		
Antidepressants	Yes	39/118	1.16 (0.83, 1.63)	0.3778	
	No	255/843	1.00 (-)		
Anxiolytic/hypnotics	Yes	20/68	0.95 (0.60, 1.49)	0.8159	
	No	274/893	1.00 (-)		
Antiepileptics	Yes	90/253	1.31 (1.02, 1.68)	0.0315	
	No	204/708	1.00 (-)		
Number of drug classes taken		294/961	1.16 (1.12, 1.21)	<0.0001	

A&E=accident and emergency; CI=confidence interval; GORD=gastro-oesophageal reflux disorder; PEG=percutaneous endoscopic gastrostomy

For peer review only

STROBE Statement—checklist of items that should be included in reports of observational studies

	Item No.	Recommendation	Page No.	Relevant text from manuscript
**Title and abstract**	1	(*a*) Indicate the study's design with a commonly used term in the title or the abstract		p1
		(*b*) Provide in the abstract an informative and balanced summary of what was done and what was found		p2
**Introduction**				
Background/rationale	2	Explain the scientific background and rationale for the investigation being reported		p4-6, supplementary table 1
Objectives	3	State specific objectives, including any prespecified hypotheses		6, paragraph 3
**Methods**				
Study design	4	Present key elements of study design early in the paper		p6-10, supplementary tables 2/3
Setting	5	Describe the setting, locations, and relevant dates, including periods of recruitment, exposure, follow-up, and data collection		p7, paragraph 1, 7-8
Participants	6	(*a*) *Cohort study*—Give the eligibility criteria, and the sources and methods of selection of participants. Describe methods of follow-up<br>*Case-control study*—Give the eligibility criteria, and the sources and methods of case ascertainment and control selection. Give the rationale for the choice of cases and controls<br>*Cross-sectional study*—Give the eligibility criteria, and the sources and methods of selection of participants		p7, paragraph 1
		(*b*) *Cohort study*—For matched studies, give matching criteria and number of exposed and unexposed<br>*Case-control study*—For matched studies, give matching criteria and the number of controls per case		p7, paragraph 2, p9, paragraph 4
Variables	7	Clearly define all outcomes, exposures, predictors, potential confounders, and effect modifiers. Give diagnostic criteria, if applicable		p7-8, supplementary table 2
Data sources/ measurement	8*	For each variable of interest, give sources of data and details of methods of assessment (measurement). Describe comparability of assessment methods if there is more than one group		p7-8, p9, paragraph 4
Bias	9	Describe any efforts to address potential sources of bias		p9, paragraph 4
Study size	10	Explain how the study size was arrived at		P7, paragraph2, p9, paragraph 4

Continued on next page

For peer review only - http://bmjopen.bmj.com/site/about/guidelines.xhtml

For peer review only

Quantitative variables	11	Explain how quantitative variables were handled in the analyses. If applicable, describe which groupings were chosen and why	p8-10
Statistical methods	12	(*a*) Describe all statistical methods, including those used to control for confounding	p8-10
		(*b*) Describe any methods used to examine subgroups and interactions	p8-10
		(*c*) Explain how missing data were addressed	p11, paragraph 2
		(*d*) *Cohort study*—If applicable, explain how loss to follow-up was addressed<br>*Case-control study*—If applicable, explain how matching of cases and controls was addressed<br>*Cross-sectional study*—If applicable, describe analytical methods taking account of sampling strategy	p11, paragraph 2
		(*e*) Describe any sensitivity analyses	N/A
**Results**			
Participants	13*	(a) Report numbers of individuals at each stage of study—eg numbers potentially eligible, examined for eligibility, confirmed eligible, included in the study, completing follow-up, and analysed	p11, paragraph 2
		(b) Give reasons for non-participation at each stage	p11, paragraph 2
		(c) Consider use of a flow diagram	-
Descriptive data	14*	(a) Give characteristics of study participants (eg demographic, clinical, social) and information on exposures and potential confounders	p11-12, Table 1, supplementary table 3
		(b) Indicate number of participants with missing data for each variable of interest	table 1, supplementary table 3
		(c) *Cohort study*—Summarise follow-up time (eg, average and total amount)	p12, paragraph 1
Outcome data	15*	*Cohort study*—Report numbers of outcome events or summary measures over time	p11, paragraph 2
		Case-control study—Report numbers in each exposure category, or summary measures of exposure	
		Cross-sectional study—Report numbers of outcome events or summary measures	
Main results	16	(*a*) Give unadjusted estimates and, if applicable, confounder-adjusted estimates and their precision (eg, 95% confidence interval). Make clear which confounders were adjusted for and why they were included	P15, table 6
		(*b*) Report category boundaries when continuous variables were categorized	N/A
		(*c*) If relevant, consider translating estimates of relative risk into absolute risk for a meaningful time period	-

Continued on next page

For peer review only

Other analyses	17	Report other analyses done—eg analyses of subgroups and interactions, and sensitivity analyses	p11-16
**Discussion**			
Key results	18	Summarise key results with reference to study objectives	p16, paragraph 2
Limitations	19	Discuss limitations of the study, taking into account sources of potential bias or imprecision. Discuss both direction and magnitude of any potential bias	p20, paragraph 1
Interpretation	20	Give a cautious overall interpretation of results considering objectives, limitations, multiplicity of analyses, results from similar studies, and other relevant evidence	p20, paragraph 2
Generalisability	21	Discuss the generalisability (external validity) of the study results	p20, paragraph 1
**Other information**			
Funding	22	Give the source of funding and the role of the funders for the present study and, if applicable, for the original study on which the present article is based	P24

*Give information separately for cases and controls in case-control studies and, if applicable, for exposed and unexposed groups in cohort and cross-sectional studies.

Note: An Explanation and Elaboration article discusses each checklist item and gives methodological background and published examples of transparent reporting. The STROBE checklist is best used in conjunction with this article (freely available on the Web sites of PLoS Medicine at http://www.plosmedicine.org/, Annals of Internal Medicine at http://www.annals.org/, and Epidemiology at http://www.epidem.com/). Information on the STROBE Initiative is available at www.strobe-statement.org.

For peer review only

For peer review only - http://bmjopen.bmj.com/site/about/guidelines.xhtml

BMJ Open

Rates, causes, place, and predictors of mortality in adults with intellectual disabilities with and without Down syndrome: cohort study with record linkage

Journal:	*BMJ Open*
Manuscript ID	bmjopen-2019-036465.R1
Article Type:	Original research
Date Submitted by the Author:	10-Apr-2020
Complete List of Authors:	Cooper, Sally-Ann; University of Glasgow, Institute of Health and Wellbeing<br>Allan, Linda; University of Glasgow, Institute of Health and Wellbeing<br>Greenlaw, Nicola; University of Glasgow, Robertson Centre for Biostatistics, Institute of Health and Wellbeing<br>Mcskimming, Paula; University of Glasgow, Robertson Centre for Biostatistics, Institute of Health and Wellbeing<br>Jasilek, Adam; University of Glasgow, Robertson Centre for Biostatistics, Institute of Health and Wellbeing<br>Henderson, Angela; University of Glasgow, Institute of Health and Wellbeing<br>McCowan, Colin ; University of St. Andrews<br>Kinnear, Deborah; University of Glasgow, Institute of Health and Wellbeing<br>Melville, Craig; University of Glasgow, Institute of Health and Wellbeing
**Primary Subject Heading**:	Public health
Secondary Subject Heading:	Mental health, Epidemiology
Keywords:	PUBLIC HEALTH, Adult psychiatry < PSYCHIATRY, EPIDEMIOLOGY, GENERAL MEDICINE (see Internal Medicine)

SCHOLARONE™
Manuscripts

I, the Submitting Author has the right to grant and does grant on behalf of all authors of the Work (as defined in the below author licence), an exclusive licence and/or a non-exclusive licence for contributions from authors who are: i) UK Crown employees; ii) where BMJ has agreed a CC-BY licence shall apply, and/or iii) in accordance with the terms applicable for US Federal Government officers or employees acting as part of their official duties; on a worldwide, perpetual, irrevocable, royalty-free basis to BMJ Publishing Group Ltd ("BMJ") its licensees and where the relevant Journal is co-owned by BMJ to the co-owners of the Journal, to publish the Work in this journal and any other BMJ products and to exploit all rights, as set out in our licence.

The Submitting Author accepts and understands that any supply made under these terms is made by BMJ to the Submitting Author unless you are acting as an employee on behalf of your employer or a postgraduate student of an affiliated institution which is paying any applicable article publishing charge ("APC") for Open Access articles. Where the Submitting Author wishes to make the Work available on an Open Access basis (and intends to pay the relevant APC), the terms of reuse of such Open Access shall be governed by a Creative Commons licence – details of these licences and which Creative Commons licence will apply to this Work are set out in our licence referred to above.

Other than as permitted in any relevant BMJ Author's Self Archiving Policies, I confirm this Work has not been accepted for publication elsewhere, is not being considered for publication elsewhere and does not duplicate material already published. I confirm all authors consent to publication of this Work and authorise the granting of this licence.

For peer review only

Rates, causes, place, and predictors of mortality in adults with intellectual disabilities with and without Down syndrome: cohort study with record linkage

*Sally-Ann Cooper

Linda Allan

Nicola Greenlaw

Paula McSkimming

Adam Jasilek

Angela Henderson

Colin McCowan

Deborah Kinnear

Craig Melville

*Corresponding author: Sally-Ann.Cooper@glasgow.ac.uk

For peer review only

Abstract

Objectives

To investigate mortality in adults with intellectual disabilities: rates, causes, place, demographic and clinical predictors.

Design

Cohort study with record linkage to death data.

Setting

General community.

Participants

961/1,023 (94%) adults (16-83 years; mean=44.1 years; 54.6% male) with intellectual disabilities, clinically examined in 2001-2004; subsequently record-linked to their National Health Service number, allowing linkage to death certificate data, 2018.

Outcome measures

Standardised mortality ratios (SMRs), underlying, and all contributing causes of death, avoidable deaths, place, and demographic and clinical predictors of death.

Results

294/961 (30.6%) had died; 64/179 (35.8%) with Down syndrome, 230/783 (29.4%) without Down syndrome. SMR overall=2.24 (1.98, 2.49); Down syndrome adults=5.28 (3.98, 6.57), adults without Down syndrome=1.93 (1.68, 2.18); male=1.69 (1.42, 1.95), female=3.48 (2.90, 4.06). SMRs decreased as age increased. More severe intellectual disabilities increased SMR, but ability was not retained in the multivariable model. SMRs were higher for most ICD-10 chapters. For adults without Down syndrome, aspiration/reflux/choking, and respiratory infection were the commonest underlying causes of mortality; for Down syndrome adults “Down syndrome”, and dementia were most common. Amenable deaths (29.8%) were double that in the general population (14%). 60.3% died in hospital. Mortality risk related to: percutaneous endoscopic gastrostomy/tube fed, Down syndrome, diabetes, lower respiratory tract infection at

For peer review only

cohort-entry, smoking, epilepsy, hearing impairment, increasing number of prescribed drugs, increasing age. Bowel incontinence reduced mortality risk.

Conclusions

Adults with intellectual disabilities with and without Down syndrome have different SMRs and causes of death which should be separately reported. Both die younger, from different causes than other people. Some mortality risks are similar to other people, with earlier mortality reflecting more multi-morbidity; amenable deaths are also common. This should inform actions to reduce early mortality, e.g. training to avoid aspiration/choking, pain identification to address problems before they are advanced, and reasonable adjustments to improve health-care quality.

Strengths and limitations of this study

- Thorough methods of case ascertainment for intellectual disabilities at baseline.
- Individual verification of intellectual disabilities and its severity, and detailed health assessments at baseline.
- Longitudinal design.
- Large cohort size and study duration, and successful record linkage for 94% of participants.
- Limitations include that the study was conducted in only one part of Scotland, and the reliance upon recorded cause of death from death certificates.

Word count: 5,605

For peer review only - http://bmjopen.bmj.com/site/about/guidelines.xhtml

For peer review only

Introduction

People with intellectual disabilities die at a younger age than other people; on average, 20 years younger,[1] or 28 years younger specifically for people with Down syndrome.[2] It has been demonstrated that people with intellectual disabilities receive poorer management of their long term conditions within primary health care services compared with the general population,[3] and it is conceivable that this is one contributor to earlier mortality. It has been suggested that as many as 40% of deaths of people with intellectual disabilities may have been amenable to good quality health care.[4-6] There has been a recent increase in research on mortality in people with intellectual disabilities, but very little research has distinguished people with intellectual disabilities with and without Down syndrome, or investigated the factors associated with risk of mortality, and causes of mortality.

Previous studies on death in people with intellectual disabilities had limitations such as small sample sizes, or non-representative populations. More recently, there have been large-scale studies which are more representative, having been drawn from intellectual disabilities registers, or social security or primary care data with record linkage to death certification. These have been undertaken in parts of Sweden, Australia, England, Finland, Canada, Ireland, and USA (supplementary table 1).[5-19] These studies fairly consistently report standardised mortality ratios (SMRs) to be high for people with intellectual disabilities, more so at younger ages, and higher for women than men. Adult studies have tended to report SMRs in the region of 2-4, although in some, SMR is only slightly above 1.[10,16,19] However, direct comparison between studies is not always possible, due to the different age ranges studied and methods of reporting.

- Supplementary table 1 -

For peer review only - http://bmjopen.bmj.com/site/about/guidelines.xhtml

In view of the methods that studies have used for population identification (typically, routine administrative data linked to death certifications), they provide little information on the socio-clinical factors that influence SMR, or the risk factors associated with death, beyond that of age and sex. Three studies reported SMR by level of intellectual disabilities, with, broadly speaking, higher SMR with more severe intellectual disabilities.[7,10,17] Only three studies (different studies to those that reported on level of intellectual disabilities) were able to report data separately for adults with intellectual disabilities with and without Down syndrome; two found higher mortality rates for adults with Down syndrome (SMR=7.6,[9] and hazard ratio=9.21[5]) than for adults without Down syndrome, or an odds ratio showing Down syndrome as a risk of death.[12] A further study reported SMR=5.5 for children and adults (combined) with Down syndrome, but did not report SMR for those with intellectual disabilities without Down syndrome.[20] Two studies reported adults with intellectual disabilities to have higher SMRs if they have the co-morbidities of epilepsy,[5,7] and cerebral palsy,[7] as opposed to not having these comorbidities. One study reported adults with intellectual disabilities with comorbid autism to have lower risk of mortality than those without comorbid autism.[5] One study reported the risk factors for mortality in a population with intellectual disabilities to be: age, Down syndrome, cerebral palsy, blindness/low vision, technological dependence/medical fragility, wheelchair dependence, mobility impairment without wheelchair dependence, and epilepsy.[12] Factors not found to be risks, if any, were not reported, and a further limitation was that factors were reported by agency staff, rather than the individuals undergoing health assessments.[12] We have not identified any other studies that investigated risk factors for time to mortality in adults with intellectual disabilities.

There is less consistency regarding the most common certified underlying causes of death in adults with intellectual disabilities, partly as some studies do not report these separately for children and adults, or by age ranges. Additionally, studies group causes of death in different ways (e.g. pneumonia versus respiratory system), which can affect

For peer review only - http://bmjopen.bmj.com/site/about/guidelines.xhtml

prevalence rankings between studies. Pneumonia, other respiratory diseases, and diseases of the nervous system were reported to be the most common in one study,[11] diseases of the circulatory system and respiratory systems in another,[5] heart disease, neoplasm, and Alzheimer disease in a third,[18] and diseases of the circulatory system, neoplasm, and the nervous system in a fourth.[19] In adults with intellectual disabilities, cause specific SMRs have been reported to be high across most groups of disorders.[5,11] These studies did not report cause of death separately for adults with and without Down syndrome. Given the different health profile of people with Down syndrome compared with people with intellectual disabilities of other causes, this is an important limitation.[21] In people with Down syndrome, most studies on mortality have been conducted with child populations, and report the most common causes of death to be congenital heart disease, and pneumonia/diseases of the respiratory system.[2]

Overall, the existing body of literature on mortality in adults with intellectual disabilities does not include more detailed information on level of intellectual disabilities, nor separate out the population with, from those without, Down syndrome (for whom causes of death may differ), nor investigate health and demographic predictors of death other than age and sex, and is inconsistent with regards to causes of death. A better understanding of these factors may provide a pathway to action to reduce the observed earlier mortality in adults with intellectual disabilities.

This study aims to investigate the rates, causes, place, and demographic and clinical associations with mortality in adults with intellectual disabilities, with and without Down syndrome.

Methods

Approval

For peer review only - http://bmjopen.bmj.com/site/about/guidelines.xhtml

Ethical approved was gained from NHS Greater Glasgow Primary Care Trust - Community & Mental Health Research Ethics Committee, and NHS Greater Glasgow and Clyde Safe Haven. Individual consent to participate was taken in line with Scottish law, between 2001-2004.

Participants

The adult (aged 16+ years) intellectual disabilities population living within the NHS Greater Glasgow area was identified through multiple sources between 2000-2001. General practitioners were financially incentivised to identify their registered patients with intellectual disabilities, and all 631 (100%) did so. Adults were also identified via the intellectual disabilities health and social work services including day services, the Health Board register, and records of financial payments for any service by social work. This process led initially to an over-identification, such as people with IQ scores in the 70–80 range with additional complex health needs. All were systematically reviewed by nurses in the intellectual disabilities health service, and this group were removed. Thus, a register was compiled, and subsequently updated annually via general practices, with central support from the intellectual disabilities health service, until 2017 when services were redesigned. The identified adult prevalence of intellectual disabilities within the area was 3.33 per 1,000 in 2000-2001.

Process and data collection

With initial piloting in 2001, each participant had a detailed assessment of their general and mental health, and demographic factors, completed 2002-2004. One of six specially trained, registered nurses reviewed each person's primary health care records, then used a semi-structured tool, the C21st Health Check, to assess clinical factors and the level and cause of intellectual disabilities. In addition to a review of existing health problems and all bodily health systems, a physical examination was undertaken, including assessment of vision and hearing, measurement of height and weight, and a phlebotomy protocol followed. All information was then reviewed by the nurse with one

For peer review only - http://bmjopen.bmj.com/site/about/guidelines.xhtml

of three general practitioners with a special interest in intellectual disabilities, and any further investigations that were indicated were completed. Previously known, and newly identified, conditions were then classified using the *International Statistical Classification of Diseases and Related Health Problems, 10th Revision (ICD-10).*[22] Anyone identified to have possible, probable, or definite mental ill-health, autism, or problem behaviours was then fully assessed by the project's intellectual disabilities psychiatrists. Each person's assessment findings were then case conferenced by the two Consultant psychiatrists, and diagnoses were derived and agreed according to clinical diagnoses, *ICD-10 (Diagnostic Criteria for Research),*[23] *Diagnostic and Statistical Manuel of Mental Disorders-IV-TR,*[24] and *Diagnostic Criteria for Psychiatric Disorders for use with Adults with Learning Disabilities (DC-LD).*[25] Information was also collected on demographics, and community, hospital, and social service use. Further details are provided elsewhere.[26,27] The data were entered into a database by two dedicated data-entry staff.

Each person in Scotland is given a number unique to them at birth or first registration with a general practitioner, which is used in almost all subsequent health service encounters, and on certification of death. The numbers are held on the Community Health Index (CHI) database at National Services Scotland. These CHI numbers provided a means to record link each participant with National Records for Scotland death certification data. This linkage was performed in 2018, and the linked data were held in the NHS Greater Glasgow & Clyde (NHS GG&C) Safe Haven. Data on immediate, underlying, and contributory causes of deaths by ICD-10 codes, age at death, and place of death were extracted.

In order to provide finer granularity of cause of death, two clinical academics then grouped specific causes of death into narrower groupings than those provided by ICD-10 chapter headings (supplementary table 2). This approach was also in view of the recognised issue of variation between health staff in distinguishing and recording immediate causes of death, and because some causes occurred in low numbers so could

For peer review only - http://bmjopen.bmj.com/site/about/guidelines.xhtml

not be individually reported due to the risk of statistical disclosure. Additionally, some conditions likely to be the same are spilt between different ICD-10 chapters, e.g. dementia in Alzheimer disease (F00) and unspecified dementia (F03) in the ICD-10 mental and behavioural disorders chapter, and Alzheimer's disease (G30) and Alzheimer's disease, unspecified (G30.9) in the ICD-10 diseases of the nervous system chapter. A list of related conditions was generated by one of the clinical academics and then checked by the second.

- Supplementary table 2 –

Analyses

All statistical analyses were conducted using R for Windows v3.3.0 or SAS 9.3 (SAS Institute, Cary NC) and were performed within the NHS GG&C Safe Haven environment. Due to disclosure principles of the Safe Haven, results with counts of less than 5 cannot be released; these have been referred to as <5 throughout. Similarly, if it is deemed possible that participants may be identified from the results, these may be omitted. Details are provided if this occurred.

Data were summarised for the population of adults aged 16+ years with intellectual disabilities. Categorical variables were summarised with the number and percentages of people falling into each category and the number of missing data. Continuous variables were summarised with the number of observations and those missing, the mean and standard deviation (SD), and the minimum and maximum values, unless otherwise stated.

Participant characteristics were summarised overall and for those alive and those deceased. For those who are deceased, their data including age at death, underlying/contributing causes of death, and location of death were summarised for those with and without Down syndrome. Location codes for place of death are provided

For peer review only - http://bmjopen.bmj.com/site/about/guidelines.xhtml

For peer review only

where available. We assumed those with the code for non-institutional location to have died at home. Due to small numbers, location codes have been grouped together for NHS hospitals, home, and other hospitals/care facilities including hospices.

Mortality incidence rates have been calculated using the number of deaths in the cohort divided by the number of person years alive within the study period multiplied by 100,000, overall and for those with and without Down syndrome. SMRs were calculated using population data for those aged 15 and over within NHS GG&C in 2010.[28,29] Death rates for males and females by 5 year band ages groups spanning from 15-20 years old to 90 years and over were summed to form the expected death rates for the general population. The observed death rate for adults with intellectual disabilities was taken from our study results. The observed/expected death rates were calculated for the intellectual disabilities cohort overall then separately by age group, sex, ability level, and for the adults with, and without, Down syndrome, and ICD-10 chapter for cause of death, and compared to the general population.

Deaths were also analysed for those that could be considered as deaths that would have been avoidable. The Office for National Statistics (ONS) published a definition of avoidable mortality,[30] which lists the causes of amenable deaths (deaths that should not occur in the presence of good health care, e.g. respiratory disease), and causes of preventable deaths (e.g. from diseases that could have been avoided by prior immunisation), by ICD-10 codes. Causes of death for the adults with intellectual disabilities have been summarised by ONS definition of avoidable deaths.

To determine the demographic and clinical factors associated with death in adults with intellectual disabilities, time to event analyses were explored using univariate Cox Proportional Hazards models. Variables were selected as potentially relevant on the basis of what is known on causes of death in people with intellectual disabilities, the 20 most common physical health conditions reported in the adult population with intellectual

For peer review only - http://bmjopen.bmj.com/site/about/guidelines.xhtml

disabilities,[21] and other factors hypothesised as potentially clinically relevant (supplementary table 3):

- Demographics - 9 variables.
- Clinical conditions - 33 variables.
- Service use - 3 variables.
- Prescriptions - 5 variables.

All 50 variables were then permitted entry in to a single multivariable analysis using stepwise regression methods, in order to identify a model containing the statistically significant factors associated with death. Age at date of the health assessment was entered in to the model as a continuous measure. Results from the univariate Cox Proportional Hazards models (Supplementary table 3) and the statistically significant multivariable model from the stepwise results have been presented with hazard ratios with corresponding 95% confidence intervals (HR, 95% CI) and p-values were obtained.

- Supplementary table 3 –

Patient and public involvement

This study was designed to respond to the growing concern expressed by people with intellectual disabilities, their families, and third sector organisations about the early deaths of people with intellectual disabilities. The Scottish Learning Disabilities Observatory, where this research was undertaken, has a specific remit for people with intellectual disabilities. Its steering group includes partners from third sector organisations, including Down syndrome Scotland, and people with intellectual disabilities, who approved the work plan for this project prior to it commencing. Results from this study will be disseminated for people with intellectual disabilities in an easy-read version via the Scottish Learning Disabilities Observatory.

Results

For peer review only - http://bmjopen.bmj.com/site/about/guidelines.xhtml

Population characteristics

962 of the original 1,023 (94.0%) adults with intellectual disabilities who were assessed were linked to a CHI number enabling the extraction of relevant death data. Reasons for the unlinked 61 people could be due to moving out of the area, or a recording mistake. 1 further participant was removed from the analysis due to inaccurate recording of dates, leaving 961 adults in the cohort (93.9%). Of these 961 adults, 294 (30.6%) had a record of death. Table 1 shows the baseline characteristics of the full cohort of 961, the adults who died, and those still alive at the time of linkage.

- Insert table 1 about here –

Age at death, and mortality incidence

The mean age at death was 61.0 years (SD=7.0 years). Of the 961 adults, 64 (35.8%) of the 179 adults with Down syndrome, and 230 (29.4%) of the 782 adults without Down syndrome had a record of death. Their mean age of death was 56.9 years (SD=4.3 years) for the adults with Down syndrome, and 62.2 years (SD=7.5 years) for the adults without Down syndrome. Mortality incidence for the cohort during the study period was 3,049.0 per 100,000 person years follow-up, with 3,832.1 per 100,000 for those with Down Syndrome and 2,885.0 for those without Down syndrome.

Standardised mortality ratios

Compared with the general population, the SMR was 2.24 (1.98, 2.49) overall; 5.28 (3.98, 6.57) for adults with Down syndrome, 1.93 (1.68, 2.18) for adults without Down syndrome; 1.69 (1.42, 1.95) for men and 3.48 (2.90, 4.06) for women. SMRs were higher the more severe the level of intellectual disabilities, with people with profound intellectual disabilities having an SMR of 4.14 (3.11, 5.17). SMR was high for all age groups (though for the 15-25 year age group, the wide confidence interval includes one, perhaps due to the smaller number of deaths in this group); this decreased as age increased. SMRs were high for most ICD-10 chapter groups of conditions, particularly so

For peer review only - http://bmjopen.bmj.com/site/about/guidelines.xhtml

for congenital malformations at 17.26 (10.75, 23.78), diseases of the digestive system at 16.13 (8.23, 24.04), mental and behavioural disorders at 12.64 (3.27, 22.00), and external causes at 11.08 (3.40, 18.76). Details are shown in table 2.

- Insert table 2 about here –

Causes of death

Cause of death data was available from death certificates for 262 (89.1%) of 294 participants who had died, which include 57 (89.1%) participants with Down syndrome, and 205 (88.7%) participants without Down syndrome. Table 3 shows the underlying causes of death by ICD-10 chapters separately for the adults with, and without Down syndrome. For the whole cohort, diseases of the respiratory system were the most common (21.8%), then diseases of the circulatory system (19.1%), then diseases of the nervous system (13.0%), and neoplasms, followed by congenital anomalies (10.3%). For the adults with Down syndrome, congenital anomalies were the most common (in all cases this was a record of "Down syndrome"), then jointly diseases of the respiratory system and diseases of the circulatory system, then diseases of the nervous system, followed by infections, and mental and behavioural disorders. For the adults without Down syndrome, diseases of the respiratory system were the most common, then diseases of the circulatory system, then neoplasms, then diseases of the nervous system, followed by diseases of the digestive system. Table 4 presents the most common underlying causes of death by individual causes, or related groups of causes, with finer granularity than ICD-10 chapter headings (groups are shown in supplementary table 2). Causes are listed in the order of how common they were in the whole cohort. Data are presented separately for the adults with, and without Down syndrome. For the whole cohort, the most common cause was aspiration/reflux/choking, then respiratory infection, then other malignancy (non gastrointestinal), then other condition (mostly unrelated conditions that could not be reported individually or as groups, due to individually occurring at a frequency of <5). For the adults with Down syndrome, Down

For peer review only - http://bmjopen.bmj.com/site/about/guidelines.xhtml

syndrome was the most common cause, then dementia, then other infection. For the adults without Down syndrome, aspiration/reflux/choking was the most common cause, then respiratory infection, then other malignancy (non gastrointestinal). For the 21 people whose death certificate listed Down syndrome as their underlying cause of death, the death certificates were reviewed and underlying cause of death reclassified, as a sensitivity check. Following this, the most common underlying causes of death for the adults with Down syndrome were dementia (n=20; 35.1%), then other infection (n=7; 12.3%).

- Insert tables 3 and 4 about here -

Table 5 shows the all contributing causes of death data, again presenting the most common causes by individual causes, or related groups of causes with finer granularity than ICD-10 chapter headings. Data is presented separately for the adults with, and without Down syndrome. For the whole cohort, respiratory infection was the most common cause (27.1%), followed by aspiration/reflux/choking (19.8%), other conditions (15.6%), other cardiovascular conditions (non acute myocardial nor other ischaemic heart disease: 14.5%), then other respiratory conditions. For the adults with Down syndrome, Down syndrome was the most common, then dementia, then respiratory infection, then aspiration/reflux/choking. For the adults without Down syndrome, respiratory infection was the most common cause, then aspiration/reflux/choking, then other condition, then other respiratory conditions, and intellectual disabilities.

- Insert table 5 about here –

Avoidable deaths

According to the ONS list of avoidable deaths, 102 (38.9%) of the 262 deaths were avoidable; most notably, respiratory infection and epilepsies (table 4). 78 (29.8%) were deaths that are amenable to good health care, whilst 51 (19.5%) were preventable

For peer review only - http://bmjopen.bmj.com/site/about/guidelines.xhtml

deaths. 27 (10.3%) deaths were classed as both amenable and preventable deaths. This compares to published Scottish death data showing in 2018 that 28% of deaths were avoidable; 14% amenable and 24% preventable, similar to the figures in the previous four years (data not available prior to 2014).[31] For the 57 deaths of adults with Down syndrome, 17 (29.8%) deaths were avoidable, 15 (26.3%) deaths were amenable to good health care, whilst 7 (12.3%) were preventable. 5 (8.8%) were both amenable and preventable. For the 205 deaths of adults without Down syndrome, 85 (41.5%) were avoidable, 63 (30.7%) deaths were amenable to good health care, whilst 44 (21.5%) were preventable. 22 (10.7%) were both amenable and preventable.

Place of death

Of the 262 participants for whom place of death was known, 158 (60.3%) died in an NHS Hospital, 70 (26.7%) died at home, and 34 (13.0%) died within other hospitals/care facilities. This was similar for both the adults with Down syndrome: 31 (54.4%) in an NHS hospital, 17 (29.8%) at home, and 9 (15.8%) within other hospitals/care facilities; and the adults without Down syndrome: 127 (62.0%) in an NHS hospital, 53 (25.9%) at home, and 25 (12.2%) within other hospitals/care facilities.

Factors associated with risk of death

The results from the univariate cox proportional hazards models indicated that of the original 50 potential variables, factors associated with risk of death were (supplementary table 3):

- Demographics – age at the time of the health assessment, more severe learning disabilities, accommodation type (not living with family carer), not having day-time occupation, and being a smoker (but not sex, the extent of neighbourhood deprivation, civil status, nor Down syndrome, in view of the confidence intervals).
- Clinical conditions – having spastic quadriplegia, hearing impairment, visual impairment, diabetes, percutaneous endoscopic gastrostomy/tube fed, constipation, ataxia/gait disorder, osteoporosis, hypertension, dysphagia, dyspnoea, gastro-

For peer review only - http://bmjopen.bmj.com/site/about/guidelines.xhtml

oesophageal reflux disorder, lower respiratory tract infection, total number of physical health disorders, not having impaired mobility, not having urinary incontinence, not having bowel incontinence, and not having autism (but not epilepsy, body mass index, nail disorder, epidermal thickening, cerebral palsy, fungal infection, musculoskeletal pain, bone deformity, dental/oral problem, eczema/dermatitis, psychosis, affective disorder including bipolar affective disorder, problem behaviour, eating disorder including pica, nor any mental illness).

- Service use – number of general practitioner consultations in the previous 12 months, total number of different types of health professionals providing care at the time of the clinical assessment, (but not number of accident and emergency attendances in the previous 12 months).
- Prescriptions – antiepileptic drugs, total number of different types of drugs, (but not antipsychotic drugs, antidepressant drugs, nor anxiolytic drugs).

Table 6 shows the final model of the variables retained in the multivariable analysis for time to death. The significant factors indicating an increased risk of death were increased age at the time of the health assessment, smoking, Down syndrome, diabetes, being percutaneous endoscopic gastrostomy/tube fed, lower respiratory tract infection at cohort inception, epilepsy, hearing impairment, and total number of different types of drugs prescribed, whilst bowel incontinence showed a reduced risk of death. Of note, level of intellectual disabilities whilst significant in the univariate analysis, was not retained in the multivariable model.

- Insert table 6 about here –

Discussion

Principle findings and interpretation

For peer review only - http://bmjopen.bmj.com/site/about/guidelines.xhtml

For peer review only

As far as we are aware, this is the first population-based study of adults with intellectual disabilities to report in detail the factors associated with time to death, and to describe their causes of death and quantify the SMR separately for adults with Down syndrome and adults without Down syndrome. This is important, since adults with Down syndrome form a notable proportion of all adults with intellectual disabilities (19% in this cohort), and because they have a different pattern of clinical conditions compared with other adults with intellectual disabilities.[21] We found that aspiration/reflux/choking is the most common underlying cause of death in adults with intellectual disabilities, followed by respiratory infection. They are also the most common all contributing causes of death. The profile differed in the adults with Down syndrome for whom "Down syndrome", followed by dementia, were recorded as the most common underlying cause of death, and all contributing causes of death (or alternatively, dementia, then other infection were the most common underlying causes when "Down syndrome" deaths were reclassified); with the next most common all contributing cause of death being respiratory infection, then aspiration/reflux/choking. The proportion of deaths that would have been amenable to good care for adults with intellectual disabilities was more than double that seen in the general population. Although aspiration/reflux/choking is not included in the ONS list of avoidable deaths, and therefore not included in the figures we report on amenable deaths, we consider that good care could have prevented many of these deaths. This appears to be very important for adults with intellectual disabilities irrespective of whether they have Down syndrome. Similarly, some other causes of deaths within this cohort (supplementary table 2), such as constipation/mega-colon, and urinary tract infections do not appear on the ONS list of avoidable deaths.

Clearly, this pattern of causes of death differs from that seen in the general population, in whom the most common underlying causes of death are heart disease, then dementia, then lung cancer in men, and dementia, then heart disease, then stroke in women.[32] When all cancers are grouped together, in the general population, cancer is the leading underlying cause of death in 30% of men and 26% of women, compared with this study

For peer review only - http://bmjopen.bmj.com/site/about/guidelines.xhtml

reporting 0% for adults with Down syndrome, and 15.2% for adults with intellectual disabilities without Down syndrome – presumably as the adults with intellectual disabilities are dying younger from other causes, and cancers increase with age.

We found an overall SMR of 2.24; 5.28 in the adults with Down syndrome and 1.93 for the adults without Down syndrome. SMRs were higher for most ICD-10 chapter groupings of conditions. It was higher in the women than the men, as has been previously reported in most (supplementary table 1), but not all[10,19] previous reports. The reason for this is unknown; in the general population, mortality rates have fallen in recent decades, and more so in middle and older aged men than women (i.e. the sex gap is narrowing at these ages), but we do not know what trends over time there have been for people with intellectual disabilities. Having intellectual disabilities removes differences in lifespan by sex compared with the general population; but sex was not a predictor of mortality in our study, so the SMR difference may only be because of the difference found in the general population by sex. SMRs were lowest with older age groups, likely to be due to increased illness in the older general population and conversely a healthier group with intellectual disabilities living to older ages compared with those who die younger. Although SMR was higher with increasing severity of intellectual disabilities, ability level was not retained within the multivariable model on time to death. The factors that were independently associated with increased risk of death, in order, were being percutaneous endoscopic gastrostomy/tube fed, Down syndrome, diabetes, having a lower respiratory tract infection at entry to the cohort, smoking, epilepsy, hearing impairment, total number of prescribed drugs, and age, whilst bowel incontinence had a reduced risk of death. Some of these predictors are similar to those reported in the general population, suggesting earlier mortality of adults with intellectual disabilities is largely accounted for by the higher rates of multi-morbidities that they experience compared with other people, and amenable deaths.[33]

For peer review only - http://bmjopen.bmj.com/site/about/guidelines.xhtml

Whilst accommodation type (not living with a family carer), ability level, not having day-time occupation, having spastic quadriplegia, visual impairment, constipation, ataxia/gait disorder, osteoporosis, hypertension, dysphagia, dyspnoea, gastro-oesophageal reflux disorder, total number of physical health disorders, not having impaired mobility, not having urinary incontinence, and not having autism, number of general practitioner consultations in the previous 12 months, total number of different types of health professionals providing care at the time of the health assessment, and antiepileptic drugs were related to time of death on univariate analyses, they were not retained in the multivariable model.

The majority of the adults with intellectual disabilities, with and without Down syndrome, died in an NHS hospital.

Comparison with previous literature

The overall SMR we report, higher SMR in women than men, and higher SMR at younger age groups is similar to the majority of previous reports. Most mortality studies with people with Down syndrome have been conducted with children. Previous reports of children and adults (combined) gave an SMR=5.5,[20] and for adults SMR=7.6,[9] compared with our finding for adults with Down syndrome of SMR=5.28. Recent systematic reviews reported people with intellectual disabilities on average died 20 years younger than other people, and people with Down syndrome died 28 years younger, although the majority of the Down syndrome studies were not recent.[1,2] In our study we found the gap between the age at death of people with intellectual disabilities with and without Down syndrome to be only 5.3 years, possibly reflecting the increasing lifespan of people with Down syndrome exceeding increases in lifespan for people with intellectual disabilities without Down syndrome. Notably, after “Down syndrome”, dementia was the most commonly reported underlying, and all contributing cause of death for the adults with Down syndrome, whereas studies in the past commented on congenital heart disease and respiratory causes.

For peer review only - http://bmjopen.bmj.com/site/about/guidelines.xhtml

For the cohort overall, respiratory infection and aspiration/reflux/choking were the most common all contributing causes of death. These conditions feature in previous studies on causes of death,[5,6,8,10,11] although there are inconsistencies between studies. By ICD-10 chapter, our study found the most common underlying causes of death were diseases of the respiratory system, then of the circulatory system, followed by neoplasms. Others reported the most common to be vascular,[10] circulatory,[5] heart disease, [17] and jointly circulatory and neoplasm.[19]

Previous research from other countries has highlighted that listing Down syndrome or intellectual disabilities as the underlying cause of death obscures actual causes of death for this population.[34] We therefore presented data on revised cause of death for the 21 people for whom it was listed as Down syndrome (as a sensitivity check), and highlight with interest that in this Scottish cohort no-one had intellectual disabilities listed as underlying cause of death. This may reflect different medical death certificate recording practices in Scotland compared to e.g. USA.

Studies that investigated avoidable deaths in adults with intellectual disabilities found them to be more common than in the general population, due to deaths that would have been amenable to good care. Avoidable deaths have been reported in 44.7% of deaths of people with intellectual disabilities in England (mostly amenable deaths – figure not reported),[6] and in 31% in Australia,[19] compared with our figure of 38.9%. Avoidable deaths that would have been amenable to good care have been reported to occur in 37% of deaths of people with intellectual disabilities in England.[5] Our figure is slightly lower at 29.8% but still more than double that found in the Scottish general population.[31] It should be noted that the ONS list of avoidable deaths was not designed specifically for

For peer review only - http://bmjopen.bmj.com/site/about/guidelines.xhtml

people with intellectual disabilities, and it may emphasise some causes less relevant, and omit others that might be highly relevant in this population.[5]

Strengths and limitations

The strengths of the study include the thorough methods of case ascertainment for intellectual disabilities at baseline with verification of intellectual disabilities and its severity, suggesting results are generalisable in other high income countries. Whist our Identification of the population will not have identified everyone with intellectual impairment (an IQ<70), in view of the multiple sources used, we believe it will have identified the adults with intellectual disabilities (IQ<70, plus need for support in daily activities, and onset in the developmental period). Additionally, there were detailed clinical assessments at baseline, and a longitudinal design. The size of the cohort and the duration of follow-up is also a strength, as is the successful record linkage for 94% of participants. Our study does have limitations, specifically that the study was only conducted in one region of Scotland, and the reliance upon death certificate data to obtain cause of death. Additionally, the characteristics and health of the participants was collected in 2002-2004. The health conditions we investigated tend to be long-standing or remitting/relapsing conditions, and psychotropic prescribing also once initiated tends to be long-standing in people with intellectual disabilities. However, it is possible that extent of neighbourhood deprivation, type of accommodation, employment, and civil status (though few marry) might have changed for some people between 2002-2004 and 2018; we have no further information to check this. There were no concerns regarding the proportional hazards assumption in the multivariable model. The linkage was also reliant on the accuracy of the CHI number as a sole source of linkage.

Implications

It is important to know the factors that are associated with risk of death, and the common causes of death in this population, as these then inform the actions needed to reduce the unacceptably high SMRs experienced by people with intellectual disabilities.

For peer review only - http://bmjopen.bmj.com/site/about/guidelines.xhtml

Awareness of these factors may provide a pathway to action to reduce the observed earlier mortality in adults with intellectual disabilities. It is not adequate to solely rely on the public health interventions available to everyone, even when they are accessible. Aspiration, reflux, and choking could, and should, be avoided by raising awareness of its consequences (death), and putting in place training on simple measures related to feeding, positioning, food consistency, and when to seek health advice from speech and language therapy, physiotherapy, nursing, and medical advice. Carers need to be aware of how the adults they care for express pain, so that conditions such as gastrointestinal ulcers are attended to, prior to the extreme point of perforation, and so treatable conditions such as constipation and urinary tract infections are managed before they lead to respiratory distress and sepsis. Quality of care is important; adults with intellectual disabilities need just as good care for their diabetes and epilepsy (and other conditions) as the rest of the population, with reasonable adjustments to address accessibility, and accessible smoking cessation programs.

Future research

Further research on larger samples is needed, particularly with regards to replicating and extending our findings on the factors that are associated with risk of death, and any sex differences in them, so that practitioners can focus on actions to improve the life expectancy of adults with intellectual disabilities, with and without Down syndrome.

References

1. O'Leary L, Hughes-McCormack L, Cooper S-A. Early death and causes of death of people with intellectual disabilities: A systematic review. *J Appl Res Intellect Disabil* 2017;31:325-42.
2. O'Leary L, Hughes-McCormack L, Cooper S-A. Life expectancy and causes of death of people with Down syndrome: a systematic review. *J Appl Res Intellect Disabil* 2018;31:687-708.

For peer review only - http://bmjopen.bmj.com/site/about/guidelines.xhtml

3. Cooper S-A, Hughes-McCormack L, Greenlaw N, *et al.* Management and prevalence of long-term conditions in primary health care for adults with intellectual disabilities compared with the general population: a population-based cohort study. *J Appl Res Intellect Disabil* 2017;31(S1):68–81. 10.1111/jar.12386
4. Heslop P, Blair PS, Fleming P, *et al.* The Confidential Inquiry into premature deaths of people with intellectual disabilities in the UK: a population-based study. *Lancet* 2014;383:889–95.
5. Hosking FJ, Carey IM, Shah SM, *et al.* Mortality among adults with intellectual disability in England: Comparisons with the general population. *Am J Public Healt,* 2016;106:1483–90. ERRATUM: *Am J Public Health* 2016;106:e11–e12. doi: 10.2105/AJPH.2016.303240e
6. Glover G, Williams R, Heslop P, *et al.* Mortality in people with intellectual disabilities in England. *J Intellect Disabil Res* 2017;61:62-74.
7. Fosgren L, Edvinsson SO, Nystrom L, *et al.* Influence of epilepsy on mortality in mental retardation: an epidemiological study. *Epilepsia 1996;*37:956-63.
8. Durvasula S, Beange H, Baker W. Mortality of people with intellectual disability in northern Sydney. *J Intellect Development Disabil* 2002; 27:255-64.
9. Tyrer F, Smith LK, McGrother CW. Mortality in adults with moderate to profound intellectual disability: a population-based study. *J Intellectl Disabil Res* 2007;51:520–7.
10. Patja K, Mölsä P, Iivanainen M. Cause-specific mortality of people with intellectual disability in a population-based, 35-year follow-up study. *J Intellect Disabil Res* 201;45:30–40.
11. Tyrer F, McGrother C. Cause-specific mortality and death certificate reporting in adults with moderate to profound intellectual disability. *J Intellect Disabil Res* 2009;53:898–904.
12. Ouellette-Kuntz H, Shooshtari S, Balogh R, *et al.* Understanding information about mortality among people with intellectual and developmental disabilities in Canada. *J Appl Res Intellect Disabil* 2015;28:423–35.

For peer review only - http://bmjopen.bmj.com/site/about/guidelines.xhtml

13. Florio T, Trollor J. Mortality among a cohort of persons with an intellectual disability in New South Wales, Australia. *J Appl Res Intellect Disabil* 2015;28:383–93.
14. McCarron M, Carroll R, Kelly C, *et al.* Mortality rates in the general Irish population compared to those with an intellectual disability from 2003 to 2012. *J Appl Res Intellect Disabil* 2015;28:406–13.
15. Heslop P, Glover G. Mortality of people with intellectual disabilties in England: a comparison of data from existing sources. *J Appl Res Intellect Disabil* 2015;28:414-22.
16. Lauer E, McCallion P. Mortality of people with intellectual and developmental disabilities from select US state disability service systems and medical claims data. *J Appl Res Intellect Disabil* 2015;28:394-405.
17. Arvio M, Salokivi T, Tiitinen A, *et al.* Mortality in individuals with intellectual disabilities in Finland. *Brain Behavior* 2016;6:e00431.
18. Lauer E. 2012 & 2013 Mortality Report, Massachusetts. 2016; https://shriver.umassmed.edu/sites/shriver.umassmed.edu/files/2012-13%20DDS%20Mortality%20Report%20Final_v2.pdf [accessed 9.7.19].
19. Troller J, Srasuebkul P, Xu H, *et al.* Cause of death and potentially avoidable deaths in Australian adults with intellectual disability using retrospective linked data. *BMJOpen* 2017;7:e013489
20. Day SM, Strauss DJ, Shavelle RM, Reynolds RJ. Mortality and causes of death in persons with Down syndrome in California. *Developmental Med Child Neurology* 2005;47:171-6.
21. Kinnear D, Morrison J, Allan L, *et al.* The prevalence of multi-morbidity in a cohort of adults with intellectual disabilities, with and without Down syndrome. *BMJOpen* 2018; 0:e018292
22. World Health Organisation. *International Statistical Classification of Diseases and Related Health Problems, 10th Revision.* Geneva: World Health Organisation, 1992.

For peer review only - http://bmjopen.bmj.com/site/about/guidelines.xhtml

23. World Health Organisation. *The ICD-10 Classification of Mental and Behavioural Disorders: Diagnostic Criteria for Research.* Geneva: World Health organisation, 1993.

24. American Psychiatric Association. *Diagnostic and Statistical Manual of Mental Disorders, Fourth Edition Revised.* Washington, DC: American Psychiatric Association, 2000.

25. Royal College of Psychiatrists. *DC-LD [Diagnostic Criteria for Psychiatric Disorders for Use with Adults with Learning Disabilities / Mental Retardation],* London: Gaskell Press, 2001.

26. Cooper S-A, Smiley E, Morrison J, *et al.* Prevalence of and associations with mental ill-health in adults with intellectual disabilities. *Br J Psychiatry 2007;*190:27-35**.**

27. Finlayson J, Jackson A, Cooper S-A, *et al.* Understanding predictors of low physical exercise in adults with intellectual disabilities. *J Appl Res Intellect Disabil* 2009;22:236-47.

28. National Records of Scotland Vital Events Reference Tables 2010. Section 5: Deaths https://www.nrscotland.gov.uk/statistics-and-data/statistics/statistics-by-theme/vital-events/general-publications/vital-events-reference-tables/archive/2010/section-5-deaths

29. National Records of Scotland. Mid-2010 Population Estimates Scotland Population estimates by sex, age and administrative area. https://www.nrscotland.gov.uk/files/statistics/population-estimates/mid-2010/mid-year-pop-est-2010.pdf

30. Office for National Statistics Avoidable Mortality in England and Wales, 2013, Newport, South Wales. 2015. https://www.ons.gov.uk/peoplepopulationandcommunity/healthandsocialcare/causesofdeath/bulletins/avoidablemortalityinenglandandwales/2015

31. National Records of Scotland. Avoidable mortality. https://www.nrscotland.gov.uk/statistics-and-data/statistics/statistics-by-theme/vital-events/deaths/avoidable-mortality

For peer review only - http://bmjopen.bmj.com/site/about/guidelines.xhtml

32. Public Health England. Health profile for England. 2018 https://www.gov.uk/government/publications/health-profile-for-england-2018/chapter-2-trends-in-mortality

33. Cooper S-A, McLean G, Guthrie B, *et al.* Multiple physical and mental health comorbidity in adults with intellectual disabilities: population-based cross-sectional analysis. *BMC Family Practice*, 2015;DOI:10.1186/s12875-015-0329-3

34. Landes SD, Stevens JD, Turk MA. The obscuring effect of coding developmental disability as the underlying cause of death on mortality trends for adults with developmental disability: a cross-sectional study utilizing U.S. mortality data from 2012 to 2016. *BMJ Open*, 2019. 9:e026614

Funding

This work was supported by the UK Medical Research Council, grant number: MC_PC_17217), and the Scottish Government via the Scottish Learning Disabilities Observatory.

The study sponsor and funders had no role in the study design; in the collection, analysis, and interpretation of data; in the writing of the report; and in the decision to submit the article for publication.

The researchers are independent from the funders.

Competing interests

The authors declare no competing interests.

Author's contributions

For peer review only - http://bmjopen.bmj.com/site/about/guidelines.xhtml

S-AC is principle investigator, she conceived and managed the project, interpreted data, and wrote the first draft of the manuscript. LA contributed to the conception of the project, and project management. NG designed and supervised the statistical analysis, and contributed to data interpretation and drafting of the manuscript. PMcS implemented and refined the statistical analysis, and contributed to data interpretation, and drafting of the manuscript. AJ implemented and refined the statistical analysis, and contributed to data interpretation. AH contributed to data linkage and interpretation, and drafting of the manuscript. CMcC provided expertise on data linkage and methods, and drafting of the manuscript. DK contributed to data interpretation and drafting of the manuscript. CM contributed to data interpretation, and drafting of the manuscript. All approved the final version of the manuscript. S-AC is the study guarantor.

Data sharing

Data is available via NHS GG&C Safe Haven upon application.

Acknowledgments

We thank the NHS Greater Glasgow and Clyde learning disabilities primary care liaison team for their contribution to the study, and the participants with intellectual disabilities and their carers.

For peer review only - http://bmjopen.bmj.com/site/about/guidelines.xhtml

Table 1. Cohort characteristics at time of the health assessment, summarised overall and by death status during the follow-up period

Variable	Statistics / Groups	All participants (N=961)	Deceased participants (N=294)	Alive participants (N=667)
Age (years)	Mean (SD)	44.1 (14.6)	52.4 (13.6)	40.5 (13.6)
	Min, max	16, 83	18, 83	16, 77
Age group	16-25 years	127 (13.2%)	10 (3.4%)	117 (17.5%)
	26-35 years	153 (15.9%)	26 (8.8%)	127 (19.0%)
	36-45 years	246 (25.6%)	49 (16.7%)	197 (29.5)
	46-55 years	205 (21.3%)	85 (28.8%)	120 (18.0%)
	>55 years	230 (23.9%)	124 (42.0%)	106 (15.9%)
Sex	Male	525 (54.6%)	154 (52.4%)	371 (55.6%)
	Female	436 (45.3%)	140 (47.5%)	296 (44.4%)
Ability level	Mild ID	382 (39.7%)	92 (31.2%)	290 (43.5%)
	Moderate ID	236 (24.5%)	73 (24.7%)	163 (24.4%)
	Severe ID	180 (18.7%)	67 (22.7%)	113 (16.9%)
	Profound ID	163 (17.0%)	62 (21.1%)	101 (15.1%)
Accommodation type	Family carer	374 (38.9%)	70 (23.8%)	304 (45.6%)
	Independent	93 (9.7%)	36 (12.2%)	57 (8.5%)
	Paid support	435 (45.2%)	161 (54.6%)	274 (41.1%)
	Congregate care	59 (6.1%)	27 (9.2%)	32 (4.8%)
Down syndrome	No	782 (81.4%)	230 (78.2%)	552 (82.8%)
	Yes	179 (18.6%)	64 (21.7%)	115 (17.2%)

ID=intellectual disabilities; SD=standard deviation

For peer review only

Table 2. Standardised mortality ratios

Variable	Groups	SMR (95% CI)
All participants	-	2.24 (1.99, 2.50)
Age group*	15-25 years	18.73 (0.37, 37.09)
	26-35 years	4.21 (1.29, 7.13)
	36-45 years	3.86 (2.28, 5.44)
	46-55 years	3.77 (2.90, 4.74)
	>55 years	1.86 (1.60, 2.12)
Sex	Male	1.69 (1.42, 1.95)
	Female	3.48 (2.90, 4.06)
Ability level	Mild ID	1.60 (1.27, 1.92)
	Moderate ID	2.10 (1.62, 2.58)
	Severe ID	2.78 (2.11, 3.44)
	Profound ID	4.14 (3.11, 5.17)
Down syndrome	No	1.93 (1.68, 2.18)
	Yes	5.28 (3.98, 6.57)
Underlying causes of death grouped by ICD-10 chapter**	Congenital malformations, deformations and chromosomal abnormalities	17.26 (10.75, 23.78)
	Diseases of the blood and blood-forming organs and certain disorders involving the immune mechanism	7.50 (-7.20, 22.20)
	Diseases of the circulatory system	5.55 (4.01, 7.09)
	Diseases of the digestive system	16.13 (8.23, 24.04)
	Diseases of the genitourinary system	3.65 (0.73, 6.57)
	Diseases of the musculoskeletal system and connective tissue	5.40 (-0.71, 11.52)
	Diseases of the nervous system	7.73 (5.13, 10.32)
	Diseases of the respiratory system	6.78 (5.02, 8.54)

For peer review only - http://bmjopen.bmj.com/site/about/guidelines.xhtml

	Diseases of the skin and subcutaneous tissue	2.75 (-2.64, 8.15)
	Endocrine, nutritional and metabolic diseases	3.43 (1.05, 5.81)
	External causes of morbidity and mobility	11.08 (3.40, 18.76)
	Infectious and parasitic diseases	8.93 (1.78, 16.07)
	Mental and behavioural disorders	12.64 (3.27, 22.00)
	Neoplasms	6.31 (4.19, 8.43)
	Symptoms, signs and abnormal clinical and laboratory findings, not elsewhere classified	19.51 (0.39, 38.63)

CI=confidence intervals; ID=intellectual disabilities; SMR=standardised mortality ratios

*Data used for comparison with General Population (GG&C Health Board) provides data in 5 year age bands therefore 15+. Data on adults with ID are 16+

** Negative Lower CI and wide CIs indicate low number of observed deaths in study population

For peer review only

Table 3. Underlying causes of death grouped by ICD-10 chapter, where cause of death is known

ICD-10 chapter	Participants with Down syndrome (N=57)	Participants without Down syndrome (N=205)
Certain infectious and parasitic diseases	5 (8.8%)	<5
Neoplasms	<5	33 (16.1%)
Diseases of the blood and blood-forming organs and certain disorders involving the immune mechanism	<5	<5
Endocrine, nutritional and metabolic diseases	<5	8 (3.9%)
Mental and behavioural disorders	5 (8.8%)	<5
Diseases of the nervous system	7 (12.3%)	27 (13.2%)
Diseases of the eye and adnexa	<5	<5
Diseases of the ear and mastoid process	<5	<5
Diseases of the circulatory system	8 (14.0%)	42 (20.5%)
Diseases of the respiratory system	8 (14.0%)	49 (23.9%)
Diseases of the digestive system	<5	16 (7.8%)
Diseases of the skin and subcutaneous tissue	<5	<5
Diseases of the musculoskeletal system and connective tissue	<5	<5
Diseases of the genitourinary system	<5	5 (2.4%)
Pregnancy, childbirth and the puerperium	<5	<5
Certain conditions originating in the perinatal period	<5	<5
Congenital malformations, deformations and chromosomal abnormalities	21 (36.8%)	6 (2.9%)
Symptoms, signs and abnormal clinical and laboratory findings, not elsewhere classified	<5	<5
External causes of morbidity and mortality	<5	7 (3.4%)
All deaths	57 (100%)	205 (100%)

ICD-10=International Statistical Classification of Diseases and Related Health Problems, 10th Revision

For peer review only

For peer review only - http://bmjopen.bmj.com/site/about/guidelines.xhtml

Table 4. Underlying causes of death grouped by specific individual causes or related groups of causes, where cause of death is known

Causes	Participants with Down syndrome (N=57)	Participants without Down syndrome (N=205)
Aspiration/reflux/choking	<5	22 (10.8%)
Respiratory infection	<5	21 (10.3%)
Down syndrome	21 (36.8%)	<5
Other malignancy	<5	19 (9.3%)
Other condition	<5	17 (8.3%)
Epilepsies	<5	13 (6.4%)
Acute myocardial infarction	<5	13 (6.4%)
Gastro-intestinal malignancy	<5	12 (5.9%)
Stroke	<5	11 (5.4%)
Other cardiovascular disease	<5	11 (5.4%)
Other respiratory condition	<5	9 (4.4%)
Other infection	5 (8.8%)	6 (2.9%)
Cerebral palsy	<5	11 (5.4%)
Dementia	9 (15.8%)	<5
Other gastrointestinal disorders	<5	8 (3.9%)
Ulcer/gastrointestinal perforation	<5	7 (3.4%)
Diabetes	<5	7 (3.4%)
Other congenital condition	<5	6 (2.9%)
Other ischaemic heart condition	<5	6 (2.9)
Mental health	<5	<5
Other neurological conditions	<5	<5
Renal failure	<5	<5
All deaths	57 (100%)	205 (100%)

For peer review only

For peer review only - http://bmjopen.bmj.com/site/about/guidelines.xhtml

Table 5. All contributing causes of death grouped by specific individual causes or related groups of causes, where cause of death is known

Causes	Participants with Down syndrome (N=57)	Participants without Down syndrome (N=205)
Respiratory infection	22 (38.6%)	49 (23.9%)
Aspiration/reflux/choking	11 (19.3%)	41 (20.0%)
Down syndrome	43 (75.4%)	<5
Other condition	8 (14.0%)	33 (16.1%)
Other cardiovascular disease	8 (14.0%)	30 (14.6%)
Other respiratory conditions	<5	31 (15.1%)
Other infection	9 (15.8%)	24 (11.7%)
Intellectual disabilities	<5	31 (15.1%)
Epilepsies	8 (14.0%)	24 (11.7%)
Dementia	24 (42.1%)	<5
Other neoplasms	<5	23 (11.2%)
Cerebral palsy	<5	24 (11.7%)
Acute myocardial infarction	5 (8.8%)	19 (9.3%)
Other gastrointestinal disorders	<5	18 (8.8%)
Diabetes	<5	19 (9.3%)
Other ischaemic heart disease	<5	19 (9.3%)
Renal failure	<5	16 (7.8%)
Stroke	<5	17 (8.3%)
Other congenital condition	<5	15 (7.3%)
Gastrointestinal malignant neoplasm	<5	12 (5.9%)
Ulcer/gastrointestinal perforation	<5	10 (4.9%)
Mental health	<5	10 (4.9%)
Other neurological condition	<5	8 (3.9%)
Heart failure	<5	7 (3.4%)
Injuries and accidents	<5	8 (3.9%)
Medical/surgical complications	<5	<5
Secondary malignancies	<5	<5
Thyroid disorders	<5	<5
Metabolic disorder	<5	<5
All deaths	57 (100%)	205 (100%)

For peer review only

For peer review only - http://bmjopen.bmj.com/site/about/guidelines.xhtml

Table 6. Multivariable model results for the outcome time to death

Variable		Hazard ratio	95% CI	p-value
Age at time of health assessment		1.056	1.046, 1.066	<0.0001
Smoker	No	1	-	
	Yes	1.531	1.1011, 2.128	0.0112
Down syndrome	No	1	-	
	Yes	2.440	1.787, 3.332	<0.0001
Epilepsy	No	1	-	
	Yes	1.511	1.173, 1.946	0.0014
Hearing impairment	No	1	-	
	Yes	1.320	1.030, 1.692	0.0284
Bowel incontinence	No	1	-	
	Yes	0.490	0.376, 0.640	<0.0001
Diabetes	No	1	-	
	Yes	2.346	1.553, 3.542	<0.0001
PEG/tube fed	No	1	-	
	Yes	2.346	1.135, 5.989	0.00240
Lower respiratory track infection	No	1	-	
	Yes	1.782	1.315, 2.415	0.0002
Total number of prescribed drugs		1.066	1.016, 1.118	0.0085

CI=confidence interval; PEG=percutaneous endoscopic gastrostomy

For peer review only

Supplementary table 1. Previously reported standardised mortality ratios, causes, and risks for death

Author	Country	SMR (95% confidence interval)	Number of deaths	Causes of death and risk factors for death
Forsgren et al (1996)[7]	Sweden	4.2 (3.3, 5.3) at 20-59y; 1.1 (0.9, 1.5) at 60+y<br>*Without epilepsy:*<br>3.8 (2.8, 5.0) at 20-59y; 1.1 (0.8, 1.5) at 60+y<br>*With epilepsy:*<br>5.0 (2.9, 8.7) at 20-59y; 2.4 (0.9, 6.1) at 60+y<br>*With epilepsy and cerebral palsy:*<br>8.0 (4.1, 15.7) at 20-59y; 0.9 (0.1, 6.6) at 60+y<br>*M:* 1.6 (1.2, 2.0) at 0-60+y<br>*F:* 2.6 (2.0, 3.3) at 0-60+y<br>*Mild ID:* 1.8 (1.1, 2.7) at 0-60+y<br>*Moderate ID:* 1.5 (1.1, 2.0) at 0-60+y<br>*Severe ID:* 2.0 (1.5, 2.6) at 0-60+y<br>*Profound ID:* 8.1 (5.6, 11.7) at 0-60+y	124 at 0-60+y; 112 at 20-60+y	*Underlying cause at 0-60+y:*<br>Congenital anomalies: SMR=46.3 (32.9, 65.0)<br>Nervous system: SMR=9.7 (5.5, 17.0)<br>Mental disorder: SMR=4.0 (1.9, 8.4)<br>Respiratory: SMR=3.3 (2.0, 5.5)<br>Circulatory: SMR=2.1 (1.6, 2.7)<br>Violent death: SMR=1.4 (0.6, 2.8)<br>Neoplasm: SMR=0.9 (0.6, 1.6)
Durvasula & Beange (2002)[8]	Australia	4.9 (3.4, 6.4) at 10-59y<br>*M:* 4.1 (2.4, 5.9) at 10-59y<br>*F:* 6.2 (3.3, 9.1) at 10-59y	40 at 10-59y; 31 at 20-59y	*Underlying cause at 10-59y:*<br>Respiratory: 35% (pneumonia, aspiration)<br>External causes: 20%<br>Neoplasm: 17.5%<br>Heart disease: 15% (congenital heart disease 50%)<br>Gastrointestinal: 7.5% (ischaemic bowel, perforated peptic ulcer, post-operative peritonitis)<br>Seizure: 5%
Tyrer et al (2007)[9]	England	3.24 (2.93, 3.56) at 20-70+y<br>*M:* 2.86 (2.50, 3.26) at 20-70+y<br>*F:* 3.63 (3.12, 4.20) at 20-70+y<br>1.51 (1.23, 1.83) to 11.50 (8.14, 15.78) at 20-70+y<br>*M:* 1.39 (1.03, 1.82) to 8.83 (5.60, 13.25) at 20-70+y<br>*F:* 1.60 (1.18, 2.12) to 17.22 (9.64, 28.4) at 20-70+y<br>*With Down syndrome:* 7.60 at 20-70+y<br>*Without Down syndrome:* 2.70 at 20-70+y	409 at 20-70+y	Not reported
Patja et al (2008)[10]	Finland	*M:* 2.2 at 20-39y, 1.0 at 40-59y, 1.0 at 60+y<br>*F:* 1.4 at 20-39y, 1.1 at 40-49y, 1.0 at 60+y<br>*Mild ID:*<br>M: 1.6 at 20-39y, 1.0 at 40-59y, 1.0 at 60+y<br>F: 1.2 at 20-39y, 1.1 at 40-49y, 1.0 at 60+y	1,046 at 20-97y	*Underlying cause at 2-97y:*<br>Vascular: 36% (cardiac infarct 33%, cerebral infarct 33%, congenital heart disease 18%, pulmonary infarct 6%)<br>Respiratory: 22% (pneumonia 83%, COPD 11%)

For peer review only

Rates, causes, place, and predictors of mortality in adults with intellectual disabilities with and without Down syndrome: cohort study with record linkage

		Moderate ID:<br>M: 2.3 at 20-39y, 1.1 at 40-59y, 1.0 at 60+y<br>F: 1.5 at 20-39y, 1.1 at 40-49y, 1.0 at 60+y<br>*Severe ID:*<br>M: 2.6 at 20-39y, 1.2 at 40-59y, 1.0 at 60+y<br>F: 1.6 at 20-39y, 1.0 at 40-49y, 1.0 at 60+y<br>*Profound ID:*<br>M: 2.1 at 20-39y, 1.1 at 40-59y, 1.0 at 60+y<br>F: 1.3 at 20-39y, 1.2 at 40-49y, 1.0 at 60+y		Neoplasm: 11% (digestive 44%, respiratory 15%, urogenital, 12%)<br>Digestive: 7% (intestinal obstruction 25%, ulcer perforation 13%)<br>Accidents and poisonings: 7% (commonest was fatal fracture, then drowning)<br>Vascular, neoplasm, and accident causes were less common than sex-age-matched general population; Digestive were 2.5 times, Respiratory 2.6-5.8 times more common
Tyrer & McGother (2009)[11]	England	2.77 (2.53, 3.03) at 20+y<br>*M:* 2.28 (2.02, 2.56) at 20+y<br>*F:* 3.24 (2.83, 3.69) at 20+y	503 at 20+y	*Underlying cause at 20+y:*<br>Pneumonia: 13.1%, SMR=6.47 (5.00, 8.23)<br>Nervous system: 13.1%, SMR=16.30 (12.61, 20.74)<br>Other respiratory: 12.9%, SMR=4.64 (3.58, 5.91)<br>Ischaemic heart disease: 11.5%, SMR=1.49 (1.13, 1.92)<br>Neoplasm: 9.3%<br>Congenital anomalies: 9.1%, SMR=85.60 (62.67, 114.18)<br>Cerebrovascular disease: 7.8%, SMR=2.40 (1.71. 3.28)
Oullette-Kuntz et al (2015)[12]	Canada	2.5 (2.1, 2.9) at 0-60+y<br>*M:* 2.1 (1.7, 2.6) at 0-60+y<br>*F:* 3.0 (2.4, 3.8) at 0-60+y<br>*M:* 1.7 (1.3, 2.3) to 3.4 (2.3, 4.7) at 20-60+y<br>*F:* 2.1 (1.4, 2.9) to 6.1 (4.1, 8.6) at 20-60+y	172 at 0-60+y; 158 at 20-60+y	*Risk factors for death:*<br>Age, Down syndrome (OR=1.76 at 20-39y; OR=1.69 at 40-59y: OR=22.34 at 60+y), cerebral palsy (OR=2.39 at 20-39y; OR=0.93 at 40-59y: OR=0.50 at 60+y), blindness/low vision (OR not given), technological dependance/medical fragility (OR=11.96 at 20-39y; OR=7.28 at 40-59y: OR=3.42 at 60+y), wheelchair dependence (OR=5.96 at 20-39y; OR=2.89 at 40-59y: OR=2.56 at 60+y), mobility impairment without wheelchair dependence (OR not given), epilepsy (OR=1.83 at 20-39y; OR=1.80 at 40-59y: OR=1.09 at 60+y)
Florio & Troller (2015)[13]	Australia	2.48 (2.32, 2.64) at 0-85+y<br>3.15 (2.94, 3.38) at 5-69y<br>*M:* 2.52 (2.29, 2.77) at 5-69y<br>*F:* 4.26 (3.83, 4.74) at 5-69y	953 at 0-85+y; 831 at 15+y	Not reported
McCarron et al (2015)[14]	Republic of Ireland	3.85 (3.70, 4.00) at 0-80+y<br>*M:* 3.09 (2.93, 3.25) at 0-80+y<br>*F:* 4.90 (4.63, 5.17) at 0-80+y<br>2.71 (2.41, 3.04) to 6.09 (5.29, 6.96) at 20-80y<br>*M:* 2.50 (2.18, 2.86) to 4.50 (3.69, 5.44) at 20-80y<br>*F:* 2.71 (2.32, 3.14) to 10.07 (8.99, 13.10) at 20-80y	2,666 at 0-80+y; 2,394 at 20-80+y	Not reported

Rates, causes, place, and predictors of mortality in adults with intellectual disabilities with and without Down syndrome: cohort study with record linkage

Heslop & Glover (2015)[15]	England	Median 2.13 (interquartile range 1.09, 2.83) across geographic areas at 18-65+y	18-65+y	Not reported
Lauer & McCallion (2015)[16]	USA	*Intellectual and developmental disabilities*:*<br>1.19 at all ages, 2011<br>1.49 at 18+y, 2009	120,913 in 2009 at 18+y, 140,104 in 2011 at all ages	Not reported
Arvio et al (2016)[17]	Finland	*Mild ID:*<br>2.28 (2.18, 2.39) at 0-60+y<br>1.99 (1.85, 2.13) to 2.77 (2.36, 3.23) at 15-60+y<br>*M:* 2.01 (1.88, 2.14) at 0-60+y<br>*F:* 2.80 (2.60, 3.01) at 0-60+y<br>*Severe ID:*<br>3.41 (3.30, 3.52) at 0-60+y<br>2.07 (1.96, 2.19) to 8.77 (7.77, 9.87) at 15-60+y<br>*M:* 2.59 (2.48, 2.72) at 0-60+y<br>*F:* 5.24 (4.99, 5.50) at 0-60+y	5,171 at 0-60+y; 5,053 at 15-60y	Not reported
Hosking et al (2016)[5]	England	HR=3.62 (3.33, 3.93) at 18-84y<br>*M:* HR=3.30 (2.96, 3.68) at 18-84y<br>*F:* HR=4.10 (3.61, 4.66) at 18-84y<br>*With Down syndrome:* HR=9.21 (7.22, 11.76)<br>*Without Down syndrome:* HR=3.19 (2.92, 3.49)<br>*With epilepsy:* HR=6.04 (5.04, 7.24)<br>*Without epilepsy:* HR=3.18 (2.90, 3.50)<br>*With high level of support needs:* HR=4.77 (4.08, 5.59)<br>*Without high level of support needs:* HR=3.28 (2.98, 3.62)<br>*With autism:* HR=2.39 (1.45, 3.96)<br>*Without autism:* HR=3.66 (3.37, 3.98)<br>*In communal/shared homes:* HR=4.99 (4.36, 5.73)<br>*Not in communal/shared homes:* HR=3.05 (2.74, 3.30)	656 at 18-84y	*Underlying cause at 18-84y:*<br>Circulatory: 21.6%, HR=3.05 (2.56, 3.64)<br>Respiratory: 18.8% (pneumonia and aspiration pneumonia), HR=6.68 (5.38, 8.29)<br>Neoplasm: 14.9%, HR=1.44 (1.18, 1.76)<br>Nervous system: 11.6%, HR=13.79 (9.70, 19.62)<br>Digestive: 7.0%, HR=4.02 (2.92, 5.54)<br>Congenital anomalies: 6.9%, HR could not be estimated<br>Mental disorders: 5.3%, HR=7.99 (5.19, 12.31)<br>External causes: 4.1%, HR=1.85 (1.26, 2.71)<br>Genitourinary: 3.5%, HR=10.89 (6.09, 19.47)<br>Endocrine, nutritional, and metabolic: 2.0%, HR=5.38 (2.79, 10.07)<br>*Down syndrome:* Respiratory: 20.3% (or 42.4% if "Down syndrome" is excluded as an underlying cause of death)<br>*Avoidable deaths:*<br>37% amenable (23% controls), 19% preventable (40% controls)

For peer review only

Lauer (2016)[18]	USA	Not reported	438 in 2012, 409 in 2013, at 18+y	*Major cause of death, 2012, 2013*<br>Heart disease: 16.0%, 13.7%<br>Neoplasm: 13.7%, 13.4%<br>Alzheimer disease: 13.0%-12.2% (48% in Down syndrome)<br>Aspiration pneumonia: 9.4%, 8.6%<br>Septicaemia: 10.0%, 8.6%<br>Chronic lower respiratory diseases: 4.6%, 6.6%<br>Unintentional injury: 4.8%, 3.2%
Troller et al (2017)[19]	Australia	1.3 (1.2, 1.5) at 20+y<br>4.0 (3.1, 5.2) at 20-44y<br>2.3 (2.0, 2.7) at 45-64y<br>1.0 (0.8, 1.20 at 65+y<br>*M:*1.4 (1.1, 1.6) at 20+y<br>*F:* 1.3 (1.1, 1.6) at 20+y	732 at 20-65+y	*Underlying cause at 20-65+y:*<br>Circulatory: 18%<br>Neoplasm: 18%<br>Nervous: 16%<br>Respiratory: 12%<br>Congenital anomaly: 11%<br>Injury and poisoning: 6%<br>Digestive: 5%<br>*Avoidable deaths:* 31%
Glover et al (2017)[6]	England	3.18 (2.94, 3.43) at 0-99y<br>*M:* 3.03 (2.73, 3.35) at 0-99y<br>*F:* 3.40 (3.02, 3.81) at 0-99y<br>1.6 (1.2, 2.1) to 7.8 (5.4, 11.1) at 18-99y<br>*M:* 1.5 (0.9, 2.2) to 6.6 (4.0, 10.1) at 18-99y<br>*F:* 1.7 (1.1, 2.4) to 11.6 (6.0, 20.2) at 18-99y	664 at 0-99y	*Underlying cause at 0-99y:*<br>Circulatory: 22.9% (ischaemic heart disease 37.5%, cerebrovascular 25.7%, thrombophlebitis 6.6%, cardiomyopathy 5.9%, PE 3.9%), SMR=2.8 (2.4, 3.3)<br>Respiratory: 17.2% (pneumonia 50.0%, pneumonitis 21.0%), SMR=4.9 (4.0, 5.9)<br>Neoplasm: 3.1% (digestive 36.8%, respiratory 13.8%, female genital tract 10.3%, lymphoid and haematopoietic 10.3%), SMR=1.1 (0.9, 1.4)<br>Nervous: 12.8%, SMR=9.8 (7.8, 12.1)<br>Congenital anomalies: 8.4%, SMR=72.9 (55.1, 94.7)<br>Digestive: 7.8%, SMR=4.0 (3.0, 5.2)<br>No ICD10 chapters had fewer than expected deaths<br>Other common single causes: dementia 33/664, epilepsy 26/664, cerebral palsy 23/664<br>*Avoidable deaths:*<br>44.7% (41.0%, 48.5%), mostly amenable<br>M: 50.9% (45.9%, 56.0%); F: 36.9% (31.5%, 42.5%)

COPD=chronic obstructive pulmonary disease; HR=hazard ratio; ID=intellectual disabilities; OR=odds ratio; PE=pulmonary embolism; SMR=standardised mortality ratio; y=years

*Includes some individuals with IQ>70

Rates, causes, place, and predictors of mortality in adults with intellectual disabilities with and without Down syndrome: cohort study with record linkage

For peer review only

Rates, causes, place, and predictors of mortality in adults with intellectual disabilities with and without Down syndrome: cohort study with record linkage

Supplementary table 2. Groupings of related causes of deaths

Infectious diseases	ICD10
**Infection**	
ENTEROCOLITIS DUE TO CLOSTRIDIUM DIFFICILE	A047
SEPSIS DUE TO STAPHYLOCOCCUS AUREUS	A410
SEPSIS, UNSPECIFIED	A419
BACTERIAL INFECTION, UNSPECIFIED	A499
SUBACUTE SCLEROSING PANENCEPHALITIS	A811
CHRONIC VIRAL HEPATITIS B WITHOUT DELTA-AGENT	B181
PULMONARY CANDIDIASIS	B371
NECROTISING FASCIITIS	M726
URINARY TRACT INFECTION, SITE NOT SPECIFIED	N390
**Neoplasms**	
**Gastrointestinal malignant neoplasms**	
MALIGNANT NEOPLASM OF PAROTID GLAND	C07
MALIGNANT NEOPLASM, OESOPHAGUS, UNSPECIFIED	C159
MALIGNANT NEOPLASM, STOMACH, UNSPECIFIED	C169
MALIGNANT NEOPLASM, CAECUM	C180
MALIGNANT NEOPLASM, SIGMOID COLON	C187
MALIGNANT NEOPLASM, COLON, UNSPECIFIED	C189
INTRAHEPATIC BILE DUCT CARCINOMA	C221
NEOPLASM OF UNCERTAIN OR UNKNOWN BEHAVIOUR, OTHER DIGESTIVE ORGANS	D377
**Other neoplasms**	
MALIGNANT NEOPLASM, LOWER LOBE, BRONCHUS OR LUNG	C343
MALIGNANT NEOPLASM, BRONCHUS OR LUNG, UNSPECIFIED	C349
MALIGNANT NEOPLASM, BREAST, UNSPECIFIED	C509
MALIGNANT NEOPLASM, ENDOMETRIUM	C541
MALIGNANT NEOPLASM OF OVARY	C56
MALIGNANT NEOPLASM, TESTIS, UNSPECIFIED	C629
MALIGNANT NEOPLASM, BLADDER, UNSPECIFIED	C679
MALIGNANT NEOPLASMS OF THYROID GLAND	C73
WALDENSTROM MACROGLOBULINAEMIA	C880
NON-HODGKIN'S LYMPHOMA, UNSPECIFIED	C859
MALIGNANT NEOPLASM OF UNSPECIFIED SITE	C80
NEOPLASM OF UNCERTAIN OR UNKNOWN BEHAVIOUR, TRACHEA, BRONCHUS AND LUNG	D381
SECONDARY MALIGNANT NEOPLASM OF LUNG	C780
SECONDARY MALIGNANT NEOPLASM OF LIVER AND INTRAHEPATIC BILE DUCT	C787
SECONDARY MALIGNANT NEOPLASM OF BRAIN AND CEREBRAL MENINGES	C793
SECONDARY MALIGNANT NEOPLASM OF OTHER SPECIFIED SITES	C798
**Endocrine and metabolic diseases**	
**Diabetes**	
INSULIN-DEPENDENT DIABETES MELLITUS WITHOUT COMPLICATIONS	E109
NON-INSULIN-DEPENDENT DIABETES MELLITUS WITH RENAL COMPLICATIONS	E112
NON-INSULIN-DEPENDENT DIABETES MELLITUS W. PERIPHERAL CIRC. COMPLICATIONS	E115
NON-INSULIN-DEPENDENT DIABETES MELLITUS WITHOUT COMPLICATIONS	E119
UNSPECIFIED DIABETES MELLITUS WITH RENAL COMPLICATIONS	E142

For peer review only

Rates, causes, place, and predictors of mortality in adults with intellectual disabilities with and without Down syndrome: cohort study with record linkage

UNSPECIFIED DIABETES MELLITUS WITHOUT COMPLICATIONS	E149
ABNORMAL GLUCOSE TOLERANCE TEST	R730
HYPERGLYCAEMIA, UNSPECIFIED	R739
**Metabolic disorders**	
OTHER HYPERPHENYLALANINAEMIAS	E701
DISORDERS OF PHOSPHORUS METABOLISM & PHOSPHATASES	E833
DISORDERS OF PLASMA-PROTEIN METABOLISM, NOT ELSEWHERE CLASSIFIED	E880
**Mental disorders**	
**Dementias**	
VASCULAR DEMENTIA, UNSPECIFIED	F019
UNSPECIFIED DEMENTIA	F03
ALZHEIMER'S DISEASE WITH LATE ONSET	G301
ALZHEIMER'S DISEASE, UNSPECIFIED	G309
**Mental health**	
MENTAL AND BEHAVIOURAL DISORDERS DUE TO ACUTE INTOXICATION WITH ALCOHOL	F100
MENTAL AND BEHAVIOURAL DISORDERS DUE TO ALCOHOL DEPENDENCE SYNDROME	F102
MENTAL AND BEHAVIOURAL DISORDERS DUE TO USE OF TOBACCO, UNSPECIFIED	F179
SCHIZOPHRENIA, UNSPECIFIED	F209
BIPOLAR AFFECTIVE DISORDER, UNSPECIFIED	F319
OTHER & UNSPEC SYMPTOMS & SIGNS INVOLVING COGNITIVE FUNCTIONS & AWARENESS	R418
INTENTIONAL SELF-HARM BY JUMPING FROM A HIGH PLACE	X80
**Intellectual disabilities**	
UNSPECIFIED MENTAL RETARDATION	F79
DEVELOPMENTAL DISORDER OF SCHOLASTIC SKILLS, UNSPECIFIED	F819
**Nervous system**	
**Epilepsies**	
GENERALIZED IDIOPATHIC EPILEPSY AND EPILEPTIC SYNDROMES	G403
EPILEPSY, UNSPECIFIED	G409
STATUS EPILEPTICUS, UNSPECIFIED	G419
MYOTONIC DISORDERS	G711
OTHER AND UNSPECIFIED CONVULSIONS	R568
**Cerebral palsy**	
SPASTIC QUADRAPLEGIC CEREBRAL PALSY	G800
SPASTIC HEMIPLEGIC CEREBRAL PALSY	G802
OTHER CEREBRAL PALSY	G808
CEREBRAL PALSY, UNSPECIFIED	G809
TETRAPLEGIA, UNSPECIFIED	G825
**Other neurological conditions**	
SEQUELAE OF INFLAMMATORY DISEASES OF CENTRAL NERVOUS SYSTEM	G09
PARKINSON'S DISEASE	G20
MYONEURAL DISORDER, UNSPECIFIED	G709
ENCEPHALITIS, MYELITIS AND ENCEPHALOMYELITIS, UNSPECIFIED	G049
ANOXIC BRAIN DAMAGE, NOT ELSEWHERE CLASSIFIED	G931
BLINDNESS, BINOCULAR	H540
OTHER DISORDERS OF NERVOUS SYSTEM, NOT ELSEWHERE CLASSIFIED	G98

For peer review only

Rates, causes, place, and predictors of mortality in adults with intellectual disabilities with and without Down syndrome: cohort study with record linkage

Circulatory system

Acute myocardial infarction

ACUTE MYOCARDIAL INFARCTION, UNSPECIFIED	I219
CARDIAC ARRECT, UNSPECIFIED	I469
**Other ischaemic heart disease**	
HYPERTENSIVE HEART DISEASE WITHOUT (CONGESTIVE) HEART FAILURE	I119
ACUTE ISCHAEMIC HEART DISEASE, UNSPECIFIED	I249
ATHEROSCLEROTIC HEART DISEASE	I251
CHRONIC ISCHAEMIC HEART DISEASE, UNSPECIFIED	I259
ATHEROSCLEROSIS OF AORTA	I700
GENERALIZED AND UNSPECIFIED ATHEROSCLEROSIS	I709
**Heart failure**	
HEART FAILURE, UNSPECIFIED	I509
LEFT VENTRICULAR FAILURE	I501
CONGESTIVE HEART FAILURE	I500
**Other cardiovascular disease**	
PULMONARY EMBOLISM WITHOUT MENTION OF ACUTE COR PULMONALE	I269
OTHER SPECIFIED PULMONARY HEART DISEASES	I278
PULMONARY HEART DISEASE, UNSPECIFIED	I279
AORTIC (VALVE) STENOSIS	I350
ATRIAL FIBRILLATION AND FLUTTER	I48
VENTRICULAR FIBRILLATION AND FLUTTER	I490
OTHER ILL-DEFINED HEART DISEASES	I518
PULMONARY OEDEMA	J81
CARDIOGENIC SHOCK	R570
PERIPHERAL VASCULAR DISEASE, UNSPECIFIED	I739
PHLEBITIS AND THROMBOPHLEBITIS OF OTHER DEEP VESSELS OF LOWER EXTREMITIES	I802
EMBOLISM AND THROMBOSIS OF OTHER SPECIFIED VEINS	I828
ACUTE AND SUBACUTE INFECTIVE ENDOCARDITIS	I330
ACUTE ENDOCARDITIS, UNSPECIFIED	I339
ENDOCARDITIS, VALVE UNSPECIFIED	I38
DILATED CARDIOMYOPATHY	I420
CARDIOMEGALY	I517
ESSENTIAL (PRIMARY) HYPERTENSION	I10
**Stroke**	
INTRACEREBRAL HAEMORRHAGE, UNSPECIFIED	I619
CEREBRAL INFARCTION DUE TO THROMBOSIS OF PRECEREBRAL ARTERIES	I630
CEREB INFARCT DUE TO UNSPEC OCCL/STENOSIS OF PRECEREB ARTERIES	I632
CEREBRAL INFARCTION, UNSPECIFIED	I639
STROKE, NOT SPECIFIED AS HAEMORRHAGE OR INFARCTION	I64
CEREBROVASCULAR DISEASE, UNSPECIFIED	I679
SEQUELAE OF STROKE, NOT SPECIFIED AS HAEMORRHAGE OR INFARCTION	I694
SEQUELAE OF OTHER AND UNSPECIFIED CEREBROVASCULAR DISEASES	I698

Respiratory system

Respiratory infection

ACUTE UPPER RESPIRATORY INFECTION, UNSPECIFIED	J069
INFLUENZA WITH PNEUMONIA, OTHER INFLUENZA VIRUS IDENTIFIED	J100
INFLUENZA WITH OTHER RESP MANIFESTATIONS, OTHER INFLUENZA VIRUS IDENTIFIED	J101

For peer review only

Rates, causes, place, and predictors of mortality in adults with intellectual disabilities with and without Down syndrome: cohort study with record linkage

PNEUMONIA DUE TO STREPTOCOCCUS PNEUMONIAE	J13
BRONCHOPNEUMONIA, UNSPECIFIED	J180
LOBAR PNEUMONIA, UNSPECIFIED	J181
HYPOSTATIC PNEUMONIA, UNSPECIFIED	J182
PNEUMONIA, UNSPECIFIED	J189
UNSPECIFIED ACUTE LOWER RESPIRATORY INFECTION	J22
CHRONIC OBSTRUCTIVE PULMONARY DISEASE WITH ACUTE LOWER RESP INFECTION	J440
**Aspiration/reflux/choking**	
PNEUMONITIS DUE TO FOOD AND VOMIT	J690
GASTRO-OESOPHAGEAL REFLUX DISEASE WITHOUT OESOPHAGITIS	K219
INHALATION AND INGESTION OF FOOD CAUSING OBSTRUCTION OF RESPIRATORY TRACT	W79
FOREIGN BODY IN RESPIRATORY TRACT, PART UNSPECIFIED	T179
INHALATION/INGESTION OF OTHER OBJECTS CAUSING OBSTRUCT OF RESP TRACT	W80
**Other respiratory disorders**	
UNSPECIFIED CHRONIC BRONCHITIS	J42
EMPHYSEMA, UNSPECIFIED	J439
CHRONIC OBSTRUCTIVE PULMONARY DISEASE, UNSPECIFIED	J440
ASTHMA, UNSPECIFIED	J459
BRONCHIECTASIS	J47
OTHER INTERSTITIAL PULMONARY DISEASES WITH FIBROSIS	J841
PLEURAL EFFUSION, NOT ELSEWHERE CLASSIFIED	J90
CHRONIC RESPIRATORY FAILURE	J961
RESPIRATORY FAILURE, UNSPECIFIED	J969
OTHER SPECIFIED RESPIRATORY DISORDERS	J988
DYSPNOEA	R060
RESPIRATORY ARREST	R092
ASPHYXIATION	T71
UNSPECIFIED THREAT TO BREATHING	W84

Digestive system

**Ulcer/gastrointestinal perforation**	
OESOPHAGITIS	K20
PERFORATION OF INTESTINE (NONTRAUMATIC)	K631
PERITONITIS, UNSPECIFIED	K659
GASTRIC ULCER, CHRONIC OR UNSPECIFIED WITH PERFORATION	K255
OTHER PERITONITIS	K658
ACUTE PERITONITIS	K650
GASTROINTESTINAL HAEMORRHAGE, UNSPECIFIED	K922
ULCER OF INTESTINE	K633
**Other gastrointestinal disorders**	
BARRETTS OESOPHAGUS	K227
DIAPHRAGMATIC HERNIA WITHOUT OBSTRUCTION OR GANGRENE	K449
OTHER SPECIFIED NONINFECTIVE GASTROENTERITIS AND COLITIS	K528
ACUTE VASCULAR DISORDERS OF INTESTINE	K550
VASCULAR DISORDER OF INTESTINE, UNSPECIFIED	K559
VOLVULUS	K562
OTHER AND UNSPECIFIED INTESTINAL OBSTRUCTION	K566
CONSTIPATION	K590

For peer review only

Rates, causes, place, and predictors of mortality in adults with intellectual disabilities with and without Down syndrome: cohort study with record linkage

MEGACOLON, NOT ELSEWHERE CLASSIFIED	K593
ACUTE AND SUBACUTE HEPATIC FAILURE	K720
OTHER AND UNSPECIFIED CIRRHOSIS OF LIVER	K746
AUTOIMMUNE HEPATITIS	K754
INFLAMMATORY LIVER DISEASE, UNSPECIFIED	K759
OTHER SPECIFIED DISEASES OF LIVER	K768
CALCULUS OF GALLBLADDER WITH OTHER CHOLECYSTITIS	K801
CHOLANGITIS	K830
ACUTE PANCREATITIS, UNSPECIFIED	K859
PSEUDOCYST OF PANCREAS	K863
INTESTINAL MALABSORPTION, UNSPECIFIED	K909
DYSPHAGIA	R13

Genitourinary system

Renal failure

CHRONIC NEPHRITIC SYNDROME, UNSPECIFIED	N039
OTHER ACUTE RENAL FAILURE	N178
ACUTE RENAL FAILURE, UNSPECIFIED	N179
END-STAGE RENAL DISEASE	N180
CHRONIC KIDNEY DISEASE, STAGE 5	N185
CHRONIC KIDNEY DISEASE, UNSPECIFIED	N189
UNSPECIFIED KIDNEY FAILURE	N19

Chromosomal abnormalities

Down syndrome

DOWN'S SYNDROME, UNSPECIFIED	Q909

Other congenital condition

CONGENITAL HYDROCEPHALUS, UNSPECIFIED	Q039
SPINA BIFIDA, UNSPECIFIED	Q059
CONGENITAL MALFORMATION OF HEART, UNSPECIFIED	Q249
CONGENITAL DEFORMITY OF SPINE	Q675
CONGEN MALFORMATION SYNDROMES PREDOMINANTLY ASSOCIATED WITH SHORT STATURE	Q871
MARFAN'S SYNDROME	Q874
OTHER SPECIFIED CONGEN MALFORMATION SYNDROMES, NOT ELSEWHERE CLASSIFIED	Q878
CONGENITAL MALFORMATION, UNSPECIFIED	Q899
KLINEFELTER'S SYNDROME, UNSPECIFIED	Q984
FRAGILE X CHROMOSOME	Q992
OTHER LACK OF EXPECTED NORMAL PHYSIOLOGICAL DEVELOPMENT	R628

Other conditions occurring with small frequency

Other condition

DECUBITUS ULCER AND PRESSURE AREA	L89
SCOLIOSIS, UNSPECIFIED	M419
URETHRAL STRICTURE, UNSPECIFIED	N359
EPISTAXIS	R040
IMMOBILITY	R263
MALAISE AND FATIGUE	R53
GENERALIZED ENLARGED LYMPH NODES	R591

For peer review only

Rates, causes, place, and predictors of mortality in adults with intellectual disabilities with and without Down syndrome: cohort study with record linkage

INSUFFICIENT INTAKE OF FOOD AND WATER DUE TO SELF NEGLECT	R636
OTHER SPECIFIED GENERAL SYMPTOMS AND SIGNS	R688
OTHER ILL-DEFINED AND UNSPECIFIED CAUSES OF MORTALITY	R99
EXPOSURE TO UNSPECIFIED FACTOR	X59
MULTI-SYSTEM DEGENERATION	G903
BENIGN NEOPLASM, MENINGES, UNSPECIFIED	D329
AGRANULOCYTOSIS	D70
SARCOIDOSIS OF OTHER AND COMBINED SITES	D868
SARCOIDOSIS, UNSPECIFIED	D869
HYPOPITUITARISM	E230
HYPOTHYROIDISM, UNSPECIFIED	E039
OTHER THYROTOXICOSIS	E058
VOLUME DEPLETION	E86

Injuries and external causes

Injuries and accidents

INTRACRANIAL INJURY, UNSPECIFIED	S069
UNSPECIFIED INJURY OF HEAD	S099
INJURY OF COLON	S365
FRACTURE OF NECK OF FEMUR	S720
FRACTURE OF SHAFT OF TIBIA	S822
UNSPECIFIED MULTIPLE INJURIES	T07
FAT EMBOLISM (TRAUMATIC)	T791
SEQUELAE OF UNSPECIFIED INJURY OF HEAD	T909
UNSPECIFIED FALL	W19
SEQUELAE OF OTHER ACCIDENTS	Y86

Medical/surgical complication

POISONING BY OTHER ANTIDYSRHYTHMIC DRUGS, NOT ELSEWHERE CLASSIFIED	T462
ADVERSE EFFECTS OF OTHER ANTIDYSRHYTHMIC DRUGS, NOT ELSEWHERE CLASSIFIED	Y522
ABN REACT TO/LATER COMPLIC OF OP WITH IMPLANT OF ARTIFICIAL INTERN DEVICE	Y831
ABN REACT TO/LATER COMPLIC OF OP WITH ANASTOMOSIS, BYPASS OR GRAFT	Y832
ABN REACT TO/LATER COMPLIC OF OP WITH FORMATION OF EXTERNAL STOMA	Y833
ABNORMAL REACTION TO OR LATER COMPLICATION OF OTHER MEDICAL PROCEDURES	Y848
SEQ OF PROCED CAUSING ABN REACT/COMPLIC,W/O MENTION OF MISADV AT THE TIME	Y883
OTHER POSTPROCEDURAL RESPIRATORY DISORDERS	J958

For peer review only

Rates, causes, place, and predictors of mortality in adults with intellectual disabilities with and without Down syndrome: cohort study with record linkage

Supplementary table 3. Predictors of the outcome time to death from univariate analyses

Variable		N with event/ N in group	Hazard ratio (95% CI)	Individual p-value	Overall p-value
Demographics					
Age at time of health assessment		294/961	1.05 (1.04, 1.06)	<0.0001	
Sex	Male	154/525	0.88 (0.70, 1.11)	0.2730	
	Female	140/436	1.00 (-)		
Ability level	Mild ID	92/382	1.00 (-)		0.0007
	Moderate ID	73/236	1.38 (1.01, 1.87)	0.0411	
	Severe ID	67/180	1.75 (1.28, 2.40)	0.0005	
	Profound ID	62/163	1.77 (1.28, 2.45)	0.0005	
Type of accommodation	Family carer	70/374	1.00 (-)		<0.0001
	Independent of care	36/93	2.35 (1.57, 3.52)	<0.0001	
	Paid support	161/435	2.18 (1.65, 2.88)	<0.0001	
	Congregate	27/59	2.87 (1.84, 4.48)	<0.0001	
Neighbourhood deprivation	1 – most affluent	18/73	1.00 (-)		0.1890
	2	56/137	1.92 (1.13, 3.27)	0.0158	
	3	10/45	0.90 (0.42, 1.95)	0.7896	
	4	10/40	1.06 (0.49, 2.30)	0.8808	
	5	12/32	1.71 (0.82, 3.55)	0.1527	
	6	9/32	1.27 (0.57, 2.82)	0.5640	
	7	9/34	1.09 (0.49, 2.43)	0.8302	
	8	15/58	1.21 (0.61, 2.41)	0.5818	
	9	35/124	1.22 (0.69, 2.16)	0.4882	
	10 - most deprived	120/386	1.41 (0.86, 2.31)	0.1782	
Civil status	Single	288/938	1.28 (0.57, 2.87)	0.5485	
	Not single	6/23	1.00 (-)		
Employment/day activities	Yes	83/231	1.33 (1.03, 1.71)	0.0284	
	No	211/730	1.00 (-)		
Smoker	Yes	46/101	1.70 (1.24, 2.33)	0.0009	
	No	248/860	1.00 (-)		
**Health**					
Down syndrome	Yes	64/179	1.30 (0.98, 1.71)	0.0673	
	No	230/782	1.00 (-)		
Epilepsy	Yes	111/325	1.25 (0.99, 1.58)	0.0636	
	No	183/636	1.00 (-)		
Spastic quadriplegia	Yes	24/325	1.67 (1.10, 2.54)	0.0158	
	No	183/636	1.00 (-)		
Impaired mobility	Yes	195/735	0.51 (0.40, 0.65)	<0.0001	
	No	99 /226	1.00 (-)		
Body mass index	Underweight	9/43	0.63 (0.32, 1.25)	0.1847	0.1865
	Acceptable	83/265	1.00 (-)		
	Overweight	75/289	0.78 (0.57, 1.06)	0.1132	
	Obese	81/237	1.08 (0.80, 1.47)	0.6152	
	Morbidly obese	16/58	0.87 (0.51, 1.48)	0.6058	
Hearing impairment	Yes	112/267	1.79 (1.41, 2.26)	<0.0001	
	No	182/694	1.00 (-)		
Visual impairment	Yes	154/449	1.29 (1.02, 1.62)	0.0317	
	No	140/512	1.00 (-)		

For peer review only

Urinary incontinence	Yes	158/632	0.52 (0.41, 0.65)	<0.0001	
	No	136/329	1.00 (-)		
Bowel incontinence	Yes	197/733	0.55 (0.43, 0.70)	<0.0001	
	No	97/228	1.00 (-)		
Diabetes	Yes	29/47	2.72 (1.86, 4.00)	<0.0001	
	No	265/914	1.00 (-)		
PEG/tube fed	Yes	N/7	4.99 (2.22, 11.20)	0.0001	
	No	288/954			
Constipation	Yes	112/316	1.34 (1.06, 1.70)	0.0145	
	No	182/645	1.00 (-)		
Ataxia/gait disorder	Yes	104/276	1.50 (1.18, 1.90)	0.0009	
	No	190/685	1.00 (-)		
Nail disorder	Yes	74/223	1.18 (0.91, 1.54)	0.2120	
	No	220/738	1.00 (-)		
Epidermal thickening	Yes	66/207	1.10 (0.84, 1.45)	0.4947	
	No	228/754	1.00 (-)		
Cerebral palsy	Yes	54/175	1.02 (0.76, 1.37)	0.8792	
	No	240/786	1.00 (-)		
Osteoporosis	Yes	76/174	1.71 (1.32, 2.22)	<0.0001	
	No	218/786	1.00 (-)		
Fungal infection	Yes	42/158	0.83 (0.61, 1.18)	0.3366	
	No	252/803	1.00 (-)		
Hypertension	Yes	56/146	1.36 (1.01, 1.82)	0.0399	
	No	238/815	1.00 (-)		
Dysphagia	Yes	51/132	1.51 (1.11, 2.04)	0.0080	
	No	243/829	1.00 (-)		
Dyspnoea	Yes	49/130	1.41 (1.04, 1.92)	0.0285	
	No	245/831	1.00 (-)		
Muskuloskeletal pain	Yes	48/148	1.14 (0.83, 1.55)	0.4153	
	No	246/813	1.00 (-)		
Bone deformity	Yes	50/139	1.32 (0.97, 1.79)	0.0769	
	No	244/822	1.00 (-)		
Dental/oral problem	Yes	38/120	1.07 (0.76, 1.50)	0.7128	
	No	256/841	1.00 (-)		
Eczema/dermatitis	Yes	38/138	0.86 (0.61, 1.21)	0.3790	
	No	256/823	1.00 (-)		
GORD	Yes	51/133	1.43 (1.06, 1.94)	0.0198	
	No	243/828	1.00 (-)		
Lower respiratory tract infection	Yes	55/126	1.75 (1.30, 2.34)	0.0002	
	No	239/835	1.00 (-)		
Total number of physical conditions		294/961	1.06 (1.04, 1.08)	<0.0001	
Psychosis	Yes	11 /42	0.81 (0.44, 1.48)	0.4990	
	No	283 /919	1.00 (-)		
Affective disorder including bipolar	Yes	24/68	1.19 (0.78, 1.80)	0.4216	
	No	270/893	1.00 (-)		
Autism	Yes	13/69	0.54 (0.31, 0.94)	0.0306	
	No	281/892	1.00 (-)		
Problem behaviour	Yes	71/218	1.09 (0.83, 1.42)	0.5251	
	No	223/743	1.00 (-)		
Eating disorder, including pica	Yes	5/17	0.99 (0.41, 2.40)	0.9857	
	No	289/944	1.00 (-)		
Any mental illness, excluding problem behaviours	Yes	73/217	1.16 (0.89, 1.51)	0.2849	
	No	221/744	1.00 (-)		
Service use					
Number of GP consultations in last 12 months		287/951	1.05 (1.03, 1.06)	<0.0001	

For peer review only

Rates, causes, place, and predictors of mortality in adults with intellectual disabilities with and without Down syndrome: cohort study with record linkage

Number of A&E attendances in last 12 months		280/938	1.09 (0.99, 1.20)	0.0847	
Number of health professions providing care		294 /961	1.10 (1.03, 1.16)	0.0023	
Prescriptions					
Antipsychotics	Yes	79/226	1.12 (0.94, 1.57)	0.1421	
	No	215/735	1.00 (-)		
Antidepressants	Yes	39/118	1.16 (0.83, 1.63)	0.3778	
	No	255/843	1.00 (-)		
Anxiolytic/hypnotics	Yes	20/68	0.95 (0.60, 1.49)	0.8159	
	No	274/893	1.00 (-)		
Antiepileptics	Yes	90/253	1.31 (1.02, 1.68)	0.0315	
	No	204/708	1.00 (-)		
Number of drug classes taken		294/961	1.16 (1.12, 1.21)	<0.0001	

A&E=accident and emergency; CI=confidence interval; GORD=gastro-oesophageal reflux disorder; PEG=percutaneous endoscopic gastrostomy

For peer review only

STROBE Statement—checklist of items that should be included in reports of observational studies

	Item No.	Recommendation	Page No.	Relevant text from manuscript
**Title and abstract**	1	(*a*) Indicate the study's design with a commonly used term in the title or the abstract		p1
		(*b*) Provide in the abstract an informative and balanced summary of what was done and what was found		p2
**Introduction**				
Background/rationale	2	Explain the scientific background and rationale for the investigation being reported		p4-6, supplementary table 1
Objectives	3	State specific objectives, including any prespecified hypotheses		6, paragraph 3
**Methods**				
Study design	4	Present key elements of study design early in the paper		p6-10, supplementary tables 2/3
Setting	5	Describe the setting, locations, and relevant dates, including periods of recruitment, exposure, follow-up, and data collection		p7, paragraph 1, 7-8
Participants	6	(*a*) *Cohort study*—Give the eligibility criteria, and the sources and methods of selection of participants. Describe methods of follow-up<br>*Case-control study*—Give the eligibility criteria, and the sources and methods of case ascertainment and control selection. Give the rationale for the choice of cases and controls<br>*Cross-sectional study*—Give the eligibility criteria, and the sources and methods of selection of participants		p7, paragraph 1
		(*b*) *Cohort study*—For matched studies, give matching criteria and number of exposed and unexposed<br>*Case-control study*—For matched studies, give matching criteria and the number of controls per case		p7, paragraph 2, p9, paragraph 4
Variables	7	Clearly define all outcomes, exposures, predictors, potential confounders, and effect modifiers. Give diagnostic criteria, if applicable		p7-8, supplementary table 2
Data sources/ measurement	8*	For each variable of interest, give sources of data and details of methods of assessment (measurement). Describe comparability of assessment methods if there is more than one group		p7-8, p9, paragraph 4
Bias	9	Describe any efforts to address potential sources of bias		p9, paragraph 4
Study size	10	Explain how the study size was arrived at		P7, paragraph2, p9, paragraph 4

Continued on next page

For peer review only

Quantitative variables	11	Explain how quantitative variables were handled in the analyses. If applicable, describe which groupings were chosen and why	p8-10
Statistical methods	12	(*a*) Describe all statistical methods, including those used to control for confounding	p8-10
		(*b*) Describe any methods used to examine subgroups and interactions	p8-10
		(*c*) Explain how missing data were addressed	p11, paragraph 2
		(*d*) *Cohort study*—If applicable, explain how loss to follow-up was addressed<br>*Case-control study*—If applicable, explain how matching of cases and controls was addressed<br>*Cross-sectional study*—If applicable, describe analytical methods taking account of sampling strategy	p11, paragraph 2
		(*e*) Describe any sensitivity analyses	N/A
**Results**			
Participants	13*	(a) Report numbers of individuals at each stage of study—eg numbers potentially eligible, examined for eligibility, confirmed eligible, included in the study, completing follow-up, and analysed	p11, paragraph 2
		(b) Give reasons for non-participation at each stage	p11, paragraph 2
		(c) Consider use of a flow diagram	-
Descriptive data	14*	(a) Give characteristics of study participants (eg demographic, clinical, social) and information on exposures and potential confounders	p11-12, Table 1, supplementary table 3
		(b) Indicate number of participants with missing data for each variable of interest	table 1, supplementary table 3
		(c) *Cohort study*—Summarise follow-up time (eg, average and total amount)	p12, paragraph 1
Outcome data	15*	*Cohort study*—Report numbers of outcome events or summary measures over time	p11, paragraph 2
		Case-control study—Report numbers in each exposure category, or summary measures of exposure	
		Cross-sectional study—Report numbers of outcome events or summary measures	
Main results	16	(*a*) Give unadjusted estimates and, if applicable, confounder-adjusted estimates and their precision (eg, 95% confidence interval). Make clear which confounders were adjusted for and why they were included	P15, table 6
		(*b*) Report category boundaries when continuous variables were categorized	N/A
		(*c*) If relevant, consider translating estimates of relative risk into absolute risk for a meaningful time period	-

Continued on next page

For peer review only - http://bmjopen.bmj.com/site/about/guidelines.xhtml
For peer review only

Other analyses	17	Report other analyses done—eg analyses of subgroups and interactions, and sensitivity analyses	p11-16
**Discussion**			
Key results	18	Summarise key results with reference to study objectives	p16, paragraph 2
Limitations	19	Discuss limitations of the study, taking into account sources of potential bias or imprecision. Discuss both direction and magnitude of any potential bias	p20, paragraph 1
Interpretation	20	Give a cautious overall interpretation of results considering objectives, limitations, multiplicity of analyses, results from similar studies, and other relevant evidence	p20, paragraph 2
Generalisability	21	Discuss the generalisability (external validity) of the study results	p20, paragraph 1
**Other information**			
Funding	22	Give the source of funding and the role of the funders for the present study and, if applicable, for the original study on which the present article is based	P24

*Give information separately for cases and controls in case-control studies and, if applicable, for exposed and unexposed groups in cohort and cross-sectional studies.

Note: An Explanation and Elaboration article discusses each checklist item and gives methodological background and published examples of transparent reporting. The STROBE checklist is best used in conjunction with this article (freely available on the Web sites of PLoS Medicine at http://www.plosmedicine.org/, Annals of Internal Medicine at http://www.annals.org/, and Epidemiology at http://www.epidem.com/). Information on the STROBE Initiative is available at www.strobe-statement.org.

For peer review only

BMJ Open

Rates, causes, place, and predictors of mortality in adults with intellectual disabilities with and without Down syndrome: cohort study with record linkage

Journal:	*BMJ Open*
Manuscript ID	bmjopen-2019-036465.R2
Article Type:	Original research
Date Submitted by the Author:	21-Apr-2020
Complete List of Authors:	Cooper, Sally-Ann; University of Glasgow, Institute of Health and Wellbeing<br>Allan, Linda; University of Glasgow, Institute of Health and Wellbeing<br>Greenlaw, Nicola; University of Glasgow, Robertson Centre for Biostatistics, Institute of Health and Wellbeing<br>Mcskimming, Paula; University of Glasgow, Robertson Centre for Biostatistics, Institute of Health and Wellbeing<br>Jasilek, Adam; University of Glasgow, Robertson Centre for Biostatistics, Institute of Health and Wellbeing<br>Henderson, Angela; University of Glasgow, Institute of Health and Wellbeing<br>McCowan, Colin ; University of St. Andrews<br>Kinnear, Deborah; University of Glasgow, Institute of Health and Wellbeing<br>Melville, Craig; University of Glasgow, Institute of Health and Wellbeing
**Primary Subject Heading**:	Public health
Secondary Subject Heading:	Mental health, Epidemiology
Keywords:	PUBLIC HEALTH, Adult psychiatry < PSYCHIATRY, EPIDEMIOLOGY, GENERAL MEDICINE (see Internal Medicine)

SCHOLARONE™
Manuscripts

BMJ

I, the Submitting Author has the right to grant and does grant on behalf of all authors of the Work (as defined in the below author licence), an exclusive licence and/or a non-exclusive licence for contributions from authors who are: i) UK Crown employees; ii) where BMJ has agreed a CC-BY licence shall apply, and/or iii) in accordance with the terms applicable for US Federal Government officers or employees acting as part of their official duties; on a worldwide, perpetual, irrevocable, royalty-free basis to BMJ Publishing Group Ltd ("BMJ") its licensees and where the relevant Journal is co-owned by BMJ to the co-owners of the Journal, to publish the Work in this journal and any other BMJ products and to exploit all rights, as set out in our licence.

The Submitting Author accepts and understands that any supply made under these terms is made by BMJ to the Submitting Author unless you are acting as an employee on behalf of your employer or a postgraduate student of an affiliated institution which is paying any applicable article publishing charge ("APC") for Open Access articles. Where the Submitting Author wishes to make the Work available on an Open Access basis (and intends to pay the relevant APC), the terms of reuse of such Open Access shall be governed by a Creative Commons licence – details of these licences and which Creative Commons licence will apply to this Work are set out in our licence referred to above.

Other than as permitted in any relevant BMJ Author's Self Archiving Policies, I confirm this Work has not been accepted for publication elsewhere, is not being considered for publication elsewhere and does not duplicate material already published. I confirm all authors consent to publication of this Work and authorise the granting of this licence.

For peer review only

Rates, causes, place, and predictors of mortality in adults with intellectual disabilities with and without Down syndrome: cohort study with record linkage

*Sally-Ann Cooper

Linda Allan

Nicola Greenlaw

Paula McSkimming

Adam Jasilek

Angela Henderson

Colin McCowan

Deborah Kinnear

Craig Melville

*Corresponding author: Sally-Ann.Cooper@glasgow.ac.uk

For peer review only

For peer review only - http://bmjopen.bmj.com/site/about/guidelines.xhtml

Abstract

Objectives

To investigate mortality in adults with intellectual disabilities: rates, causes, place, demographic and clinical predictors.

Design

Cohort study with record linkage to death data.

Setting

General community.

Participants

961/1,023 (94%) adults (16-83 years; mean=44.1 years; 54.6% male) with intellectual disabilities, clinically examined in 2001-2004; subsequently record-linked to their National Health Service number, allowing linkage to death certificate data, 2018.

Outcome measures

Standardised mortality ratios (SMRs), underlying, and all contributing causes of death, avoidable deaths, place, and demographic and clinical predictors of death.

Results

294/961 (30.6%) had died; 64/179 (35.8%) with Down syndrome, 230/783 (29.4%) without Down syndrome. SMR overall=2.24 (1.98, 2.49); Down syndrome adults=5.28 (3.98, 6.57), adults without Down syndrome=1.93 (1.68, 2.18); male=1.69 (1.42, 1.95), female=3.48 (2.90, 4.06). SMRs decreased as age increased. More severe intellectual disabilities increased SMR, but ability was not retained in the multivariable model. SMRs were higher for most ICD-10 chapters. For adults without Down syndrome, aspiration/reflux/choking, and respiratory infection were the commonest underlying causes of mortality; for Down syndrome adults “Down syndrome”, and dementia were most common. Amenable deaths (29.8%) were double that in the general population (14%). 60.3% died in hospital. Mortality risk related to: percutaneous endoscopic gastrostomy/tube fed, Down syndrome, diabetes, lower respiratory tract infection at

For peer review only

cohort-entry, smoking, epilepsy, hearing impairment, increasing number of prescribed drugs, increasing age. Bowel incontinence reduced mortality risk.

Conclusions

Adults with intellectual disabilities with and without Down syndrome have different SMRs and causes of death which should be separately reported. Both die younger, from different causes than other people. Some mortality risks are similar to other people, with earlier mortality reflecting more multi-morbidity; amenable deaths are also common. This should inform actions to reduce early mortality, e.g. training to avoid aspiration/choking, pain identification to address problems before they are advanced, and reasonable adjustments to improve health-care quality.

Strengths and limitations of this study

- Thorough methods of case ascertainment for intellectual disabilities at baseline.
- Individual verification of intellectual disabilities and its severity, and detailed health assessments at baseline.
- Longitudinal design.
- Large cohort size and study duration, and successful record linkage for 94% of participants.
- Limitations include that the study was conducted in only one part of Scotland, and the reliance upon recorded cause of death from death certificates.

Word count: 5,610

For peer review only - http://bmjopen.bmj.com/site/about/guidelines.xhtml

Introduction

People with intellectual disabilities die at a younger age than other people; on average, 20 years younger,[1] or 28 years younger specifically for people with Down syndrome.[2] It has been demonstrated that people with intellectual disabilities receive poorer management of their long term conditions within primary health care services compared with the general population,[3] and it is conceivable that this is one contributor to earlier mortality. It has been suggested that as many as 40% of deaths of people with intellectual disabilities may have been amenable to good quality health care.[4-6] There has been a recent increase in research on mortality in people with intellectual disabilities, but very little research has distinguished people with intellectual disabilities with and without Down syndrome, or investigated the factors associated with risk of mortality, and causes of mortality.

Previous studies on death in people with intellectual disabilities had limitations such as small sample sizes, or non-representative populations. More recently, there have been large-scale studies which are more representative, having been drawn from intellectual disabilities registers, or social security or primary care data with record linkage to death certification. These have been undertaken in parts of Sweden, Australia, England, Finland, Canada, Ireland, and USA (supplementary table 1).[5-19] These studies fairly consistently report standardised mortality ratios (SMRs) to be high for people with intellectual disabilities, more so at younger ages, and higher for women than men. Adult studies have tended to report SMRs in the region of 2-4, although in some, SMR is only slightly above 1.[10,16,19] However, direct comparison between studies is not always possible, due to the different age ranges studied and methods of reporting.

- Supplementary table 1 -

For peer review only

In view of the methods that studies have used for population identification (typically, routine administrative data linked to death certifications), they provide little information on the socio-clinical factors that influence SMR, or the risk factors associated with death, beyond that of age and sex. Three studies reported SMR by level of intellectual disabilities, with, broadly speaking, higher SMR with more severe intellectual disabilities.[7,10,17] Only three studies (different studies to those that reported on level of intellectual disabilities) were able to report data separately for adults with intellectual disabilities with and without Down syndrome; two found higher mortality rates for adults with Down syndrome (SMR=7.6,[9] and hazard ratio=9.21[5]) than for adults without Down syndrome, or an odds ratio showing Down syndrome as a risk of death.[12] A further study reported SMR=5.5 for children and adults (combined) with Down syndrome, but did not report SMR for those with intellectual disabilities without Down syndrome.[20] Two studies reported adults with intellectual disabilities to have higher SMRs if they have the co-morbidities of epilepsy,[5,7] and cerebral palsy,[7] as opposed to not having these comorbidities. One study reported adults with intellectual disabilities with comorbid autism to have lower risk of mortality than those without comorbid autism.[5] One study reported the risk factors for mortality in a population with intellectual disabilities to be: age, Down syndrome, cerebral palsy, blindness/low vision, technological dependence/medical fragility, wheelchair dependence, mobility impairment without wheelchair dependence, and epilepsy.[12] Factors not found to be risks, if any, were not reported, and a further limitation was that factors were reported by agency staff, rather than the individuals undergoing health assessments.[12] We have not identified any other studies that investigated risk factors for time to mortality in adults with intellectual disabilities.

There is less consistency regarding the most common certified underlying causes of death in adults with intellectual disabilities, partly as some studies do not report these separately for children and adults, or by age ranges. Additionally, studies group causes of death in different ways (e.g. pneumonia versus respiratory system), which can affect

For peer review only - http://bmjopen.bmj.com/site/about/guidelines.xhtml

prevalence rankings between studies. Pneumonia, other respiratory diseases, and diseases of the nervous system were reported to be the most common in one study,[11] diseases of the circulatory system and respiratory systems in another,[5] heart disease, neoplasm, and Alzheimer disease in a third,[18] and diseases of the circulatory system, neoplasm, and the nervous system in a fourth.[19] In adults with intellectual disabilities, cause specific SMRs have been reported to be high across most groups of disorders.[5,11] These studies did not report cause of death separately for adults with and without Down syndrome. Given the different health profile of people with Down syndrome compared with people with intellectual disabilities of other causes, this is an important limitation.[21] In people with Down syndrome, most studies on mortality have been conducted with child populations, and report the most common causes of death to be congenital heart disease, and pneumonia/diseases of the respiratory system.[2]

Overall, the existing body of literature on mortality in adults with intellectual disabilities does not include more detailed information on level of intellectual disabilities, nor separate out the population with, from those without, Down syndrome (for whom causes of death may differ), nor investigate health and demographic predictors of death other than age and sex, and is inconsistent with regards to causes of death. A better understanding of these factors may provide a pathway to action to reduce the observed earlier mortality in adults with intellectual disabilities.

This study aims to investigate the rates, causes, place, and demographic and clinical associations with mortality in adults with intellectual disabilities, with and without Down syndrome.

Methods

Approval

For peer review only - http://bmjopen.bmj.com/site/about/guidelines.xhtml

Ethical approved was gained from NHS Greater Glasgow Primary Care Trust - Community & Mental Health Research Ethics Committee, and NHS Greater Glasgow and Clyde Safe Haven. Individual consent to participate was taken in line with Scottish law, between 2001-2004.

Participants

The adult (aged 16+ years) intellectual disabilities population living within the NHS Greater Glasgow area was identified through multiple sources between 2000-2001. General practitioners were financially incentivised to identify their registered patients with intellectual disabilities, and all 631 (100%) did so. Adults were also identified via the intellectual disabilities health and social work services including day services, the Health Board register, and records of financial payments for any service by social work. This process led initially to an over-identification, such as people with IQ scores in the 70–80 range with additional complex health needs. All were systematically reviewed by nurses in the intellectual disabilities health service, and this group were removed. Thus, a register was compiled, and subsequently updated annually via general practices, with central support from the intellectual disabilities health service, until 2017 when services were redesigned. The identified adult prevalence of intellectual disabilities within the area was 3.33 per 1,000 in 2000-2001.

Process and data collection

With initial piloting in 2001, each participant had a detailed assessment of their general and mental health, and demographic factors, completed 2002-2004. One of six specially trained, registered nurses reviewed each person's primary health care records, then used a semi-structured tool, the C21st Health Check, to assess clinical factors and the level and cause of intellectual disabilities. In addition to a review of existing health problems and all bodily health systems, a physical examination was undertaken, including assessment of vision and hearing, measurement of height and weight, and a phlebotomy protocol followed. All information was then reviewed by the nurse with one

For peer review only - http://bmjopen.bmj.com/site/about/guidelines.xhtml

of three general practitioners with a special interest in intellectual disabilities, and any further investigations that were indicated were completed. Previously known, and newly identified, conditions were then classified using the *International Statistical Classification of Diseases and Related Health Problems, 10th Revision (ICD-10).*[22] Anyone identified to have possible, probable, or definite mental ill-health, autism, or problem behaviours was then fully assessed by the project's intellectual disabilities psychiatrists. Each person's assessment findings were then case conferenced by the two Consultant psychiatrists, and diagnoses were derived and agreed according to clinical diagnoses, *ICD-10 (Diagnostic Criteria for Research),*[23] *Diagnostic and Statistical Manuel of Mental Disorders-IV-TR,*[24] and *Diagnostic Criteria for Psychiatric Disorders for use with Adults with Learning Disabilities (DC-LD).*[25] Information was also collected on demographics, and community, hospital, and social service use. Further details are provided elsewhere.[26,27] The data were entered into a database by two dedicated data-entry staff.

Each person in Scotland is given a number unique to them at birth or first registration with a general practitioner, which is used in almost all subsequent health service encounters, and on certification of death. The numbers are held on the Community Health Index (CHI) database at National Services Scotland. These CHI numbers provided a means to record link each participant with National Records for Scotland death certification data. This linkage was performed in 2018, and the linked data were held in the NHS Greater Glasgow & Clyde (NHS GG&C) Safe Haven. Data on immediate, underlying, and contributory causes of deaths by ICD-10 codes, age at death, and place of death were extracted.

In order to provide finer granularity of cause of death, two clinical academics then grouped specific causes of death into narrower groupings than those provided by ICD-10 chapter headings (supplementary table 2). This approach was also in view of the recognised issue of variation between health staff in distinguishing and recording immediate causes of death, and because some causes occurred in low numbers so could

For peer review only - http://bmjopen.bmj.com/site/about/guidelines.xhtml

not be individually reported due to the risk of statistical disclosure. Additionally, some conditions likely to be the same are spilt between different ICD-10 chapters, e.g. dementia in Alzheimer disease (F00) and unspecified dementia (F03) in the ICD-10 mental and behavioural disorders chapter, and Alzheimer's disease (G30) and Alzheimer's disease, unspecified (G30.9) in the ICD-10 diseases of the nervous system chapter. A list of related conditions was generated by one of the clinical academics and then checked by the second.

- Supplementary table 2 –

Analyses

All statistical analyses were conducted using R for Windows v3.3.0 or SAS 9.3 (SAS Institute, Cary NC) and were performed within the NHS GG&C Safe Haven environment. Due to disclosure principles of the Safe Haven, results with counts of less than 5 cannot be released; these have been referred to as <5 throughout. Similarly, if it is deemed possible that participants may be identified from the results, these may be omitted. Details are provided if this occurred.

Data were summarised for the population of adults aged 16+ years with intellectual disabilities. Categorical variables were summarised with the number and percentages of people falling into each category and the number of missing data. Continuous variables were summarised with the number of observations and those missing, the mean and standard deviation (SD), and the minimum and maximum values, unless otherwise stated.

Participant characteristics were summarised overall and for those alive and those deceased. For those who are deceased, their data including age at death, underlying/contributing causes of death, and location of death were summarised for those with and without Down syndrome. Location codes for place of death are provided

For peer review only - http://bmjopen.bmj.com/site/about/guidelines.xhtml

where available. We assumed those with the code for non-institutional location to have died at home. Due to small numbers, location codes have been grouped together for NHS hospitals, home, and other hospitals/care facilities including hospices.

Mortality incidence rates have been calculated using the number of deaths in the cohort divided by the number of person years alive within the study period multiplied by 100,000, overall and for those with and without Down syndrome. SMRs were calculated using population data for those aged 15 and over within NHS GG&C in 2010.[28,29] Death rates for males and females by 5 year band ages groups spanning from 15-20 years old to 90 years and over were summed to form the expected death rates for the general population. The observed death rate for adults with intellectual disabilities was taken from our study results. The observed/expected death rates were calculated for the intellectual disabilities cohort overall then separately by age group, sex, ability level, and for the adults with, and without, Down syndrome, and ICD-10 chapter for cause of death, and compared to the general population.

Deaths were also analysed for those that could be considered as deaths that would have been avoidable. The Office for National Statistics (ONS) published a definition of avoidable mortality,[30] which lists the causes of amenable deaths (deaths that should not occur in the presence of good health care, e.g. respiratory disease), and causes of preventable deaths (e.g. from diseases that could have been avoided by prior immunisation), by ICD-10 codes. Causes of death for the adults with intellectual disabilities have been summarised by ONS definition of avoidable deaths.

To determine the demographic and clinical factors associated with death in adults with intellectual disabilities, time to event analyses were explored using univariate Cox Proportional Hazards models. Variables were selected as potentially relevant on the basis of what is known on causes of death in people with intellectual disabilities, the 20 most common physical health conditions reported in the adult population with intellectual

For peer review only - http://bmjopen.bmj.com/site/about/guidelines.xhtml

disabilities,[21] and other factors hypothesised as potentially clinically relevant (supplementary table 3):

- Demographics - 9 variables.
- Clinical conditions - 33 variables.
- Service use - 3 variables.
- Prescriptions - 5 variables.

All 50 variables were then permitted entry in to a single multivariable analysis using stepwise regression methods, in order to identify a model containing the statistically significant factors associated with death. Age at date of the health assessment was entered in to the model as a continuous measure. Results from the univariate Cox Proportional Hazards models (Supplementary table 3) and the statistically significant multivariable model from the stepwise results have been presented with hazard ratios with corresponding 95% confidence intervals (HR, 95% CI) and p-values were obtained.

- Supplementary table 3 –

Patient and public involvement

This study was designed to respond to the growing concern expressed by people with intellectual disabilities, their families, and third sector organisations about the early deaths of people with intellectual disabilities. The Scottish Learning Disabilities Observatory, where this research was undertaken, has a specific remit for people with intellectual disabilities. Its steering group includes partners from third sector organisations, including Down syndrome Scotland, and people with intellectual disabilities, who approved the work plan for this project prior to it commencing. Results from this study will be disseminated for people with intellectual disabilities in an easy-read version via the Scottish Learning Disabilities Observatory.

Results

For peer review only - http://bmjopen.bmj.com/site/about/guidelines.xhtml

Population characteristics

962 of the original 1,023 (94.0%) adults with intellectual disabilities who were assessed were linked to a CHI number enabling the extraction of relevant death data. Reasons for the unlinked 61 people could be due to moving out of the area, or a recording mistake. 1 further participant was removed from the analysis due to inaccurate recording of dates, leaving 961 adults in the cohort (93.9%). Of these 961 adults, 294 (30.6%) had a record of death. Table 1 shows the baseline characteristics of the full cohort of 961, the adults who died, and those still alive at the time of linkage.

- Insert table 1 about here –

Age at death, and mortality incidence

The mean age at death was 61.0 years (SD=7.0 years). Of the 961 adults, 64 (35.8%) of the 179 adults with Down syndrome, and 230 (29.4%) of the 782 adults without Down syndrome had a record of death. Their mean age of death was 56.9 years (SD=4.3 years) for the adults with Down syndrome, and 62.2 years (SD=7.5 years) for the adults without Down syndrome. Mortality incidence for the cohort during the study period was 3,049.0 per 100,000 person years follow-up, with 3,832.1 per 100,000 for those with Down Syndrome and 2,885.0 for those without Down syndrome.

Standardised mortality ratios

Compared with the general population, the SMR was 2.24 (1.98, 2.49) overall; 5.28 (3.98, 6.57) for adults with Down syndrome, 1.93 (1.68, 2.18) for adults without Down syndrome; 1.69 (1.42, 1.95) for men and 3.48 (2.90, 4.06) for women. SMRs were higher the more severe the level of intellectual disabilities, with people with profound intellectual disabilities having an SMR of 4.14 (3.11, 5.17). SMR was high for all age groups (though for the 15-25 year age group, the wide confidence interval includes one, perhaps due to the smaller number of deaths in this group); this decreased as age increased. SMRs were high for most ICD-10 chapter groups of conditions, particularly so

For peer review only - http://bmjopen.bmj.com/site/about/guidelines.xhtml

for congenital malformations at 17.26 (10.75, 23.78), diseases of the digestive system at 16.13 (8.23, 24.04), mental and behavioural disorders at 12.64 (3.27, 22.00), and external causes at 11.08 (3.40, 18.76). Details are shown in table 2.

- Insert table 2 about here –

Causes of death

Cause of death data was available from death certificates for 262 (89.1%) of 294 participants who had died, which include 57 (89.1%) participants with Down syndrome, and 205 (88.7%) participants without Down syndrome. Table 3 shows the underlying causes of death by ICD-10 chapters separately for the adults with, and without Down syndrome. For the whole cohort, diseases of the respiratory system were the most common (21.8%), then diseases of the circulatory system (19.1%), then diseases of the nervous system (13.0%), and neoplasms, followed by congenital anomalies (10.3%). For the adults with Down syndrome, congenital anomalies were the most common (in all cases this was a record of "Down syndrome"), then jointly diseases of the respiratory system and diseases of the circulatory system, then diseases of the nervous system, followed by infections, and mental and behavioural disorders. For the adults without Down syndrome, diseases of the respiratory system were the most common, then diseases of the circulatory system, then neoplasms, then diseases of the nervous system, followed by diseases of the digestive system. Table 4 presents the most common underlying causes of death by individual causes, or related groups of causes, with finer granularity than ICD-10 chapter headings (groups are shown in supplementary table 2). Causes are listed in the order of how common they were in the whole cohort. Data are presented separately for the adults with, and without Down syndrome. For the whole cohort, the most common cause was aspiration/reflux/choking, then respiratory infection, then other malignancy (non gastrointestinal), then other condition (mostly unrelated conditions that could not be reported individually or as groups, due to individually occurring at a frequency of <5). For the adults with Down syndrome, Down

For peer review only

syndrome was the most common cause, then dementia, then other infection. For the adults without Down syndrome, aspiration/reflux/choking was the most common cause, then respiratory infection, then other malignancy (non gastrointestinal). For the 21 people whose death certificate listed Down syndrome as their underlying cause of death, the death certificates were reviewed and underlying cause of death reclassified, as a sensitivity check. Following this, the most common underlying causes of death for the adults with Down syndrome were dementia (n=20; 35.1%), then other infection (n=7; 12.3%).

- Insert tables 3 and 4 about here -

Table 5 shows the all contributing causes of death data, again presenting the most common causes by individual causes, or related groups of causes with finer granularity than ICD-10 chapter headings. Data is presented separately for the adults with, and without Down syndrome. For the whole cohort, respiratory infection was the most common cause (27.1%), followed by aspiration/reflux/choking (19.8%), other conditions (15.6%), other cardiovascular conditions (non acute myocardial nor other ischaemic heart disease: 14.5%), then other respiratory conditions. For the adults with Down syndrome, Down syndrome was the most common, then dementia, then respiratory infection, then aspiration/reflux/choking. For the adults without Down syndrome, respiratory infection was the most common cause, then aspiration/reflux/choking, then other condition, then other respiratory conditions, and intellectual disabilities.

- Insert table 5 about here –

Avoidable deaths

According to the ONS list of avoidable deaths, 102 (38.9%) of the 262 deaths were avoidable; most notably, respiratory infection and epilepsies (table 4). 78 (29.8%) were deaths that are amenable to good health care, whilst 51 (19.5%) were preventable

For peer review only - http://bmjopen.bmj.com/site/about/guidelines.xhtml

deaths. 27 (10.3%) deaths were classed as both amenable and preventable deaths. This compares to published Scottish death data showing in 2018 that 28% of deaths were avoidable; 14% amenable and 24% preventable, similar to the figures in the previous four years (data not available prior to 2014).[31] For the 57 deaths of adults with Down syndrome, 17 (29.8%) deaths were avoidable, 15 (26.3%) deaths were amenable to good health care, whilst 7 (12.3%) were preventable. 5 (8.8%) were both amenable and preventable. For the 205 deaths of adults without Down syndrome, 85 (41.5%) were avoidable, 63 (30.7%) deaths were amenable to good health care, whilst 44 (21.5%) were preventable. 22 (10.7%) were both amenable and preventable.

Place of death

Of the 262 participants for whom place of death was known, 158 (60.3%) died in an NHS Hospital, 70 (26.7%) died at home, and 34 (13.0%) died within other hospitals/care facilities. This was similar for both the adults with Down syndrome: 31 (54.4%) in an NHS hospital, 17 (29.8%) at home, and 9 (15.8%) within other hospitals/care facilities; and the adults without Down syndrome: 127 (62.0%) in an NHS hospital, 53 (25.9%) at home, and 25 (12.2%) within other hospitals/care facilities.

Factors associated with risk of death

The results from the univariate cox proportional hazards models indicated that of the original 50 potential variables, factors associated with risk of death were (supplementary table 3):

- Demographics – age at the time of the health assessment, more severe learning disabilities, accommodation type (not living with family carer), not having day-time occupation, and being a smoker (but not sex, the extent of neighbourhood deprivation, civil status, nor Down syndrome, in view of the confidence intervals).
- Clinical conditions – having spastic quadriplegia, hearing impairment, visual impairment, diabetes, percutaneous endoscopic gastrostomy/tube fed, constipation, ataxia/gait disorder, osteoporosis, hypertension, dysphagia, dyspnoea, gastro-

For peer review only - http://bmjopen.bmj.com/site/about/guidelines.xhtml

oesophageal reflux disorder, lower respiratory tract infection, total number of physical health disorders, not having impaired mobility, not having urinary incontinence, not having bowel incontinence, and not having autism (but not epilepsy, body mass index, nail disorder, epidermal thickening, cerebral palsy, fungal infection, musculoskeletal pain, bone deformity, dental/oral problem, eczema/dermatitis, psychosis, affective disorder including bipolar affective disorder, problem behaviour, eating disorder including pica, nor any mental illness).

- Service use – number of general practitioner consultations in the previous 12 months, total number of different types of health professionals providing care at the time of the clinical assessment, (but not number of accident and emergency attendances in the previous 12 months).
- Prescriptions – antiepileptic drugs, total number of different types of drugs, (but not antipsychotic drugs, antidepressant drugs, nor anxiolytic drugs).

Table 6 shows the final model of the variables retained in the multivariable analysis for time to death. The significant factors indicating an increased risk of death were increased age at the time of the health assessment, smoking, Down syndrome, diabetes, being percutaneous endoscopic gastrostomy/tube fed, lower respiratory tract infection at cohort inception, epilepsy, hearing impairment, and total number of different types of drugs prescribed, whilst bowel incontinence showed a reduced risk of death. Of note, level of intellectual disabilities whilst significant in the univariate analysis, was not retained in the multivariable model.

- Insert table 6 about here –

Discussion

Principle findings and interpretation

For peer review only - http://bmjopen.bmj.com/site/about/guidelines.xhtml

For peer review only

As far as we are aware, this is the first population-based study of adults with intellectual disabilities to report in detail the factors associated with time to death, and to describe their causes of death and quantify the SMR separately for adults with Down syndrome and adults without Down syndrome. This is important, since adults with Down syndrome form a notable proportion of all adults with intellectual disabilities (19% in this cohort), and because they have a different pattern of clinical conditions compared with other adults with intellectual disabilities.[21] We found that aspiration/reflux/choking is the most common underlying cause of death in adults with intellectual disabilities, followed by respiratory infection. They are also the most common all contributing causes of death. The profile differed in the adults with Down syndrome for whom “Down syndrome”, followed by dementia, were recorded as the most common underlying cause of death, and all contributing causes of death (or alternatively, dementia, then other infection were the most common underlying causes when “Down syndrome” deaths were reclassified); with the next most common all contributing cause of death being respiratory infection, then aspiration/reflux/choking. The proportion of deaths that would have been amenable to good care for adults with intellectual disabilities was more than double that seen in the general population. Although aspiration/reflux/choking is not included in the ONS list of avoidable deaths, and therefore not included in the figures we report on amenable deaths, we consider that good care could have prevented many of these deaths. This appears to be very important for adults with intellectual disabilities irrespective of whether they have Down syndrome. Similarly, some other causes of deaths within this cohort (supplementary table 2), such as constipation/mega-colon, and urinary tract infections do not appear on the ONS list of avoidable deaths.

Clearly, this pattern of causes of death differs from that seen in the general population, in whom the most common underlying causes of death are heart disease, then dementia, then lung cancer in men, and dementia, then heart disease, then stroke in women.[32] When all cancers are grouped together, in the general population, cancer is the leading underlying cause of death in 30% of men and 26% of women, compared with this study

For peer review only - http://bmjopen.bmj.com/site/about/guidelines.xhtml

reporting 0% for adults with Down syndrome, and 15.2% for adults with intellectual disabilities without Down syndrome – presumably as the adults with intellectual disabilities are dying younger from other causes, and cancers increase with age.

We found an overall SMR of 2.24; 5.28 in the adults with Down syndrome and 1.93 for the adults without Down syndrome. SMRs were higher for most ICD-10 chapter groupings of conditions. It was higher in the women than the men, as has been previously reported in most (supplementary table 1), but not all[10,19] previous reports. The reason for this is unknown; in the general population, mortality rates have fallen in recent decades, and more so in middle and older aged men than women (i.e. the sex gap is narrowing at these ages), but we do not know what trends over time there have been for people with intellectual disabilities. Having intellectual disabilities removes differences in lifespan by sex compared with the general population; but sex was not a predictor of mortality in our study, so the SMR difference may only be because of the difference found in the general population by sex. SMRs were lowest with older age groups, likely to be due to increased illness in the older general population and conversely a healthier group with intellectual disabilities living to older ages compared with those who die younger (as has previously been reported[33]). Although SMR was higher with increasing severity of intellectual disabilities, ability level was not retained within the multivariable model on time to death. The factors that were independently associated with increased risk of death, in order, were being percutaneous endoscopic gastrostomy/tube fed, Down syndrome, diabetes, having a lower respiratory tract infection at entry to the cohort, smoking, epilepsy, hearing impairment, total number of prescribed drugs, and age, whilst bowel incontinence had a reduced risk of death. Some of these predictors are similar to those reported in the general population, suggesting earlier mortality of adults with intellectual disabilities is largely accounted for by the higher rates of multi-morbidities that they experience compared with other people, and amenable deaths.[34]

For peer review only - http://bmjopen.bmj.com/site/about/guidelines.xhtml

Whilst accommodation type (not living with a family carer), ability level, not having day-time occupation, having spastic quadriplegia, visual impairment, constipation, ataxia/gait disorder, osteoporosis, hypertension, dysphagia, dyspnoea, gastro-oesophageal reflux disorder, total number of physical health disorders, not having impaired mobility, not having urinary incontinence, and not having autism, number of general practitioner consultations in the previous 12 months, total number of different types of health professionals providing care at the time of the health assessment, and antiepileptic drugs were related to time of death on univariate analyses, they were not retained in the multivariable model.

The majority of the adults with intellectual disabilities, with and without Down syndrome, died in an NHS hospital.

Comparison with previous literature

The overall SMR we report, higher SMR in women than men, and higher SMR at younger age groups is similar to the majority of previous reports. Most mortality studies with people with Down syndrome have been conducted with children. Previous reports of children and adults (combined) gave an SMR=5.5,[20] and for adults SMR=7.6,[9] compared with our finding for adults with Down syndrome of SMR=5.28. Recent systematic reviews reported people with intellectual disabilities on average died 20 years younger than other people, and people with Down syndrome died 28 years younger, although the majority of the Down syndrome studies were not recent.[1,2] In our study we found the gap between the age at death of people with intellectual disabilities with and without Down syndrome to be only 5.3 years, possibly reflecting the increasing lifespan of people with Down syndrome exceeding increases in lifespan for people with intellectual disabilities without Down syndrome. Notably, after "Down syndrome", dementia was the most commonly reported underlying, and all contributing cause of death for the adults with Down syndrome, whereas studies in the past commented on congenital heart disease and respiratory causes.

For peer review only - http://bmjopen.bmj.com/site/about/guidelines.xhtml

For the cohort overall, respiratory infection and aspiration/reflux/choking were the most common all contributing causes of death. These conditions feature in previous studies on causes of death,[5,6,8,10,11] although there are inconsistencies between studies. By ICD-10 chapter, our study found the most common underlying causes of death were diseases of the respiratory system, then of the circulatory system, followed by neoplasms. Others reported the most common to be vascular,[10] circulatory,[5] heart disease, [17] and jointly circulatory and neoplasm.[19]

Previous research from other countries has highlighted that listing Down syndrome or intellectual disabilities as the underlying cause of death obscures actual causes of death for this population.[35] We therefore presented data on revised cause of death for the 21 people for whom it was listed as Down syndrome (as a sensitivity check), and highlight with interest that in this Scottish cohort no-one had intellectual disabilities listed as underlying cause of death. This may reflect different medical death certificate recording practices in Scotland compared to e.g. USA.

Studies that investigated avoidable deaths in adults with intellectual disabilities found them to be more common than in the general population, due to deaths that would have been amenable to good care. Avoidable deaths have been reported in 44.7% of deaths of people with intellectual disabilities in England (mostly amenable deaths – figure not reported),[6] and in 31% in Australia,[19] compared with our figure of 38.9%. Avoidable deaths that would have been amenable to good care have been reported to occur in 37% of deaths of people with intellectual disabilities in England.[5] Our figure is slightly lower at 29.8% but still more than double that found in the Scottish general population.[31] It should be noted that the ONS list of avoidable deaths was not designed specifically for people with intellectual disabilities, and it may emphasise some causes less relevant, and omit others that might be highly relevant in this population.[5]

For peer review only - http://bmjopen.bmj.com/site/about/guidelines.xhtml

Strengths and limitations

The strengths of the study include the thorough methods of case ascertainment for intellectual disabilities at baseline with verification of intellectual disabilities and its severity, suggesting results are generalisable in other high income countries. Whist our Identification of the population will not have identified everyone with intellectual impairment (an IQ<70), in view of the multiple sources used, we believe it will have identified the adults with intellectual disabilities (IQ<70, plus need for support in daily activities, and onset in the developmental period). Additionally, there were detailed clinical assessments at baseline, and a longitudinal design. The size of the cohort and the duration of follow-up is also a strength, as is the successful record linkage for 94% of participants. Our study does have limitations, specifically that the study was only conducted in one region of Scotland, and the reliance upon death certificate data to obtain cause of death. Additionally, the characteristics and health of the participants was collected in 2002-2004. The health conditions we investigated tend to be long-standing or remitting/relapsing conditions, and psychotropic prescribing also once initiated tends to be long-standing in people with intellectual disabilities. However, it is possible that extent of neighbourhood deprivation, type of accommodation, employment, and civil status (though few marry) might have changed for some people between 2002-2004 and 2018; we have no further information to check this. There were no concerns regarding the proportional hazards assumption in the multivariable model. The linkage was also reliant on the accuracy of the CHI number as a sole source of linkage.

Implications

It is important to know the factors that are associated with risk of death, and the common causes of death in this population, as these then inform the actions needed to reduce the unacceptably high SMRs experienced by people with intellectual disabilities. Awareness of these factors may provide a pathway to action to reduce the observed earlier mortality in adults with intellectual disabilities. It is not adequate to solely rely on the public health interventions available to everyone, even when they are accessible.

For peer review only - http://bmjopen.bmj.com/site/about/guidelines.xhtml

Aspiration, reflux, and choking could, and should, be avoided by raising awareness of its consequences (death), and putting in place training on simple measures related to feeding, positioning, food consistency, and when to seek health advice from speech and language therapy, physiotherapy, nursing, and medical advice. Carers need to be aware of how the adults they care for express pain, so that conditions such as gastrointestinal ulcers are attended to, prior to the extreme point of perforation, and so treatable conditions such as constipation and urinary tract infections are managed before they lead to respiratory distress and sepsis. Quality of care is important; adults with intellectual disabilities need just as good care for their diabetes and epilepsy (and other conditions) as the rest of the population, with reasonable adjustments to address accessibility, and accessible smoking cessation programs.

Future research

Further research on larger samples is needed, particularly with regards to replicating and extending our findings on the factors that are associated with risk of death, and any sex differences in them, so that practitioners can focus on actions to improve the life expectancy of adults with intellectual disabilities, with and without Down syndrome.

References

1. O'Leary L, Hughes-McCormack L, Cooper S-A. Early death and causes of death of people with intellectual disabilities: A systematic review. *J Appl Res Intellect Disabil* 2017;31:325-42.
2. O'Leary L, Hughes-McCormack L, Cooper S-A. Life expectancy and causes of death of people with Down syndrome: a systematic review. *J Appl Res Intellect Disabil* 2018;31:687-708.
3. Cooper S-A, Hughes-McCormack L, Greenlaw N, *et al.* Management and prevalence of long-term conditions in primary health care for adults with intellectual disabilities

For peer review only - http://bmjopen.bmj.com/site/about/guidelines.xhtml

compared with the general population: a population-based cohort study. *J Appl Res Intellect Disabil* 2017;31(S1):68–81. 10.1111/jar.12386

4. Heslop P, Blair PS, Fleming P, *et al.* The Confidential Inquiry into premature deaths of people with intellectual disabilities in the UK: a population-based study. *Lancet* 2014;383:889–95.
5. Hosking FJ, Carey IM, Shah SM, *et al.* Mortality among adults with intellectual disability in England: Comparisons with the general population. *Am J Public Healt,* 2016;106:1483–90. ERRATUM: *Am J Public Health* 2016;106:e11–e12. doi: 10.2105/AJPH.2016.303240e
6. Glover G, Williams R, Heslop P, *et al.* Mortality in people with intellectual disabilities in England. *J Intellect Disabil Res* 2017;61:62-74.
7. Fosgren L, Edvinsson SO, Nystrom L, *et al.* Influence of epilepsy on mortality in mental retardation: an epidemiological study. *Epilepsia 1996;*37:956-63.
8. Durvasula S, Beange H, Baker W. Mortality of people with intellectual disability in northern Sydney. *J Intellect Development Disabil* 2002; 27:255-64.
9. Tyrer F, Smith LK, McGrother CW. Mortality in adults with moderate to profound intellectual disability: a population-based study. *J Intellectl Disabil Res* 2007;51:520–7.
10. Patja K, Mölsä P, Iivanainen M. Cause-specific mortality of people with intellectual disability in a population-based, 35-year follow-up study. *J Intellect Disabil Res* 201;45:30–40.
11. Tyrer F, McGrother C. Cause-specific mortality and death certificate reporting in adults with moderate to profound intellectual disability. *J Intellect Disabil Res* 2009;53:898–904.
12. Ouellette-Kuntz H, Shooshtari S, Balogh R, *et al.* Understanding information about mortality among people with intellectual and developmental disabilities in Canada. *J Appl Res Intellect Disabil* 2015;28:423–35.
13. Florio T, Trollor J. Mortality among a cohort of persons with an intellectual disability in New South Wales, Australia. *J Appl Res Intellect Disabil* 2015;28:383–93.

For peer review only - http://bmjopen.bmj.com/site/about/guidelines.xhtml

14. McCarron M, Carroll R, Kelly C, *et al.* Mortality rates in the general Irish population compared to those with an intellectual disability from 2003 to 2012. *J Appl Res Intellect Disabil* 2015;28:406–13.

15. Heslop P, Glover G. Mortality of people with intellectual disabilties in England: a comparison of data from existing sources. *J Appl Res Intellect Disabil* 2015;28:414-22.

16. Lauer E, McCallion P. Mortality of people with intellectual and developmental disabilities from select US state disability service systems and medical claims data. *J Appl Res Intellect Disabil* 2015;28:394-405.

17. Arvio M, Salokivi T, Tiitinen A, *et al.* Mortality in individuals with intellectual disabilities in Finland. *Brain Behavior* 2016;6:e00431.

18. Lauer E. 2012 & 2013 Mortality Report, Massachusetts. 2016; https://shriver.umassmed.edu/sites/shriver.umassmed.edu/files/2012-13%20DDS%20Mortality%20Report%20Final_v2.pdf [accessed 9.7.19].

19. Troller J, Srasuebkul P, Xu H, *et al.* Cause of death and potentially avoidable deaths in Australian adults with intellectual disability using retrospective linked data. *BMJOpen* 2017;7:e013489

20. Day SM, Strauss DJ, Shavelle RM, Reynolds RJ. Mortality and causes of death in persons with Down syndrome in California. *Developmental Med Child Neurology* 2005;47:171-6.

21. Kinnear D, Morrison J, Allan L, *et al.* The prevalence of multi-morbidity in a cohort of adults with intellectual disabilities, with and without Down syndrome. *BMJOpen* 2018; 0:e018292

22. World Health Organisation. *International Statistical Classification of Diseases and Related Health Problems, 10th Revision.* Geneva: World Health Organisation, 1992.

23. World Health Organisation. *The ICD-10 Classification of Mental and Behavioural Disorders: Diagnostic Criteria for Research.* Geneva: World Health organisation, 1993.

For peer review only

24. American Psychiatric Association. *Diagnostic and Statistical Manual of Mental Disorders, Fourth Edition Revised.* Washington, DC: American Psychiatric Association, 2000.

25. Royal College of Psychiatrists. *DC-LD [Diagnostic Criteria for Psychiatric Disorders for Use with Adults with Learning Disabilities / Mental Retardation],* London: Gaskell Press, 2001.

26. Cooper S-A, Smiley E, Morrison J, *et al.* Prevalence of and associations with mental ill-health in adults with intellectual disabilities. *Br J Psychiatry* 2007;190:27-35.

27. Finlayson J, Jackson A, Cooper S-A, *et al.* Understanding predictors of low physical exercise in adults with intellectual disabilities. *J Appl Res Intellect Disabil* 2009;22:236-47.

28. National Records of Scotland Vital Events Reference Tables 2010. Section 5: Deaths https://www.nrscotland.gov.uk/statistics-and-data/statistics/statistics-by-theme/vital-events/general-publications/vital-events-reference-tables/archive/2010/section-5-deaths

29. National Records of Scotland. Mid-2010 Population Estimates Scotland Population estimates by sex, age and administrative area. https://www.nrscotland.gov.uk/files/statistics/population-estimates/mid-2010/mid-year-pop-est-2010.pdf

30. Office for National Statistics Avoidable Mortality in England and Wales, 2013, Newport, South Wales. 2015. https://www.ons.gov.uk/peoplepopulationandcommunity/healthandsocialcare/causesofdeath/bulletins/avoidablemortalityinenglandandwales/2015

31. National Records of Scotland. Avoidable mortality. https://www.nrscotland.gov.uk/statistics-and-data/statistics/statistics-by-theme/vital-events/deaths/avoidable-mortality

32. Public Health England. Health profile for England. 2018 https://www.gov.uk/government/publications/health-profile-for-england-2018/chapter-2-trends-in-mortality

For peer review only - http://bmjopen.bmj.com/site/about/guidelines.xhtml

33. Landes SD. The Intellectual Disability Mortality Disadvantage: Diminishing with Age?. *Am J Intellect Developmental Disabil*, 2017;122:192-207.

34. Cooper S-A, McLean G, Guthrie B, *et al.* Multiple physical and mental health comorbidity in adults with intellectual disabilities: population-based cross-sectional analysis. *BMC Family Practice* 2015;DOI:10.1186/s12875-015-0329-3

35. Landes SD, Stevens JD, Turk MA. The obscuring effect of coding developmental disability as the underlying cause of death on mortality trends for adults with developmental disability: a cross-sectional study utilizing U.S. mortality data from 2012 to 2016. *BMJ Open* 2019; 9:e026614

Funding

This work was supported by the UK Medical Research Council, grant number: MC_PC_17217), and the Scottish Government via the Scottish Learning Disabilities Observatory.

The study sponsor and funders had no role in the study design; in the collection, analysis, and interpretation of data; in the writing of the report; and in the decision to submit the article for publication.

The researchers are independent from the funders.

Competing interests

The authors declare no competing interests.

Author's contributions

For peer review only - http://bmjopen.bmj.com/site/about/guidelines.xhtml

S-AC is principle investigator, she conceived and managed the project, interpreted data, and wrote the first draft of the manuscript. LA contributed to the conception of the project, and project management. NG designed and supervised the statistical analysis, and contributed to data interpretation and drafting of the manuscript. PMcS implemented and refined the statistical analysis, and contributed to data interpretation, and drafting of the manuscript. AJ implemented and refined the statistical analysis, and contributed to data interpretation. AH contributed to data linkage and interpretation, and drafting of the manuscript. CMcC provided expertise on data linkage and methods, and drafting of the manuscript. DK contributed to data interpretation and drafting of the manuscript. CM contributed to data interpretation, and drafting of the manuscript. All approved the final version of the manuscript. S-AC is the study guarantor.

Data sharing

Data is available via NHS GG&C Safe Haven upon application.

Acknowledgments

We thank the NHS Greater Glasgow and Clyde learning disabilities primary care liaison team for their contribution to the study, and the participants with intellectual disabilities and their carers.

For peer review only

For peer review only - http://bmjopen.bmj.com/site/about/guidelines.xhtml

Table 1. Cohort characteristics at time of the health assessment, summarised overall and by death status during the follow-up period

Variable	Statistics / Groups	All participants (N=961)	Deceased participants (N=294)	Alive participants (N=667)
Age (years)	Mean (SD)	44.1 (14.6)	52.4 (13.6)	40.5 (13.6)
	Min, max	16, 83	18, 83	16, 77
Age group	16-25 years	127 (13.2%)	10 (3.4%)	117 (17.5%)
	26-35 years	153 (15.9%)	26 (8.8%)	127 (19.0%)
	36-45 years	246 (25.6%)	49 (16.7%)	197 (29.5)
	46-55 years	205 (21.3%)	85 (28.8%)	120 (18.0%)
	>55 years	230 (23.9%)	124 (42.0%)	106 (15.9%)
Sex	Male	525 (54.6%)	154 (52.4%)	371 (55.6%)
	Female	436 (45.3%)	140 (47.5%)	296 (44.4%)
Ability level	Mild ID	382 (39.7%)	92 (31.2%)	290 (43.5%)
	Moderate ID	236 (24.5%)	73 (24.7%)	163 (24.4%)
	Severe ID	180 (18.7%)	67 (22.7%)	113 (16.9%)
	Profound ID	163 (17.0%)	62 (21.1%)	101 (15.1%)
Accommodation type	Family carer	374 (38.9%)	70 (23.8%)	304 (45.6%)
	Independent	93 (9.7%)	36 (12.2%)	57 (8.5%)
	Paid support	435 (45.2%)	161 (54.6%)	274 (41.1%)
	Congregate care	59 (6.1%)	27 (9.2%)	32 (4.8%)
Down syndrome	No	782 (81.4%)	230 (78.2%)	552 (82.8%)
	Yes	179 (18.6%)	64 (21.7%)	115 (17.2%)

ID=intellectual disabilities; SD=standard deviation

For peer review only

Table 2. Standardised mortality ratios

Variable	Groups	SMR (95% CI)
All participants	-	2.24 (1.99, 2.50)
Age group*	15-25 years	18.73 (0.37, 37.09)
	26-35 years	4.21 (1.29, 7.13)
	36-45 years	3.86 (2.28, 5.44)
	46-55 years	3.77 (2.90, 4.74)
	>55 years	1.86 (1.60, 2.12)
Sex	Male	1.69 (1.42, 1.95)
	Female	3.48 (2.90, 4.06)
Ability level	Mild ID	1.60 (1.27, 1.92)
	Moderate ID	2.10 (1.62, 2.58)
	Severe ID	2.78 (2.11, 3.44)
	Profound ID	4.14 (3.11, 5.17)
Down syndrome	No	1.93 (1.68, 2.18)
	Yes	5.28 (3.98, 6.57)
Underlying causes of death grouped by ICD-10 chapter**	Congenital malformations, deformations and chromosomal abnormalities	17.26 (10.75, 23.78)
	Diseases of the blood and blood-forming organs and certain disorders involving the immune mechanism	7.50 (-7.20, 22.20)
	Diseases of the circulatory system	5.55 (4.01, 7.09)
	Diseases of the digestive system	16.13 (8.23, 24.04)
	Diseases of the genitourinary system	3.65 (0.73, 6.57)
	Diseases of the musculoskeletal system and connective tissue	5.40 (-0.71, 11.52)
	Diseases of the nervous system	7.73 (5.13, 10.32)
	Diseases of the respiratory system	6.78 (5.02, 8.54)

For peer review only - http://bmjopen.bmj.com/site/about/guidelines.xhtml

For peer review only

	Diseases of the skin and subcutaneous tissue	2.75 (-2.64, 8.15)
	Endocrine, nutritional and metabolic diseases	3.43 (1.05, 5.81)
	External causes of morbidity and mobility	11.08 (3.40, 18.76)
	Infectious and parasitic diseases	8.93 (1.78, 16.07)
	Mental and behavioural disorders	12.64 (3.27, 22.00)
	Neoplasms	6.31 (4.19, 8.43)
	Symptoms, signs and abnormal clinical and laboratory findings, not elsewhere classified	19.51 (0.39, 38.63)

CI=confidence intervals; ID=intellectual disabilities; SMR=standardised mortality ratios

*Data used for comparison with General Population (GG&C Health Board) provides data in 5 year age bands therefore 15+. Data on adults with ID are 16+

** Negative Lower CI and wide CIs indicate low number of observed deaths in study population

For peer review only

For peer review only - http://bmjopen.bmj.com/site/about/guidelines.xhtml

Table 3. Underlying causes of death grouped by ICD-10 chapter, where cause of death is known

**ICD-10 chapter**	**Participants with Down syndrome (N=57)**	**Participants without Down syndrome (N=205)**
Certain infectious and parasitic diseases	5 (8.8%)	<5
Neoplasms	<5	33 (16.1%)
Diseases of the blood and blood-forming organs and certain disorders involving the immune mechanism	<5	<5
Endocrine, nutritional and metabolic diseases	<5	8 (3.9%)
Mental and behavioural disorders	5 (8.8%)	<5
Diseases of the nervous system	7 (12.3%)	27 (13.2%)
Diseases of the eye and adnexa	<5	<5
Diseases of the ear and mastoid process	<5	<5
Diseases of the circulatory system	8 (14.0%)	42 (20.5%)
Diseases of the respiratory system	8 (14.0%)	49 (23.9%)
Diseases of the digestive system	<5	16 (7.8%)
Diseases of the skin and subcutaneous tissue	<5	<5
Diseases of the musculoskeletal system and connective tissue	<5	<5
Diseases of the genitourinary system	<5	5 (2.4%)
Pregnancy, childbirth and the puerperium	<5	<5
Certain conditions originating in the perinatal period	<5	<5
Congenital malformations, deformations and chromosomal abnormalities	21 (36.8%)	6 (2.9%)
Symptoms, signs and abnormal clinical and laboratory findings, not elsewhere classified	<5	<5
External causes of morbidity and mortality	<5	7 (3.4%)
All deaths	57 (100%)	205 (100%)

ICD-10=International Statistical Classification of Diseases and Related Health Problems, 10th Revision

For peer review only - http://bmjopen.bmj.com/site/about/guidelines.xhtml

Table 4. Underlying causes of death grouped by specific individual causes or related groups of causes, where cause of death is known

Causes	Participants with Down syndrome (N=57)	Participants without Down syndrome (N=205)
Aspiration/reflux/choking	<5	22 (10.8%)
Respiratory infection	<5	21 (10.3%)
Down syndrome	21 (36.8%)	<5
Other malignancy	<5	19 (9.3%)
Other condition	<5	17 (8.3%)
Epilepsies	<5	13 (6.4%)
Acute myocardial infarction	<5	13 (6.4%)
Gastro-intestinal malignancy	<5	12 (5.9%)
Stroke	<5	11 (5.4%)
Other cardiovascular disease	<5	11 (5.4%)
Other respiratory condition	<5	9 (4.4%)
Other infection	5 (8.8%)	6 (2.9%)
Cerebral palsy	<5	11 (5.4%)
Dementia	9 (15.8%)	<5
Other gastrointestinal disorders	<5	8 (3.9%)
Ulcer/gastrointestinal perforation	<5	7 (3.4%)
Diabetes	<5	7 (3.4%)
Other congenital condition	<5	6 (2.9%)
Other ischaemic heart condition	<5	6 (2.9)
Mental health	<5	<5
Other neurological conditions	<5	<5
Renal failure	<5	<5
All deaths	57 (100%)	205 (100%)

For peer review only - http://bmjopen.bmj.com/site/about/guidelines.xhtml

Table 5. All contributing causes of death grouped by specific individual causes or related groups of causes, where cause of death is known

Causes	Participants with Down syndrome (N=57)	Participants without Down syndrome (N=205)
Respiratory infection	22 (38.6%)	49 (23.9%)
Aspiration/reflux/choking	11 (19.3%)	41 (20.0%)
Down syndrome	43 (75.4%)	<5
Other condition	8 (14.0%)	33 (16.1%)
Other cardiovascular disease	8 (14.0%)	30 (14.6%)
Other respiratory conditions	<5	31 (15.1%)
Other infection	9 (15.8%)	24 (11.7%)
Intellectual disabilities	<5	31 (15.1%)
Epilepsies	8 (14.0%)	24 (11.7%)
Dementia	24 (42.1%)	<5
Other neoplasms	<5	23 (11.2%)
Cerebral palsy	<5	24 (11.7%)
Acute myocardial infarction	5 (8.8%)	19 (9.3%)
Other gastrointestinal disorders	<5	18 (8.8%)
Diabetes	<5	19 (9.3%)
Other ischaemic heart disease	<5	19 (9.3%)
Renal failure	<5	16 (7.8%)
Stroke	<5	17 (8.3%)
Other congenital condition	<5	15 (7.3%)
Gastrointestinal malignant neoplasm	<5	12 (5.9%)
Ulcer/gastrointestinal perforation	<5	10 (4.9%)
Mental health	<5	10 (4.9%)
Other neurological condition	<5	8 (3.9%)
Heart failure	<5	7 (3.4%)
Injuries and accidents	<5	8 (3.9%)
Medical/surgical complications	<5	<5
Secondary malignancies	<5	<5
Thyroid disorders	<5	<5
Metabolic disorder	<5	<5
All deaths	57 (100%)	205 (100%)

For peer review only

For peer review only - http://bmjopen.bmj.com/site/about/guidelines.xhtml

Table 6. Multivariable model results for the outcome time to death

Variable		Hazard ratio	95% CI	p-value
Age at time of health assessment		1.056	1.046, 1.066	<0.0001
Smoker	No	1	-	
	Yes	1.531	1.1011, 2.128	0.0112
Down syndrome	No	1	-	
	Yes	2.440	1.787, 3.332	<0.0001
Epilepsy	No	1	-	
	Yes	1.511	1.173, 1.946	0.0014
Hearing impairment	No	1	-	
	Yes	1.320	1.030, 1.692	0.0284
Bowel incontinence	No	1	-	
	Yes	0.490	0.376, 0.640	<0.0001
Diabetes	No	1	-	
	Yes	2.346	1.553, 3.542	<0.0001
PEG/tube fed	No	1	-	
	Yes	2.346	1.135, 5.989	0.00240
Lower respiratory track infection	No	1	-	
	Yes	1.782	1.315, 2.415	0.0002
Total number of prescribed drugs		1.066	1.016, 1.118	0.0085

CI=confidence interval; PEG=percutaneous endoscopic gastrostomy

For peer review only

Rates, causes, place, and predictors of mortality in adults with intellectual disabilities with and without Down syndrome: cohort study with record linkage

Supplementary table 1. Previously reported standardised mortality ratios, causes, and risks for death

Author	Country	SMR (95% confidence interval)	Number of deaths	Causes of death and risk factors for death
Forsgren et al (1996)[7]	Sweden	4.2 (3.3, 5.3) at 20-59y; 1.1 (0.9, 1.5) at 60+y<br>*Without epilepsy:*<br>3.8 (2.8, 5.0) at 20-59y; 1.1 (0.8, 1.5) at 60+y<br>*With epilepsy:*<br>5.0 (2.9, 8.7) at 20-59y; 2.4 (0.9, 6.1) at 60+y<br>*With epilepsy and cerebral palsy:*<br>8.0 (4.1, 15.7) at 20-59y; 0.9 (0.1, 6.6) at 60+y<br>*M:* 1.6 (1.2, 2.0) at 0-60+y<br>*F:* 2.6 (2.0, 3.3) at 0-60+y<br>*Mild ID:* 1.8 (1.1, 2.7) at 0-60+y<br>*Moderate ID:* 1.5 (1.1, 2.0) at 0-60+y<br>*Severe ID:* 2.0 (1.5, 2.6) at 0-60+y<br>*Profound ID:* 8.1 (5.6, 11.7) at 0-60+y	124 at 0-60+y; 112 at 20-60+y	*Underlying cause at 0-60+y:*<br>Congenital anomalies: SMR=46.3 (32.9, 65.0)<br>Nervous system: SMR=9.7 (5.5, 17.0)<br>Mental disorder: SMR=4.0 (1.9, 8.4)<br>Respiratory: SMR=3.3 (2.0, 5.5)<br>Circulatory: SMR=2.1 (1.6, 2.7)<br>Violent death: SMR=1.4 (0.6, 2.8)<br>Neoplasm: SMR=0.9 (0.6, 1.6)
Durvasula & Beange (2002)[8]	Australia	4.9 (3.4, 6.4) at 10-59y<br>*M:* 4.1 (2.4, 5.9) at 10-59y<br>*F:* 6.2 (3.3, 9.1) at 10-59y	40 at 10-59y; 31 at 20-59y	*Underlying cause at 10-59y:*<br>Respiratory: 35% (pneumonia, aspiration)<br>External causes: 20%<br>Neoplasm: 17.5%<br>Heart disease: 15% (congenital heart disease 50%)<br>Gastrointestinal: 7.5% (ischaemic bowel, perforated peptic ulcer, post-operative peritonitis)<br>Seizure: 5%
Tyrer et al (2007)[9]	England	3.24 (2.93, 3.56) at 20-70+y<br>*M:* 2.86 (2.50, 3.26) at 20-70+y<br>*F:* 3.63 (3.12, 4.20) at 20-70+y<br>1.51 (1.23, 1.83) to 11.50 (8.14, 15.78) at 20-70+y<br>*M:* 1.39 (1.03, 1.82) to 8.83 (5.60, 13.25) at 20-70+y<br>*F:* 1.60 (1.18, 2.12) to 17.22 (9.64, 28.4) at 20-70+y<br>*With Down syndrome:* 7.60 at 20-70+y<br>*Without Down syndrome:* 2.70 at 20-70+y	409 at 20-70+y	Not reported
Patja et al (2008)[10]	Finland	*M:* 2.2 at 20-39y, 1.0 at 40-59y, 1.0 at 60+y<br>*F:* 1.4 at 20-39y, 1.1 at 40-49y, 1.0 at 60+y<br>*Mild ID:*<br>M: 1.6 at 20-39y, 1.0 at 40-59y, 1.0 at 60+y<br>F: 1.2 at 20-39y, 1.1 at 40-49y, 1.0 at 60+y	1,046 at 20-97y	*Underlying cause at 2-97y:*<br>Vascular: 36% (cardiac infarct 33%, cerebral infarct 33%, congenital heart disease 18%, pulmonary infarct 6%)<br>Respiratory: 22% (pneumonia 83%, COPD 11%)

For peer review only

Rates, causes, place, and predictors of mortality in adults with intellectual disabilities with and without Down syndrome: cohort study with record linkage

		Moderate ID:<br>M: 2.3 at 20-39y, 1.1 at 40-59y, 1.0 at 60+y<br>F: 1.5 at 20-39y, 1.1 at 40-49y, 1.0 at 60+y<br>*Severe ID:*<br>M: 2.6 at 20-39y, 1.2 at 40-59y, 1.0 at 60+y<br>F: 1.6 at 20-39y, 1.0 at 40-49y, 1.0 at 60+y<br>*Profound ID:*<br>M: 2.1 at 20-39y, 1.1 at 40-59y, 1.0 at 60+y<br>F: 1.3 at 20-39y, 1.2 at 40-49y, 1.0 at 60+y		Neoplasm: 11% (digestive 44%, respiratory 15%, urogenital, 12%)<br>Digestive: 7% (intestinal obstruction 25%, ulcer perforation 13%)<br>Accidents and poisonings: 7% (commonest was fatal fracture, then drowning)<br>Vascular, neoplasm, and accident causes were less common than sex-age-matched general population; Digestive were 2.5 times, Respiratory 2.6-5.8 times more common
Tyrer & McGother (2009)[11]	England	2.77 (2.53, 3.03) at 20+y<br>*M:* 2.28 (2.02, 2.56) at 20+y<br>*F:* 3.24 (2.83, 3.69) at 20+y	503 at 20+y	*Underlying cause at 20+y:*<br>Pneumonia: 13.1%, SMR=6.47 (5.00, 8.23)<br>Nervous system: 13.1%, SMR=16.30 (12.61, 20.74)<br>Other respiratory: 12.9%, SMR=4.64 (3.58, 5.91)<br>Ischaemic heart disease: 11.5%, SMR=1.49 (1.13, 1.92)<br>Neoplasm: 9.3%<br>Congenital anomalies: 9.1%, SMR=85.60 (62.67, 114.18)<br>Cerebrovascular disease: 7.8%, SMR=2.40 (1.71. 3.28)
Oullette-Kuntz et al (2015)[12]	Canada	2.5 (2.1, 2.9) at 0-60+y<br>*M:* 2.1 (1.7, 2.6) at 0-60+y<br>*F:* 3.0 (2.4, 3.8) at 0-60+y<br>*M:* 1.7 (1.3, 2.3) to 3.4 (2.3, 4.7) at 20-60+y<br>*F:* 2.1 (1.4, 2.9) to 6.1 (4.1, 8.6) at 20-60+y	172 at 0-60+y; 158 at 20-60+y	*Risk factors for death:*<br>Age, Down syndrome (OR=1.76 at 20-39y; OR=1.69 at 40-59y: OR=22.34 at 60+y), cerebral palsy (OR=2.39 at 20-39y; OR=0.93 at 40-59y: OR=0.50 at 60+y), blindness/low vision (OR not given), technological dependance/medical fragility (OR=11.96 at 20-39y; OR=7.28 at 40-59y: OR=3.42 at 60+y), wheelchair dependence (OR=5.96 at 20-39y; OR=2.89 at 40-59y: OR=2.56 at 60+y), mobility impairment without wheelchair dependence (OR not given), epilepsy (OR=1.83 at 20-39y; OR=1.80 at 40-59y: OR=1.09 at 60+y)
Florio & Troller (2015)[13]	Australia	2.48 (2.32, 2.64) at 0-85+y<br>3.15 (2.94, 3.38) at 5-69y<br>*M:* 2.52 (2.29, 2.77) at 5-69y<br>*F:* 4.26 (3.83, 4.74) at 5-69y	953 at 0-85+y; 831 at 15+y	Not reported
McCarron et al (2015)[14]	Republic of Ireland	3.85 (3.70, 4.00) at 0-80+y<br>*M:* 3.09 (2.93, 3.25) at 0-80+y<br>*F:* 4.90 (4.63, 5.17) at 0-80+y<br>2.71 (2.41, 3.04) to 6.09 (5.29, 6.96) at 20-80y<br>*M:* 2.50 (2.18, 2.86) to 4.50 (3.69, 5.44) at 20-80y<br>*F:* 2.71 (2.32, 3.14) to 10.07 (8.99, 13.10) at 20-80y	2,666 at 0-80+y; 2,394 at 20-80+y	Not reported

For peer review only

Rates, causes, place, and predictors of mortality in adults with intellectual disabilities with and without Down syndrome: cohort study with record linkage

Heslop & Glover (2015)[15]	England	Median 2.13 (interquartile range 1.09, 2.83) across geographic areas at 18-65+y	18-65+y	Not reported
Lauer & McCallion (2015)[16]	USA	*Intellectual and developmental disabilities**:*<br>1.19 at all ages, 2011<br>1.49 at 18+y, 2009	120,913 in 2009 at 18+y, 140,104 in 2011 at all ages	Not reported
Arvio et al (2016)[17]	Finland	*Mild ID:*<br>2.28 (2.18, 2.39) at 0-60+y<br>1.99 (1.85, 2.13) to 2.77 (2.36, 3.23) at 15-60+y<br>*M:* 2.01 (1.88, 2.14) at 0-60+y<br>*F:* 2.80 (2.60, 3.01) at 0-60+y<br>*Severe ID:*<br>3.41 (3.30, 3.52) at 0-60+y<br>2.07 (1.96, 2.19) to 8.77 (7.77, 9.87) at 15-60+y<br>*M:* 2.59 (2.48, 2.72) at 0-60+y<br>*F:* 5.24 (4.99, 5.50) at 0-60+y	5,171 at 0-60+y; 5,053 at 15-60y	Not reported
Hosking et al (2016)[5]	England	HR=3.62 (3.33, 3.93) at 18-84y<br>*M:* HR=3.30 (2.96, 3.68) at 18-84y<br>*F:* HR=4.10 (3.61, 4.66) at 18-84y<br>*With Down syndrome:* HR=9.21 (7.22, 11.76)<br>*Without Down syndrome:* HR=3.19 (2.92, 3.49)<br>*With epilepsy:* HR=6.04 (5.04, 7.24)<br>*Without epilepsy:* HR=3.18 (2.90, 3.50)<br>*With high level of support needs:* HR=4.77 (4.08, 5.59)<br>*Without high level of support needs:* HR=3.28 (2.98, 3.62)<br>*With autism:* HR=2.39 (1.45, 3.96)<br>*Without autism:* HR=3.66 (3.37, 3.98)<br>*In communal/shared homes:* HR=4.99 (4.36, 5.73)<br>*Not in communal/shared homes:* HR=3.05 (2.74, 3.30)	656 at 18-84y	*Underlying cause at 18-84y:*<br>Circulatory: 21.6%, HR=3.05 (2.56, 3.64)<br>Respiratory: 18.8% (pneumonia and aspiration pneumonia), HR=6.68 (5.38, 8.29)<br>Neoplasm: 14.9%, HR=1.44 (1.18, 1.76)<br>Nervous system: 11.6%, HR=13.79 (9.70, 19.62)<br>Digestive: 7.0%, HR=4.02 (2.92, 5.54)<br>Congenital anomalies: 6.9%, HR could not be estimated<br>Mental disorders: 5.3%, HR=7.99 (5.19, 12.31)<br>External causes: 4.1%, HR=1.85 (1.26, 2.71)<br>Genitourinary: 3.5%, HR=10.89 (6.09, 19.47)<br>Endocrine, nutritional, and metabolic: 2.0%, HR=5.38 (2.79, 10.07)<br>*Down syndrome:* Respiratory: 20.3% (or 42.4% if "Down syndrome" is excluded as an underlying cause of death)<br>*Avoidable deaths:*<br>37% amenable (23% controls), 19% preventable (40% controls)

For peer review only - http://bmjopen.bmj.com/site/about/guidelines.xhtml

Rates, causes, place, and predictors of mortality in adults with intellectual disabilities with and without Down syndrome: cohort study with record linkage

Lauer (2016)[18]	USA	Not reported	438 in 2012, 409 in 2013, at 18+y	*Major cause of death, 2012, 2013*<br>Heart disease: 16.0%, 13.7%<br>Neoplasm: 13.7%, 13.4%<br>Alzheimer disease: 13.0%-12.2% (48% in Down syndrome)<br>Aspiration pneumonia: 9.4%, 8.6%<br>Septicaemia: 10.0%, 8.6%<br>Chronic lower respiratory diseases: 4.6%, 6.6%<br>Unintentional injury: 4.8%, 3.2%
Troller et al (2017)[19]	Australia	1.3 (1.2, 1.5) at 20+y<br>4.0 (3.1, 5.2) at 20-44y<br>2.3 (2.0, 2.7) at 45-64y<br>1.0 (0.8, 1.20 at 65+y<br>*M:*1.4 (1.1, 1.6) at 20+y<br>*F:* 1.3 (1.1, 1.6) at 20+y	732 at 20-65+y	*Underlying cause at 20-65+y:*<br>Circulatory: 18%<br>Neoplasm: 18%<br>Nervous: 16%<br>Respiratory: 12%<br>Congenital anomaly: 11%<br>Injury and poisoning: 6%<br>Digestive: 5%<br>*Avoidable deaths:* 31%
Glover et al (2017)[6]	England	3.18 (2.94, 3.43) at 0-99y<br>*M:* 3.03 (2.73, 3.35) at 0-99y<br>*F:* 3.40 (3.02, 3.81) at 0-99y<br>1.6 (1.2, 2.1) to 7.8 (5.4, 11.1) at 18-99y<br>*M:* 1.5 (0.9, 2.2) to 6.6 (4.0, 10.1) at 18-99y<br>*F:* 1.7 (1.1, 2.4) to 11.6 (6.0, 20.2) at 18-99y	664 at 0-99y	*Underlying cause at 0-99y:*<br>Circulatory: 22.9% (ischaemic heart disease 37.5%, cerebrovascular 25.7%, thrombophlebitis 6.6%, cardiomyopathy 5.9%, PE 3.9%), SMR=2.8 (2.4, 3.3)<br>Respiratory: 17.2% (pneumonia 50.0%, pneumonitis 21.0%), SMR=4.9 (4.0, 5.9)<br>Neoplasm: 3.1% (digestive 36.8%, respiratory 13.8%, female genital tract 10.3%, lymphoid and haematopoietic 10.3%), SMR=1.1 (0.9, 1.4)<br>Nervous: 12.8%, SMR=9.8 (7.8, 12.1)<br>Congenital anomalies: 8.4%, SMR=72.9 (55.1, 94.7)<br>Digestive: 7.8%, SMR=4.0 (3.0, 5.2)<br>No ICD10 chapters had fewer than expected deaths<br>Other common single causes: dementia 33/664, epilepsy 26/664, cerebral palsy 23/664<br>*Avoidable deaths:*<br>44.7% (41.0%, 48.5%), mostly amenable<br>M: 50.9% (45.9%, 56.0%); F: 36.9% (31.5%, 42.5%)

COPD=chronic obstructive pulmonary disease; HR=hazard ratio; ID=intellectual disabilities; OR=odds ratio; PE=pulmonary embolism; SMR=standardised mortality ratio; y=years

*Includes some individuals with IQ>70

For peer review only

Rates, causes, place, and predictors of mortality in adults with intellectual disabilities with and without Down syndrome: cohort study with record linkage

For peer review only

Rates, causes, place, and predictors of mortality in adults with intellectual disabilities with and without Down syndrome: cohort study with record linkage

Supplementary table 2. Groupings of related causes of deaths

Infectious diseases	ICD10
**Infection**	
ENTEROCOLITIS DUE TO CLOSTRIDIUM DIFFICILE	A047
SEPSIS DUE TO STAPHYLOCOCCUS AUREUS	A410
SEPSIS, UNSPECIFIED	A419
BACTERIAL INFECTION, UNSPECIFIED	A499
SUBACUTE SCLEROSING PANENCEPHALITIS	A811
CHRONIC VIRAL HEPATITIS B WITHOUT DELTA-AGENT	B181
PULMONARY CANDIDIASIS	B371
NECROTISING FASCIITIS	M726
URINARY TRACT INFECTION, SITE NOT SPECIFIED	N390
**Neoplasms**	
**Gastrointestinal malignant neoplasms**	
MALIGNANT NEOPLASM OF PAROTID GLAND	C07
MALIGNANT NEOPLASM, OESOPHAGUS, UNSPECIFIED	C159
MALIGNANT NEOPLASM, STOMACH, UNSPECIFIED	C169
MALIGNANT NEOPLASM, CAECUM	C180
MALIGNANT NEOPLASM, SIGMOID COLON	C187
MALIGNANT NEOPLASM, COLON, UNSPECIFIED	C189
INTRAHEPATIC BILE DUCT CARCINOMA	C221
NEOPLASM OF UNCERTAIN OR UNKNOWN BEHAVIOUR, OTHER DIGESTIVE ORGANS	D377
**Other neoplasms**	
MALIGNANT NEOPLASM, LOWER LOBE, BRONCHUS OR LUNG	C343
MALIGNANT NEOPLASM, BRONCHUS OR LUNG, UNSPECIFIED	C349
MALIGNANT NEOPLASM, BREAST, UNSPECIFIED	C509
MALIGNANT NEOPLASM, ENDOMETRIUM	C541
MALIGNANT NEOPLASM OF OVARY	C56
MALIGNANT NEOPLASM, TESTIS, UNSPECIFIED	C629
MALIGNANT NEOPLASM, BLADDER, UNSPECIFIED	C679
MALIGNANT NEOPLASMS OF THYROID GLAND	C73
WALDENSTROM MACROGLOBULINAEMIA	C880
NON-HODGKIN'S LYMPHOMA, UNSPECIFIED	C859
MALIGNANT NEOPLASM OF UNSPECIFIED SITE	C80
NEOPLASM OF UNCERTAIN OR UNKNOWN BEHAVIOUR, TRACHEA, BRONCHUS AND LUNG	D381
SECONDARY MALIGNANT NEOPLASM OF LUNG	C780
SECONDARY MALIGNANT NEOPLASM OF LIVER AND INTRAHEPATIC BILE DUCT	C787
SECONDARY MALIGNANT NEOPLASM OF BRAIN AND CEREBRAL MENINGES	C793
SECONDARY MALIGNANT NEOPLASM OF OTHER SPECIFIED SITES	C798
**Endocrine and metabolic diseases**	
**Diabetes**	
INSULIN-DEPENDENT DIABETES MELLITUS WITHOUT COMPLICATIONS	E109
NON-INSULIN-DEPENDENT DIABETES MELLITUS WITH RENAL COMPLICATIONS	E112
NON-INSULIN-DEPENDENT DIABETES MELLITUS W. PERIPHERAL CIRC. COMPLICATIONS	E115
NON-INSULIN-DEPENDENT DIABETES MELLITUS WITHOUT COMPLICATIONS	E119
UNSPECIFIED DIABETES MELLITUS WITH RENAL COMPLICATIONS	E142

For peer review only

Rates, causes, place, and predictors of mortality in adults with intellectual disabilities with and without Down syndrome: cohort study with record linkage

UNSPECIFIED DIABETES MELLITUS WITHOUT COMPLICATIONS	E149
ABNORMAL GLUCOSE TOLERANCE TEST	R730
HYPERGLYCAEMIA, UNSPECIFIED	R739
**Metabolic disorders**	
OTHER HYPERPHENYLALANINAEMIAS	E701
DISORDERS OF PHOSPHORUS METABOLISM & PHOSPHATASES	E833
DISORDERS OF PLASMA-PROTEIN METABOLISM, NOT ELSEWHERE CLASSIFIED	E880
**Mental disorders**	
**Dementias**	
VASCULAR DEMENTIA, UNSPECIFIED	F019
UNSPECIFIED DEMENTIA	F03
ALZHEIMER'S DISEASE WITH LATE ONSET	G301
ALZHEIMER'S DISEASE, UNSPECIFIED	G309
**Mental health**	
MENTAL AND BEHAVIOURAL DISORDERS DUE TO ACUTE INTOXICATION WITH ALCOHOL	F100
MENTAL AND BEHAVIOURAL DISORDERS DUE TO ALCOHOL DEPENDENCE SYNDROME	F102
MENTAL AND BEHAVIOURAL DISORDERS DUE TO USE OF TOBACCO, UNSPECIFIED	F179
SCHIZOPHRENIA, UNSPECIFIED	F209
BIPOLAR AFFECTIVE DISORDER, UNSPECIFIED	F319
OTHER & UNSPEC SYMPTOMS & SIGNS INVOLVING COGNITIVE FUNCTIONS & AWARENESS	R418
INTENTIONAL SELF-HARM BY JUMPING FROM A HIGH PLACE	X80
**Intellectual disabilities**	
UNSPECIFIED MENTAL RETARDATION	F79
DEVELOPMENTAL DISORDER OF SCHOLASTIC SKILLS, UNSPECIFIED	F819
**Nervous system**	
**Epilepsies**	
GENERALIZED IDIOPATHIC EPILEPSY AND EPILEPTIC SYNDROMES	G403
EPILEPSY, UNSPECIFIED	G409
STATUS EPILEPTICUS, UNSPECIFIED	G419
MYOTONIC DISORDERS	G711
OTHER AND UNSPECIFIED CONVULSIONS	R568
**Cerebral palsy**	
SPASTIC QUADRAPLEGIC CEREBRAL PALSY	G800
SPASTIC HEMIPLEGIC CEREBRAL PALSY	G802
OTHER CEREBRAL PALSY	G808
CEREBRAL PALSY, UNSPECIFIED	G809
TETRAPLEGIA, UNSPECIFIED	G825
**Other neurological conditions**	
SEQUELAE OF INFLAMMATORY DISEASES OF CENTRAL NERVOUS SYSTEM	G09
PARKINSON'S DISEASE	G20
MYONEURAL DISORDER, UNSPECIFIED	G709
ENCEPHALITIS, MYELITIS AND ENCEPHALOMYELITIS, UNSPECIFIED	G049
ANOXIC BRAIN DAMAGE, NOT ELSEWHERE CLASSIFIED	G931
BLINDNESS, BINOCULAR	H540
OTHER DISORDERS OF NERVOUS SYSTEM, NOT ELSEWHERE CLASSIFIED	G98

For peer review only

Rates, causes, place, and predictors of mortality in adults with intellectual disabilities with and without Down syndrome: cohort study with record linkage

Circulatory system

Acute myocardial infarction

ACUTE MYOCARDIAL INFARCTION, UNSPECIFIED	I219
CARDIAC ARRECT, UNSPECIFIED	I469
**Other ischaemic heart disease**	
HYPERTENSIVE HEART DISEASE WITHOUT (CONGESTIVE) HEART FAILURE	I119
ACUTE ISCHAEMIC HEART DISEASE, UNSPECIFIED	I249
ATHEROSCLEROTIC HEART DISEASE	I251
CHRONIC ISCHAEMIC HEART DISEASE, UNSPECIFIED	I259
ATHEROSCLEROSIS OF AORTA	I700
GENERALIZED AND UNSPECIFIED ATHEROSCLEROSIS	I709
**Heart failure**	
HEART FAILURE, UNSPECIFIED	I509
LEFT VENTRICULAR FAILURE	I501
CONGESTIVE HEART FAILURE	I500
**Other cardiovascular disease**	
PULMONARY EMBOLISM WITHOUT MENTION OF ACUTE COR PULMONALE	I269
OTHER SPECIFIED PULMONARY HEART DISEASES	I278
PULMONARY HEART DISEASE, UNSPECIFIED	I279
AORTIC (VALVE) STENOSIS	I350
ATRIAL FIBRILLATION AND FLUTTER	I48
VENTRICULAR FIBRILLATION AND FLUTTER	I490
OTHER ILL-DEFINED HEART DISEASES	I518
PULMONARY OEDEMA	J81
CARDIOGENIC SHOCK	R570
PERIPHERAL VASCULAR DISEASE, UNSPECIFIED	I739
PHLEBITIS AND THROMBOPHLEBITIS OF OTHER DEEP VESSELS OF LOWER EXTREMITIES	I802
EMBOLISM AND THROMBOSIS OF OTHER SPECIFIED VEINS	I828
ACUTE AND SUBACUTE INFECTIVE ENDOCARDITIS	I330
ACUTE ENDOCARDITIS, UNSPECIFIED	I339
ENDOCARDITIS, VALVE UNSPECIFIED	I38
DILATED CARDIOMYOPATHY	I420
CARDIOMEGALY	I517
ESSENTIAL (PRIMARY) HYPERTENSION	I10
**Stroke**	
INTRACEREBRAL HAEMORRHAGE, UNSPECIFIED	I619
CEREBRAL INFARCTION DUE TO THROMBOSIS OF PRECEREBRAL ARTERIES	I630
CEREB INFARCT DUE TO UNSPEC OCCL/STENOSIS OF PRECEREB ARTERIES	I632
CEREBRAL INFARCTION, UNSPECIFIED	I639
STROKE, NOT SPECIFIED AS HAEMORRHAGE OR INFARCTION	I64
CEREBROVASCULAR DISEASE, UNSPECIFIED	I679
SEQUELAE OF STROKE, NOT SPECIFIED AS HAEMORRHAGE OR INFARCTION	I694
SEQUELAE OF OTHER AND UNSPECIFIED CEREBROVASCULAR DISEASES	I698

Respiratory system

Respiratory infection

ACUTE UPPER RESPIRATORY INFECTION, UNSPECIFIED	J069
INFLUENZA WITH PNEUMONIA, OTHER INFLUENZA VIRUS IDENTIFIED	J100
INFLUENZA WITH OTHER RESP MANIFESTATIONS, OTHER INFLUENZA VIRUS IDENTIFIED	J101

For peer review only

Rates, causes, place, and predictors of mortality in adults with intellectual disabilities with and without Down syndrome: cohort study with record linkage

PNEUMONIA DUE TO STREPTOCOCCUS PNEUMONIAE	J13
BRONCHOPNEUMONIA, UNSPECIFIED	J180
LOBAR PNEUMONIA, UNSPECIFIED	J181
HYPOSTATIC PNEUMONIA, UNSPECIFIED	J182
PNEUMONIA, UNSPECIFIED	J189
UNSPECIFIED ACUTE LOWER RESPIRATORY INFECTION	J22
CHRONIC OBSTRUCTIVE PULMONARY DISEASE WITH ACUTE LOWER RESP INFECTION	J440
**Aspiration/reflux/choking**	
PNEUMONITIS DUE TO FOOD AND VOMIT	J690
GASTRO-OESOPHAGEAL REFLUX DISEASE WITHOUT OESOPHAGITIS	K219
INHALATION AND INGESTION OF FOOD CAUSING OBSTRUCTION OF RESPIRATORY TRACT	W79
FOREIGN BODY IN RESPIRATORY TRACT, PART UNSPECIFIED	T179
INHALATION/INGESTION OF OTHER OBJECTS CAUSING OBSTRUCT OF RESP TRACT	W80
**Other respiratory disorders**	
UNSPECIFIED CHRONIC BRONCHITIS	J42
EMPHYSEMA, UNSPECIFIED	J439
CHRONIC OBSTRUCTIVE PULMONARY DISEASE, UNSPECIFIED	J440
ASTHMA, UNSPECIFIED	J459
BRONCHIECTASIS	J47
OTHER INTERSTITIAL PULMONARY DISEASES WITH FIBROSIS	J841
PLEURAL EFFUSION, NOT ELSEWHERE CLASSIFIED	J90
CHRONIC RESPIRATORY FAILURE	J961
RESPIRATORY FAILURE, UNSPECIFIED	J969
OTHER SPECIFIED RESPIRATORY DISORDERS	J988
DYSPNOEA	R060
RESPIRATORY ARREST	R092
ASPHYXIATION	T71
UNSPECIFIED THREAT TO BREATHING	W84
**Digestive system**	
**Ulcer/gastrointestinal perforation**	
OESOPHAGITIS	K20
PERFORATION OF INTESTINE (NONTRAUMATIC)	K631
PERITONITIS, UNSPECIFIED	K659
GASTRIC ULCER, CHRONIC OR UNSPECIFIED WITH PERFORATION	K255
OTHER PERITONITIS	K658
ACUTE PERITONITIS	K650
GASTROINTESTINAL HAEMORRHAGE, UNSPECIFIED	K922
ULCER OF INTESTINE	K633
**Other gastrointestinal disorders**	
BARRETTS OESOPHAGUS	K227
DIAPHRAGMATIC HERNIA WITHOUT OBSTRUCTION OR GANGRENE	K449
OTHER SPECIFIED NONINFECTIVE GASTROENTERITIS AND COLITIS	K528
ACUTE VASCULAR DISORDERS OF INTESTINE	K550
VASCULAR DISORDER OF INTESTINE, UNSPECIFIED	K559
VOLVULUS	K562
OTHER AND UNSPECIFIED INTESTINAL OBSTRUCTION	K566
CONSTIPATION	K590

For peer review only

Rates, causes, place, and predictors of mortality in adults with intellectual disabilities with and without Down syndrome: cohort study with record linkage

MEGACOLON, NOT ELSEWHERE CLASSIFIED	K593
ACUTE AND SUBACUTE HEPATIC FAILURE	K720
OTHER AND UNSPECIFIED CIRRHOSIS OF LIVER	K746
AUTOIMMUNE HEPATITIS	K754
INFLAMMATORY LIVER DISEASE, UNSPECIFIED	K759
OTHER SPECIFIED DISEASES OF LIVER	K768
CALCULUS OF GALLBLADDER WITH OTHER CHOLECYSTITIS	K801
CHOLANGITIS	K830
ACUTE PANCREATITIS, UNSPECIFIED	K859
PSEUDOCYST OF PANCREAS	K863
INTESTINAL MALABSORPTION, UNSPECIFIED	K909
DYSPHAGIA	R13

Genitourinary system

Renal failure

CHRONIC NEPHRITIC SYNDROME, UNSPECIFIED	N039
OTHER ACUTE RENAL FAILURE	N178
ACUTE RENAL FAILURE, UNSPECIFIED	N179
END-STAGE RENAL DISEASE	N180
CHRONIC KIDNEY DISEASE, STAGE 5	N185
CHRONIC KIDNEY DISEASE, UNSPECIFIED	N189
UNSPECIFIED KIDNEY FAILURE	N19

Chromosomal abnormalities

Down syndrome

DOWN'S SYNDROME, UNSPECIFIED	Q909

Other congenital condition

CONGENITAL HYDROCEPHALUS, UNSPECIFIED	Q039
SPINA BIFIDA, UNSPECIFIED	Q059
CONGENITAL MALFORMATION OF HEART, UNSPECIFIED	Q249
CONGENITAL DEFORMITY OF SPINE	Q675
CONGEN MALFORMATION SYNDROMES PREDOMINANTLY ASSOCIATED WITH SHORT STATURE	Q871
MARFAN'S SYNDROME	Q874
OTHER SPECIFIED CONGEN MALFORMATION SYNDROMES, NOT ELSEWHERE CLASSIFIED	Q878
CONGENITAL MALFORMATION, UNSPECIFIED	Q899
KLINEFELTER'S SYNDROME, UNSPECIFIED	Q984
FRAGILE X CHROMOSOME	Q992
OTHER LACK OF EXPECTED NORMAL PHYSIOLOGICAL DEVELOPMENT	R628

Other conditions occurring with small frequency

Other condition

DECUBITUS ULCER AND PRESSURE AREA	L89
SCOLIOSIS, UNSPECIFIED	M419
URETHRAL STRICTURE, UNSPECIFIED	N359
EPISTAXIS	R040
IMMOBILITY	R263
MALAISE AND FATIGUE	R53
GENERALIZED ENLARGED LYMPH NODES	R591

For peer review only

Rates, causes, place, and predictors of mortality in adults with intellectual disabilities with and without Down syndrome: cohort study with record linkage

INSUFFICIENT INTAKE OF FOOD AND WATER DUE TO SELF NEGLECT	R636
OTHER SPECIFIED GENERAL SYMPTOMS AND SIGNS	R688
OTHER ILL-DEFINED AND UNSPECIFIED CAUSES OF MORTALITY	R99
EXPOSURE TO UNSPECIFIED FACTOR	X59
MULTI-SYSTEM DEGENERATION	G903
BENIGN NEOPLASM, MENINGES, UNSPECIFIED	D329
AGRANULOCYTOSIS	D70
SARCOIDOSIS OF OTHER AND COMBINED SITES	D868
SARCOIDOSIS, UNSPECIFIED	D869
HYPOPITUITARISM	E230
HYPOTHYROIDISM, UNSPECIFIED	E039
OTHER THYROTOXICOSIS	E058
VOLUME DEPLETION	E86
**Injuries and external causes**	
**Injuries and accidents**	
INTRACRANIAL INJURY, UNSPECIFIED	S069
UNSPECIFIED INJURY OF HEAD	S099
INJURY OF COLON	S365
FRACTURE OF NECK OF FEMUR	S720
FRACTURE OF SHAFT OF TIBIA	S822
UNSPECIFIED MULTIPLE INJURIES	T07
FAT EMBOLISM (TRAUMATIC)	T791
SEQUELAE OF UNSPECIFIED INJURY OF HEAD	T909
UNSPECIFIED FALL	W19
SEQUELAE OF OTHER ACCIDENTS	Y86
**Medical/surgical complication**	
POISONING BY OTHER ANTIDYSRHYTHMIC DRUGS, NOT ELSEWHERE CLASSIFIED	T462
ADVERSE EFFECTS OF OTHER ANTIDYSRHYTHMIC DRUGS, NOT ELSEWHERE CLASSIFIED	Y522
ABN REACT TO/LATER COMPLIC OF OP WITH IMPLANT OF ARTIFICIAL INTERN DEVICE	Y831
ABN REACT TO/LATER COMPLIC OF OP WITH ANASTOMOSIS, BYPASS OR GRAFT	Y832
ABN REACT TO/LATER COMPLIC OF OP WITH FORMATION OF EXTERNAL STOMA	Y833
ABNORMAL REACTION TO OR LATER COMPLICATION OF OTHER MEDICAL PROCEDURES	Y848
SEQ OF PROCED CAUSING ABN REACT/COMPLIC,W/O MENTION OF MISADV AT THE TIME	Y883
OTHER POSTPROCEDURAL RESPIRATORY DISORDERS	J958

For peer review only

Rates, causes, place, and predictors of mortality in adults with intellectual disabilities with and without Down syndrome: cohort study with record linkage

Supplementary table 3. Predictors of the outcome time to death from univariate analyses

Variable		N with event/ N in group	Hazard ratio (95% CI)	Individual p-value	Overall p-value
Demographics					
Age at time of health assessment		294/961	1.05 (1.04, 1.06)	<0.0001	
Sex	Male	154/525	0.88 (0.70, 1.11)	0.2730	
	Female	140/436	1.00 (-)		
Ability level	Mild ID	92/382	1.00 (-)		0.0007
	Moderate ID	73/236	1.38 (1.01, 1.87)	0.0411	
	Severe ID	67/180	1.75 (1.28, 2.40)	0.0005	
	Profound ID	62/163	1.77 (1.28, 2.45)	0.0005	
Type of accommodation	Family carer	70/374	1.00 (-)		<0.0001
	Independent of care	36/93	2.35 (1.57, 3.52)	<0.0001	
	Paid support	161/435	2.18 (1.65, 2.88)	<0.0001	
	Congregate	27/59	2.87 (1.84, 4.48)	<0.0001	
Neighbourhood deprivation	1 – most affluent	18/73	1.00 (-)		0.1890
	2	56/137	1.92 (1.13, 3.27)	0.0158	
	3	10/45	0.90 (0.42, 1.95)	0.7896	
	4	10/40	1.06 (0.49, 2.30)	0.8808	
	5	12/32	1.71 (0.82, 3.55)	0.1527	
	6	9/32	1.27 (0.57, 2.82)	0.5640	
	7	9/34	1.09 (0.49, 2.43)	0.8302	
	8	15/58	1.21 (0.61, 2.41)	0.5818	
	9	35/124	1.22 (0.69, 2.16)	0.4882	
	10 - most deprived	120/386	1.41 (0.86, 2.31)	0.1782	
Civil status	Single	288/938	1.28 (0.57, 2.87)	0.5485	
	Not single	6/23	1.00 (-)		
Employment/day activities	Yes	83/231	1.33 (1.03, 1.71)	0.0284	
	No	211/730	1.00 (-)		
Smoker	Yes	46/101	1.70 (1.24, 2.33)	0.0009	
	No	248/860	1.00 (-)		
**Health**					
Down syndrome	Yes	64/179	1.30 (0.98, 1.71)	0.0673	
	No	230/782	1.00 (-)		
Epilepsy	Yes	111/325	1.25 (0.99, 1.58)	0.0636	
	No	183/636	1.00 (-)		
Spastic quadriplegia	Yes	24/325	1.67 (1.10, 2.54)	0.0158	
	No	183/636	1.00 (-)		
Impaired mobility	Yes	195/735	0.51 (0.40, 0.65)	<0.0001	
	No	99 /226	1.00 (-)		
Body mass index	Underweight	9/43	0.63 (0.32, 1.25)	0.1847	0.1865
	Acceptable	83/265	1.00 (-)		
	Overweight	75/289	0.78 (0.57, 1.06)	0.1132	
	Obese	81/237	1.08 (0.80, 1.47)	0.6152	
	Morbidly obese	16/58	0.87 (0.51, 1.48)	0.6058	
Hearing impairment	Yes	112/267	1.79 (1.41, 2.26)	<0.0001	
	No	182/694	1.00 (-)		
Visual impairment	Yes	154/449	1.29 (1.02, 1.62)	0.0317	
	No	140/512	1.00 (-)		

For peer review only

Urinary incontinence	Yes	158/632	0.52 (0.41, 0.65)	<0.0001	
	No	136/329	1.00 (-)		
Bowel incontinence	Yes	197/733	0.55 (0.43, 0.70)	<0.0001	
	No	97/228	1.00 (-)		
Diabetes	Yes	29/47	2.72 (1.86, 4.00)	<0.0001	
	No	265/914	1.00 (-)		
PEG/tube fed	Yes	N/7	4.99 (2.22, 11.20)	0.0001	
	No	288/954			
Constipation	Yes	112/316	1.34 (1.06, 1.70)	0.0145	
	No	182/645	1.00 (-)		
Ataxia/gait disorder	Yes	104/276	1.50 (1.18, 1.90)	0.0009	
	No	190/685	1.00 (-)		
Nail disorder	Yes	74/223	1.18 (0.91, 1.54)	0.2120	
	No	220/738	1.00 (-)		
Epidermal thickening	Yes	66/207	1.10 (0.84, 1.45)	0.4947	
	No	228/754	1.00 (-)		
Cerebral palsy	Yes	54/175	1.02 (0.76, 1.37)	0.8792	
	No	240/786	1.00 (-)		
Osteoporosis	Yes	76/174	1.71 (1.32, 2.22)	<0.0001	
	No	218/786	1.00 (-)		
Fungal infection	Yes	42/158	0.83 (0.61, 1.18)	0.3366	
	No	252/803	1.00 (-)		
Hypertension	Yes	56/146	1.36 (1.01, 1.82)	0.0399	
	No	238/815	1.00 (-)		
Dysphagia	Yes	51/132	1.51 (1.11, 2.04)	0.0080	
	No	243/829	1.00 (-)		
Dyspnoea	Yes	49/130	1.41 (1.04, 1.92)	0.0285	
	No	245/831	1.00 (-)		
Muskuloskeletal pain	Yes	48/148	1.14 (0.83, 1.55)	0.4153	
	No	246/813	1.00 (-)		
Bone deformity	Yes	50/139	1.32 (0.97, 1.79)	0.0769	
	No	244/822	1.00 (-)		
Dental/oral problem	Yes	38/120	1.07 (0.76, 1.50)	0.7128	
	No	256/841	1.00 (-)		
Eczema/dermatitis	Yes	38/138	0.86 (0.61, 1.21)	0.3790	
	No	256/823	1.00 (-)		
GORD	Yes	51/133	1.43 (1.06, 1.94)	0.0198	
	No	243/828	1.00 (-)		
Lower respiratory tract infection	Yes	55/126	1.75 (1.30, 2.34)	0.0002	
	No	239/835	1.00 (-)		
Total number of physical conditions		294/961	1.06 (1.04, 1.08)	<0.0001	
Psychosis	Yes	11 /42	0.81 (0.44, 1.48)	0.4990	
	No	283 /919	1.00 (-)		
Affective disorder including bipolar	Yes	24/68	1.19 (0.78, 1.80)	0.4216	
	No	270/893	1.00 (-)		
Autism	Yes	13/69	0.54 (0.31, 0.94)	0.0306	
	No	281/892	1.00 (-)		
Problem behaviour	Yes	71/218	1.09 (0.83, 1.42)	0.5251	
	No	223/743	1.00 (-)		
Eating disorder, including pica	Yes	5/17	0.99 (0.41, 2.40)	0.9857	
	No	289/944	1.00 (-)		
Any mental illness, excluding problem behaviours	Yes	73/217	1.16 (0.89, 1.51)	0.2849	
	No	221/744	1.00 (-)		
Service use					
Number of GP consultations in last 12 months		287/951	1.05 (1.03, 1.06)	<0.0001	

For peer review only

Rates, causes, place, and predictors of mortality in adults with intellectual disabilities with and without Down syndrome: cohort study with record linkage

Number of A&E attendances in last 12 months		280/938	1.09 (0.99, 1.20)	0.0847	
Number of health professions providing care		294 /961	1.10 (1.03, 1.16)	0.0023	
Prescriptions					
Antipsychotics	Yes	79/226	1.12 (0.94, 1.57)	0.1421	
	No	215/735	1.00 (-)		
Antidepressants	Yes	39/118	1.16 (0.83, 1.63)	0.3778	
	No	255/843	1.00 (-)		
Anxiolytic/hypnotics	Yes	20/68	0.95 (0.60, 1.49)	0.8159	
	No	274/893	1.00 (-)		
Antiepileptics	Yes	90/253	1.31 (1.02, 1.68)	0.0315	
	No	204/708	1.00 (-)		
Number of drug classes taken		294/961	1.16 (1.12, 1.21)	<0.0001	

A&E=accident and emergency; CI=confidence interval; GORD=gastro-oesophageal reflux disorder; PEG=percutaneous endoscopic gastrostomy

For peer review only

STROBE Statement—checklist of items that should be included in reports of observational studies

	Item No.	Recommendation	Page No.	Relevant text from manuscript
**Title and abstract**	1	(*a*) Indicate the study's design with a commonly used term in the title or the abstract		p1
		(*b*) Provide in the abstract an informative and balanced summary of what was done and what was found		p2
**Introduction**				
Background/rationale	2	Explain the scientific background and rationale for the investigation being reported		p4-6, supplementary table 1
Objectives	3	State specific objectives, including any prespecified hypotheses		6, paragraph 3
**Methods**				
Study design	4	Present key elements of study design early in the paper		p6-10, supplementary tables 2/3
Setting	5	Describe the setting, locations, and relevant dates, including periods of recruitment, exposure, follow-up, and data collection		p7, paragraph 1, 7-8
Participants	6	(*a*) *Cohort study*—Give the eligibility criteria, and the sources and methods of selection of participants. Describe methods of follow-up<br>*Case-control study*—Give the eligibility criteria, and the sources and methods of case ascertainment and control selection. Give the rationale for the choice of cases and controls<br>*Cross-sectional study*—Give the eligibility criteria, and the sources and methods of selection of participants		p7, paragraph 1
		(*b*) *Cohort study*—For matched studies, give matching criteria and number of exposed and unexposed<br>*Case-control study*—For matched studies, give matching criteria and the number of controls per case		p7, paragraph 2, p9, paragraph 4
Variables	7	Clearly define all outcomes, exposures, predictors, potential confounders, and effect modifiers. Give diagnostic criteria, if applicable		p7-8, supplementary table 2
Data sources/ measurement	8*	For each variable of interest, give sources of data and details of methods of assessment (measurement). Describe comparability of assessment methods if there is more than one group		p7-8, p9, paragraph 4
Bias	9	Describe any efforts to address potential sources of bias		p9, paragraph 4
Study size	10	Explain how the study size was arrived at		P7, paragraph2, p9, paragraph 4

Continued on next page

For peer review only

Quantitative variables	11	Explain how quantitative variables were handled in the analyses. If applicable, describe which groupings were chosen and why	p8-10
Statistical methods	12	(*a*) Describe all statistical methods, including those used to control for confounding	p8-10
		(*b*) Describe any methods used to examine subgroups and interactions	p8-10
		(*c*) Explain how missing data were addressed	p11, paragraph 2
		(*d*) *Cohort study*—If applicable, explain how loss to follow-up was addressed<br>*Case-control study*—If applicable, explain how matching of cases and controls was addressed<br>*Cross-sectional study*—If applicable, describe analytical methods taking account of sampling strategy	p11, paragraph 2
		(*e*) Describe any sensitivity analyses	N/A
**Results**			
Participants	13*	(a) Report numbers of individuals at each stage of study—eg numbers potentially eligible, examined for eligibility, confirmed eligible, included in the study, completing follow-up, and analysed	p11, paragraph 2
		(b) Give reasons for non-participation at each stage	p11, paragraph 2
		(c) Consider use of a flow diagram	-
Descriptive data	14*	(a) Give characteristics of study participants (eg demographic, clinical, social) and information on exposures and potential confounders	p11-12, Table 1, supplementary table 3
		(b) Indicate number of participants with missing data for each variable of interest	table 1, supplementary table 3
		(c) *Cohort study*—Summarise follow-up time (eg, average and total amount)	p12, paragraph 1
Outcome data	15*	*Cohort study*—Report numbers of outcome events or summary measures over time	p11, paragraph 2
		Case-control study—Report numbers in each exposure category, or summary measures of exposure	
		Cross-sectional study—Report numbers of outcome events or summary measures	
Main results	16	(*a*) Give unadjusted estimates and, if applicable, confounder-adjusted estimates and their precision (eg, 95% confidence interval). Make clear which confounders were adjusted for and why they were included	P15, table 6
		(*b*) Report category boundaries when continuous variables were categorized	N/A
		(*c*) If relevant, consider translating estimates of relative risk into absolute risk for a meaningful time period	-

Continued on next page

For peer review only

Other analyses	17	Report other analyses done—eg analyses of subgroups and interactions, and sensitivity analyses	p11-16
**Discussion**			
Key results	18	Summarise key results with reference to study objectives	p16, paragraph 2
Limitations	19	Discuss limitations of the study, taking into account sources of potential bias or imprecision. Discuss both direction and magnitude of any potential bias	p20, paragraph 1
Interpretation	20	Give a cautious overall interpretation of results considering objectives, limitations, multiplicity of analyses, results from similar studies, and other relevant evidence	p20, paragraph 2
Generalisability	21	Discuss the generalisability (external validity) of the study results	p20, paragraph 1
**Other information**			
Funding	22	Give the source of funding and the role of the funders for the present study and, if applicable, for the original study on which the present article is based	P24

*Give information separately for cases and controls in case-control studies and, if applicable, for exposed and unexposed groups in cohort and cross-sectional studies.

Note: An Explanation and Elaboration article discusses each checklist item and gives methodological background and published examples of transparent reporting. The STROBE checklist is best used in conjunction with this article (freely available on the Web sites of PLoS Medicine at http://www.plosmedicine.org/, Annals of Internal Medicine at http://www.annals.org/, and Epidemiology at http://www.epidem.com/). Information on the STROBE Initiative is available at www.strobe-statement.org.

For peer review only
